# Robustness of MEK-ERK Dynamics and Origins of Cell-to-Cell Variability in MAPK Signaling

**DOI:** 10.1016/j.celrep.2016.05.024

**Published:** 2016-06-02

**Authors:** Sarah Filippi, Chris P. Barnes, Paul D.W. Kirk, Takamasa Kudo, Katsuyuki Kunida, Siobhan S. McMahon, Takaho Tsuchiya, Takumi Wada, Shinya Kuroda, Michael P.H. Stumpf

**Affiliations:** 1 Centre for Integrative Systems Biology and Bioinformatics, Imperial College London, London SW7 2AZ, UK; 2 Department of Cell and Developmental Biology, University College London, London WC1E 6BT, UK; 3 Department of Genetics, Evolution and Environment, University College London, London WC1E 6BT, UK; 4 Department of Biological Sciences, Graduate School of Science, University of Tokyo, Tokyo 113-8654, Japan; 5 CREST, Japan Science and Technology Agency, Bunkyo-ku, Tokyo 113-0033, Japan; 6 Institute of Chemical Biology, Imperial College London, London SW7 2AZ, UK

## Abstract

Cellular signaling processes can exhibit pronounced cell-to-cell variability in genetically identical cells. This affects how individual cells respond differentially to the same environmental stimulus. However, the origins of cell-to-cell variability in cellular signaling systems remain poorly understood. Here, we measure the dynamics of phosphorylated MEK and ERK across cell populations and quantify the levels of population heterogeneity over time using high-throughput image cytometry. We use a statistical modeling framework to show that extrinsic noise, particularly that from upstream MEK, is the dominant factor causing cell-to-cell variability in ERK phosphorylation, rather than stochasticity in the phosphorylation/dephosphorylation of ERK. We furthermore show that without extrinsic noise in the core module, variable (including noisy) signals would be faithfully reproduced downstream, but the within-module extrinsic variability distorts these signals and leads to a drastic reduction in the mutual information between incoming signal and ERK activity.

## Highlights

• Active MEK and ERK levels differ profoundly among genetically identical cells
• A statistical framework is developed to identify the causes of this variability
• Analysis shows that extrinsic noise upstream MEK-ERK module causes cell variability
• Within-module extrinsic variability distorts signals

## Graphical Abstract

**Figure:**
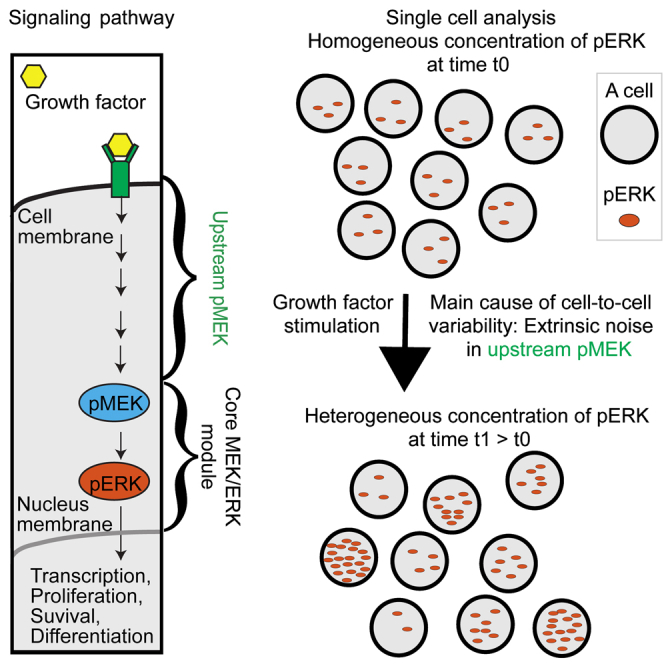

## Teaser

Cellular signaling processes can exhibit pronounced cell-to-cell variability in genetically identical cells, but the origins of such variability remain poorly understood. Filippi et al. present a comprehensive analysis of cell-to-cell variability in the ERK phosphorylation process by combining a statistical modeling approach with high-throughput image cytometry measurements.

## Introduction

The behavior of eukaryotic cells is determined by an intricate interplay between signaling, gene regulation, and epigenetic processes. Within a cell, each single molecular reaction occurs stochastically, and the expression levels of molecules can vary considerably in individual cells (Bowsher and Swain, 2012). These non-genetic differences frequently add up to macroscopically observable phenotypic variation (Spencer et al., 2009, Balázsi et al., 2011, Spiller et al., 2010). Such variability can have organism-wide consequences, especially when small differences in the initial cell populations are amplified among their progeny (Quaranta and Garbett, 2010, Pujadas and Feinberg, 2012). Cancer is the canonical example of a disease caused by a sequence of chance events that may be the result of amplifying physiological background levels of cell-to-cell variability (Roberts and Der, 2007).

Better understanding of the molecular mechanisms behind the initiation, enhancement, attenuation, and control of this cellular heterogeneity should help us to address a host of fundamental questions in cell biology and experimental and regenerative medicine. Noise at the molecular level has been amply demonstrated in the literature, in the contexts of both gene expression (Elowitz et al., 2002, Swain et al., 2002, Hilfinger and Paulsson, 2011) and signal transduction (Colman-Lerner et al., 2005, Jeschke et al., 2013). The molecular causes underlying population heterogeneity are only beginning to be understood, and each new study adds nuance and detail to our emerging understanding. Two notions have come to dominate the literature: intrinsic and extrinsic causes of cell-to-cell variability (Swain et al., 2002, Komorowski et al., 2010, Hilfinger and Paulsson, 2011, Toni and Tidor, 2013, Bowsher and Swain, 2012). The former refers to the chance events governing the molecular collisions in biochemical reactions. Each reaction occurs at a random time leading to stochastic differences between cells over time. The latter subsumes all those aspects of the system that are not explicitly modeled. This includes the impact of stochastic dynamics in any components upstream and/or downstream of the biological system of interest, which may be caused, for example, by the stage of the cell cycle and the multitude of factors deriving from it.

It has now become possible to track populations of eukaryotic cells at single-cell resolution over time and measure the changes in the abundances of proteins (Selimkhanov et al., 2014). For example, rich temporal behavior of p53 (Geva-Zatorsky et al., 2006, Batchelor et al., 2011) and Nf-κb (Nelson et al., 2004, Ashall et al., 2009, Paszek et al., 2010) has been characterized in single-cell time-lapse imaging studies. Given such data, and with a suitable model for system dynamics and extrinsic noise in hand it is possible, in principle, to locate the causes of cell-to-cell variability and quantify their contributions to system dynamics. Here, we develop a statistical framework for just this purpose, and we apply it to measurements obtained by quantitative image cytometry (Ozaki et al., 2010): data are obtained at discrete time points but encompass thousands of cells, which allows one to investigate the causes of cell-to-cell variability (Johnston, 2014). The in silico statistical model selection framework also has the advantage that it can be applied in situations where, e.g., dual reporter assays, which explicitly separate out extrinsic and intrinsic sources of variability (Hilfinger and Paulsson, 2011), cannot be applied.

**Figure 1:**
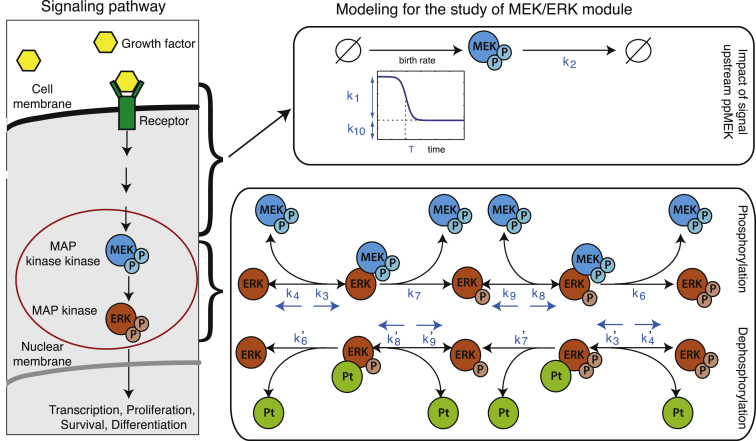
The MEK/ERK System and Modeling of the MEK/ERK Module The binding of a growth factor to its receptor activates a succession of reactions that lead to the phosphorylation of MEK; active MEK, in turn, phosphorylates ERK. In this study, we focus on the MEK/ERK module (circled in red). The impact of the stimulus and the upstream reactions on the evolution of the concentration of active MEK are modeled using a time-dependent function, which depends on three parameters (*k*_1_, *k*_10_, and *T*). In addition, active MEK is degraded with rate *k*_2_. The detailed mechanism of phosphorylation and dephosphorylation of ERK is represented in the bottom-right part of the figure. Pt denotes the cognate ERK phosphatase. The reaction rates are shown next to their associated reactions.

With this framework in hand we consider the dynamics of the central MEK-ERK core module of the MAPK signaling cascade, see Figure 1 (Santos et al., 2007, Inder et al., 2008). MAPK mediated signaling affects cell-fate decision-making processes (Eser et al., 2011)—including proliferation, differentiation, apoptosis, and cell stasis—and cell motility, and the mechanisms of MAPK cascades and their role in cellular information processing have been investigated extensively (Kiel and Serrano, 2009, Mody et al., 2009, Sturm et al., 2010, Takahashi et al., 2010, Aoki et al., 2011, Piala et al., 2014, Voliotis et al., 2014). Here, we take an engineering perspective and aim to characterize how MEK and ERK transmit signals. The upstream sources of noise in signaling involving MAPK cascades have been amply documented (see, e.g., Schoeberl et al., 2002, Santos et al., 2012, Sasagawa et al., 2005), as have their downstream consequences, e.g., in the context of stem cell-fate decision making (Miyanari and Torres-Padilla, 2012, Schröter et al., 2015). The manner in which MEK and ERK modulate this variability is less well understood in detail. Our aim is to answer three related questions: (1) are the dynamics of the MEK-ERK module noisy; (2) where might this noise originate; and (3) how does noise in the MEK-ERK system affect the ability of this important molecular system to relay information reliably?

Below we will first quantify the levels of cell-to-cell variability sources of noise in the system, with a special focus on the dynamics of active, i.e., phosphorylated, MEK and ERK; after this we will identify the sources of such noise and compare their relative contributions to cell-to-cell variability. We will show that our analysis is robust to both qualitative as well as quantitative changes in the upstream stimulation. With this in hand, we can then turn to an investigation of the effects cell-to-cell variability has on the ability of cell populations to respond to fluctuating signals.

## Results

### Quantifying Temporal Evolution of Cell-to-Cell Variability

**Figure 2:**
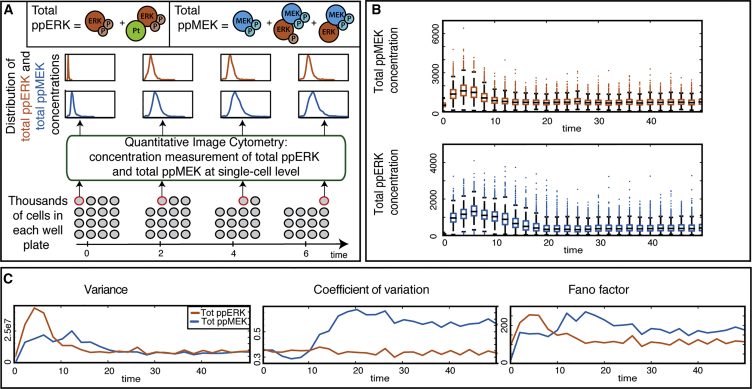
Measurement of the Evolution of the Joint Protein Distributions of Phosphorylated MEK and Phosphorylated ERK (A) Cells are plated in a medium and stimulated with NGF at *t* = 0. Every 2 min, thousands of cells are stimulated and the amount of total phosphorylated MEK and ERK (i.e., the sum of free and complex-bound forms) is measured at single-cell level using quantitative image cytometry, providing a series of cross-sectional snapshots of the joint distributions of the level of phosphorylated MEK and ERK. (B) Boxplots showing the distributions of the measured protein concentrations at each time point (the edges of the colored boxes are the 0.25 and 0.75 quantiles; the central mark is the median). (C) The temporal evolution of the variance, the coefficient of variation and the Fano factor for the distributions of the total amount of phosphorylated MEK and ERK.

We investigate the causes of cellular heterogeneity in vivo during ERK activation by phosphorylated MEK in PC12 cells. This cell-to-cell variability study is based on measurements of the concentration of phosphorylated MEK and ERK at the single-cell level obtained by quantitative image cytometry. Cells are plated in medium containing a fixed amount of neuronal growth factor (NGF) as the stimulus at time t = 0. Every 2 min, cells in one well are stimulated in order to quantify the concentration of the two proteins of interest providing us with a series of cross-sectional snapshots of the joint protein distributions of the total amount of phosphorylated MEK and ERK, see Figure 2A.

The observed distributions of the total amount of phosphorylated MEK and ERK are illustrated in Figures 2B and S1, and Figure 2C shows the evolution of the variance, the coefficient of variation and the Fano factor over time for both proteins. The variance over the cell population of the concentration is of the order of 10^5^ and significantly varies with time. We have examined experimental noise versus cell-to-cell variability of total ERKs in unstimulated PC12 cells (Figure S2) (Uda et al., 2013) and found that the experimental noise is negligible. In addition, cell size, cell volume, and Hoechst level (the dye used to quantify nucleic acid levels) make only negligible contributions to observed levels of cell-to-cell variability (Figure S3). We can thus rule out cell cycle, etc. as explanations for or cause of the temporal variability in the amount of active ERK.

### Statistical Investigation of the Cell-to-Cell Variability in the Core MEK-ERK Module

The analysis of the origins of cell-to-cell variability in the core MEK-ERK module (i.e., the MEK-ERK interactions as indicated by the red circle in Figure 1, left panel) requires us to determine the modes of ERK phosphorylation and dephosphorylation. ERK activation involves phosphorylation at both its tyrosine and threonine phosphorylation sites by its cognate kinase MEK (Ferrell and Bhatt, 1997, Ferrell and Ha, 2014). Previous studies (Toni et al., 2012) have shown that in vivo phosphorylation (as well as dephosphorylation) occurs in two steps where the kinase binds to the protein twice in order to phosphorylate the two sites successively (see Figure 1, bottom right). Using a Bayesian model selection approach, we confirm that this distributive mechanism best captures the observed average behavior in our data (see Figure S2). We therefore base our analysis of the origins of cell-to-cell variability on this mechanistic model with 20 model parameters including 12 reaction rates, four parameters describing the impact of the NGF stimulus and upstream signals and four parameters controlling the initial concentrations of the species involved in the MEK-ERK core system (see Figure 1 and Supplemental Experimental Procedures).

In this model of the MEK-ERK core module, we assume that the total abundance of ERK remains constant over the length of the experiment and is described by one of the model parameters. Previously, we had shown experimentally that total abundance of ERK does not change, while the levels of phosphorylation change considerably (Ozaki et al., 2010); therefore, it is indeed appropriate to model the cell-to-cell variability of total ERK as (extrinsic) parameter variability.

**Figure 3:**
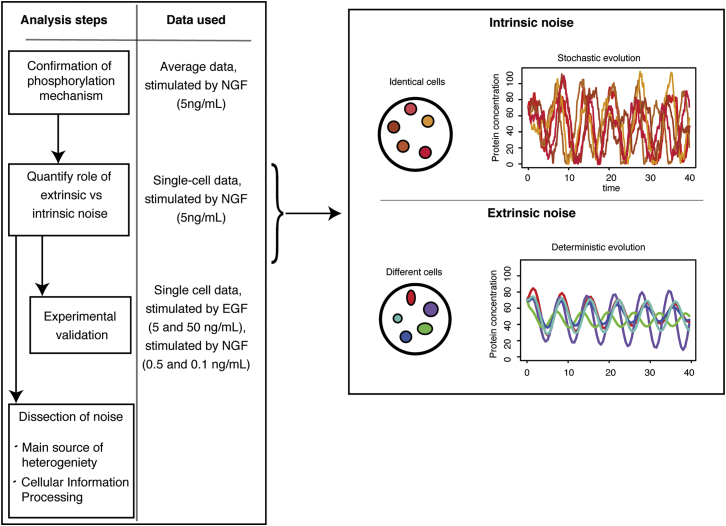
Elucidating the Origin of Cell-to-Cell Variability (Left) Flowchart of the analysis of origin of cell-to-cell variability as performed in this paper, highlighting which data are used at each steps. (Right) Within-cell variability can be caused by intrinsic noise, resulting from the stochastic nature of biochemical reactions, or extrinsic noise, arising from inherent differences between the cells.

The workflow adopted in this analysis is summarized in Figure 3. Given the mechanistic model of ERK phosphorylation described above, we will start by quantifying the relative contribution of intrinsic noise and extrinsic noise in the MEK-ERK core module. As illustrated in Figure 3, intrinsic noise results from the stochastic nature of biochemical reactions, while extrinsic noise arises from inherent differences between the cells. We will then experimentally validate our model of cell-to-cell variability by considering the response of the MEK-ERK system to different stimuli, and we will finish with a detailed analysis of the main source of cellular heterogeneity in the MEK-ERK core and the overall impact on MAPK-mediated cellular information processing.

### Relative Contribution of Extrinsic and Intrinsic Noise in the MEK-ERK Core Module

While it is straightforward to model extrinsic and intrinsic noise, quantifying their relative contributions to real molecular systems has thus far only been possible for systems where two-reporter assays are available (Elowitz et al., 2002, Swain et al., 2002). Here, we develop a statistical framework that allows us to obtain quantitative insights into the roles of these two sources of noise for signaling systems where direct measurements are typically not possible.

Intrinsic variability between cells arises from the stochastic nature of biochemical reactions. Each reaction occurs at a random time, so, even if the molecular species concentrations are identical in every cell at the beginning of the experiment, their evolution will inevitably vary from one cell to another. This intrinsic variability has traditionally been modeled using stochastic simulation algorithms such as the Gillespie algorithm. Here, we aim to examine whether there exists parameter sets for which the stochasticity of the biochemical reactions induces a similar variability between cells to that observed in the experimental data. In order to infer such parameter sets, we use the linear noise approximation (LNA) (Elf and Ehrenberg, 2003, Ferm et al., 2008), which provides an explicit Gaussian likelihood for stochastic biochemical reactions (see Experimental Procedures).

Extrinsic sources of variability stem from all those elements of the “real system” that are not explicitly modeled; these typically include factors such as inherent differences between the cells in terms of protein concentrations at the start of the experiment, and other biophysical parameters. To capture such effects, we allow model parameters to differ between cells (Shahrezaei et al., 2008, Toni and Tidor, 2013): the parameters for each cell are drawn from a log-normal distribution (with means and variances that will be inferred from the data). The potential sources of extrinsic noise in the MEK-ERK system are differences in the reaction rates between cells in the (de-)phosphorylation process of ERK, different initial concentrations of ERK and MEK, and differences in the upstream signaling cascades feeding into the MEK dynamics. The log-normal distribution has two advantages: it allows only positive values for reaction rates, and it allows parameters to vary over orders of magnitude if indicated by the data.

**Figure 4:**
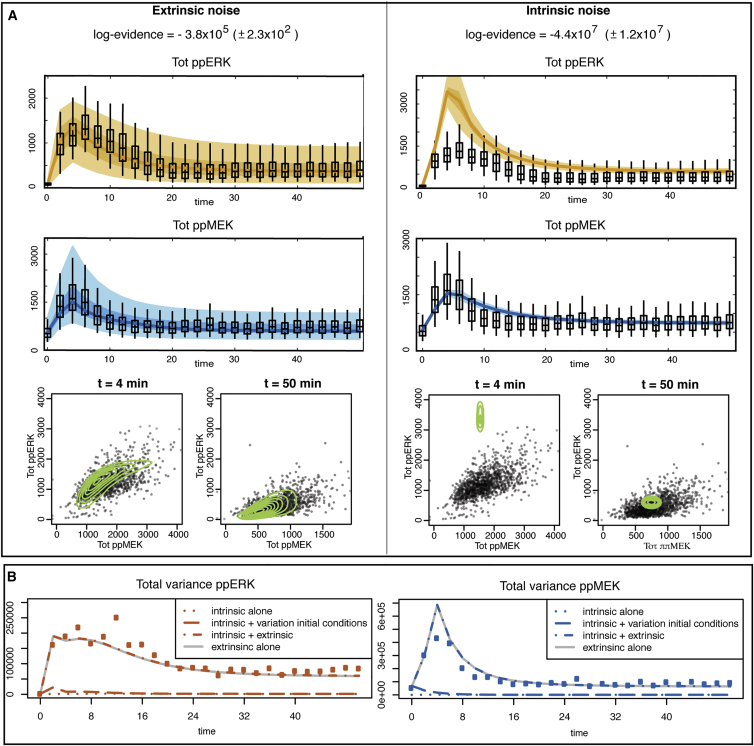
Intrinsic Noise Alone Cannot Explain the Observed Variability between Cells (A) In the top part of the figure, the evolution of the inferred marginal distributions over the cell population for the two noise models are displayed and compared to the single-cell data distributions (boxplots). The lines represent the median of the distributions, while the shaded regions indicate the regions delimited by 5^th^ and 95^th^ percentiles (lighter regions) and the one delimited by 25^th^ and 75^th^ percentiles (darker regions). The medians and percentiles shown here are the average of the medians and percentiles computed for 1,000 sets of parameters sampled from the posterior distribution. The logarithm of the evidence is shown for both noise models; these are strongly supportive of the extrinsic noise models. In the bottom part of the figure, the inferred joint distributions of the total amount of phosphorylated MEK and ERK over the cell population (contour green line) for the two noise models are displayed and compared to the single-cell data distributions (gray dots) for two time points (4 and 50 min). (B) Temporal evolution of the predicted variances over the cell population for the intrinsic noise model alone (dotted lines), the intrinsic noise model combined with a variation in initial conditions between cells (dashed lines), the intrinsic noise together with extrinsic noise (dash-dot lines), and the extrinsic noise alone (gray continuous line) show that the contribution of intrinsic noise is negligible. The squared dots represent the measured variance of the concentration of the two proteins over the cell populations.

Using the Bayesian framework developed in the Experimental Procedures, we analyze the roles of intrinsic and extrinsic noise in the single-cell data. The resulting statistical model evidence indicates that the extrinsic noise best explains the data. The evolution of the obtained distributions for MEK and ERK are shown and compared to the data in Figure 4A: only the extrinsic noise model can explain the observed high levels of cell-to-cell variability.

Variation in initial conditions is also not sufficient to generate the observed cell-to-cell variability; this is easily seen by sampling different values for the initial concentration of the species involved in the MEK-ERK system according to a log-normal distribution with mean and variance (given by the inferred means and variances in the extrinsic noise case) and simulating the model with intrinsic noise for each of these initial conditions. The total variance, which is the sum of (1) the mean over the different initial conditions of the variance due to the intrinsic noise, and (2) the variance over the different initial conditions of the mean over the intrinsic variability, is shown in Figure 4B. This shows that the variance including variation in initial conditions does not differ appreciably from the variance of intrinsic noise alone.

In a biological system, we expect extrinsic and intrinsic sources of noise: the cells are likely to be different in terms of initial molecular concentrations and the biochemical reactions occur at random times. We therefore compare the variances of the observed molecular species under extrinsic noise alone with the total variances under both extrinsic and intrinsic noise. From Figure 4B, it is apparent that the contribution of intrinsic noise to the total variation is negligible.

**Figure 5:**
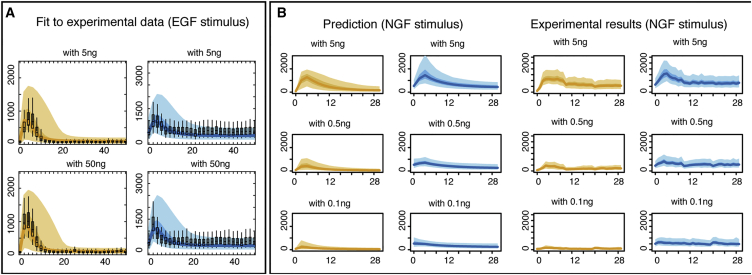
Prediction of the Impact of Growth Factor on Cell-to-Cell Variability (A) Evolution of the inferred distributions of the total amount of phosphorylated ERK and MEK in response to stimulation by EGF with two levels of intensity. The hyper-parameters for the initial conditions and the reaction rates are fixed to the previously estimated values (using the single-cell data in response to NGF stimulus), whereas the hyper-parameters describing the impact of the stimulus and upstream signals on the kinase are inferred here separately. The single-cell data distributions (boxplots) are compared to the inferred distributions (the lines represent the median of the distributions, while the shaded regions indicate the regions delimited by 5^th^ and 95^th^ percentiles for the lighter regions and the one delimited by 25^th^ and 75^th^ percentiles for the darker regions). (B) The predictions for the behavior of the total amount of phosphorylated ERK and MEK under the extrinsic noise model (left columns) are compared to experimental measurements (right columns) for different level of NGF intensity. The solid lines indicate the median value, while the shaded regions indicate the regions delimited by 5^th^ and 95^th^ percentiles (lighter zones) and by 25^th^ and 75^th^ percentiles (darker zones).

In order to validate the model further, we consider the response of the MEK-ERK system to different stimuli; while the upstream dynamics will be different (different receptors and different upstream intermediates as well as dependence on stimulus strength and temporal pattern [Fujita et al., 2010, Toyoshima et al., 2012]), the core MEK-ERK model, if parameterized correctly, should capture the dynamics. Here, we therefore use the hyper-parameters inferred previously except for those that correspond to the upstream dynamics, which we inferred directly from the EGF and NGF time courses. We find that extrinsic noise model explains the response of the MEK-ERK system to stimulation by EGF (Figure 5A) and different NGF stimulus intensities (Figures 5B and S4). The model with extrinsic noise shows good qualitative and quantitative agreement between model predictions and the new data obtained for different stimulus. Thus, our extrinsic noise model is capable of predicting the response of the core MEK-ERK module to other stimuli than those used in the model development. EGF and NGF are known to give rise to very different downstream behavior (Santos et al., 2007), but the modular nature of MAPK signaling (Mody et al., 2009) means that the characterization of the MEK-ERK component for one input (a given concentration of NGF) already yields a model that can also explain the response to other stimuli.

### Fluctuations in the Upstream Reactions and in the Degradation Rate of the Kinase Explain Most of the Cell-to-Cell Variability

**Figure 6:**
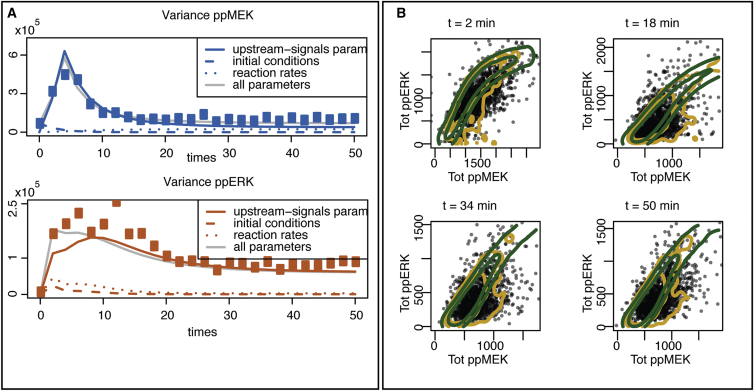
Factors Contributing to Cell-to-Cell Variability (A) Evolution of the predicted variances when only some of the model parameters vary from one cell to another: either the parameters that describe the effect of the upstream signals (continuous lines), the parameters controlling the initial conditions (dashed lines), or the reaction rates (dotted lines). The light gray continuous line is the predicted variance when all model parameters differ between cells. The squared dots represent the measured variance of the concentration of the two proteins over the cell populations. (B) Comparison of the observed (black dots) and the simulated joint distribution of the concentration of ppERK and ppMEK at different time points. The lines indicate the contour of the joint distribution when the system is simulated under the full extrinsic noise model (yellow) or only varying the parameters *k*_1_ and *k*_10_ between cells (green).

Our Bayesian analysis allows us to assess directly which parameters differ most between cells. For each parameter, we have estimates of the coefficient of variation across cells, and the parameters that contribute most to the observed cell-to-cell variability are those for which the inferred coefficient of variation is consistently and significantly different from zero (see Figure S6). We find five strongly contributing factors: three model parameters (*k*_1_, *k*_2_, and *k*_10_) and the two initial conditions that describe the level of background activity present in the cell at the point of stimulation. The degradation rate of active MEK (*k*_2_) affects the steady-state levels of cell-to-cell variability; the role of degradation reactions in determining levels of noise (and thus cell-to-cell variability) has been previously studied (Komorowski et al., 2013). The pulse height, *k*_1_, and the background upstream signal, *k*_10_, jointly characterize the impact of the NGF stimulus and the upstream reactions on the evolution of active MEK (see Figure 1, top right). The origins of noise upstream of MEK are well documented and therefore expected; here, our focus is on how MEK-ERK core dynamics modulate such variability. In Figure 6A, we illustrate the predominant role that these three model parameters that describe the effect of the upstream signal (*k*_1_, *k*_2_, and *k*_10_) have on the extent of cell-to-cell variability in this system.

To further investigate the role of the noise upstream to MEK compared to the noise in the core MEK-ERK module, we compare the joint distribution of the total amount of phosphorylated MEK and ERK when the system is simulated under the full extrinsic noise model or only varying the “driving” parameters *k*_1_ and *k*_10_ between cells (see Figure 6B). Simply varying the “driving” parameters can explain the evolution of the variance and correlation between the two proteins; the joint distribution of active MEK and ERK is only slightly better captured when we consider all the factors in the full extrinsic noise model.

### Impact of Cell-to-Cell Variability on Cellular Information Processing

**Figure 7:**
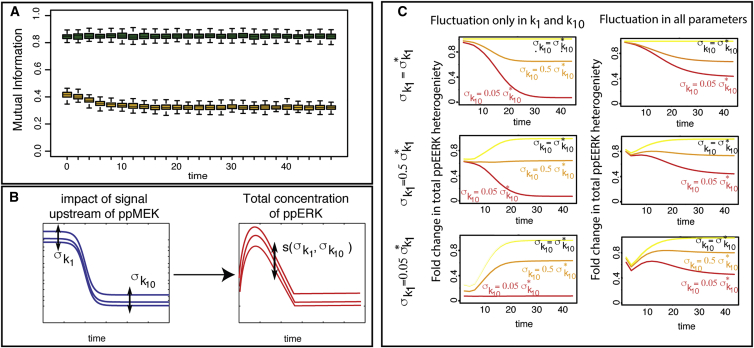
Information Processing of the MEK/ERK Module (A) Mutual information between ppMEK and ppERK when the system is simulated under the full extrinsic noise model (yellow) or only varying the parameters *k*_1_ and *k*_10_ between cells (green). (B) The variability in the impact of reactions upstream ppMEK is summarized by the variances $\sigma_{k_{1}}^{2}$ and $\sigma_{k_{10}}^{2}$. The level of cell-to-cell variability in the total concentration of ppERK at time *t* depends on $\sigma_{k_{1}}^{2}$ and $\sigma_{k_{10}}^{2}$ and is denoted by $\;s\left(\sigma_{k_{1}},\sigma_{k_{10}},t\right)$. (C) Illustration of the behavior of $\lambda \left(\sigma_{k_{1}},\sigma_{k_{10}},t\right)$ for decreasing values of $\sigma_{k_{1}}$ and $\sigma_{k_{10}}$—the red corresponding to the smallest and the yellow to the maximum SD. This subfigure is divided in two parts: in the left column, only parameters *k*_1_ and *k*_10_ vary between cells (all other model parameters are fixed to their mean value), whereas in the right column the full extrinsic noise model is considered where all model parameters differ between cells. Each panel corresponds to a fixed value of $\sigma_{k_{1}}$, while each line corresponds to a fixed value of $\sigma_{k_{10}}$.

We conclude our analysis by investigating the role that noise plays in mediating the response of the MEK-ERK module to external stimuli. We compute the mutual information between the total amount of phosphorylated MEK and ERK at different time points, simulating the system under extrinsic noise or varying only the parameters that seems to be related to most of the cellular variability (*k*_1_, *k*_2_, and *k*_10_)—all other model parameters are fixed to the inferred posterior mean values. We observe in Figure 7A that the presence of extrinsic noise decreases the level of transfer of information between the two species of interest. Thus, in a heterogeneous population of cells the statistical dependence between active MEK and ERK or, in other words, the expected information flowing through the MEK-ERK module is decreased. In light of the modest effect that within-module extrinsic noise appears to have on overall patterns of cell-to-cell variability in Figure 6, this profound change to the information transmission reliability might seem surprising. But it does reflect the complex behavior of the mutual information that can result from the interplay between system dynamics and extrinsic noise, and which has been demonstrated theoretically elsewhere (Mc Mahon et al., 2015). In other words, variability in the upstream signals is faithfully transmitted (active ERK is approximately proportional to active MEK) if we ignore variability in the other factors. But when this is taken into account, this relationship becomes less well defined and at the population level the flow of information through MEK-ERK is radically decreased.

To follow on from this, we analyze the level of cell-to-cell variability in the system’s output (i.e., the total amount of phosphorylated ERK) as a function of how variable the inputs (captured by the transient and sustained upstream intensities, *k*_1_ and *k*_10_, and their respective variances over the cell population $\sigma_{k_{1}}^{2}$ and $\sigma_{k_{10}}^{2}$) are. We simulate system output for given values of $\sigma_{k_{1}}^{2}$ and $\sigma_{k_{10}}^{2}$ and compute the ratio

$$\lambda \left(\sigma_{k_{1}},\sigma_{k_{10}},t\right)=\frac{s\left(\sigma_{k_{1}},\sigma_{k_{10}},t\right)}{s\left(\sigma_{k_{1}}^{*},\sigma_{k_{10}}^{*},t\right)}\;,$$

where $s\left(\sigma_{k_{1}},\sigma_{k_{10}},t\right)$ is the SD of the output at time *t* (see Figure 7B). Note that $\sigma_{k_{1}}^{*}=\sqrt{\mu_{k_{1}}}$ and $\sigma_{k_{10}}^{*}=\sqrt{\mu_{k_{10}}}$ are the maximum values of these SD, where $\mu_{k_{1}}$ and $\mu_{k_{10}}$ are the means over the cell population for, respectively, *k*_1_ and *k*_10_. The ratio $\lambda \left(\sigma_{k_{1}},\sigma_{k_{10}},t\right)$ quantifies the change in the level of cell-to-cell variability in the system’s output as the noise level in the input is decreased.

In the first instance, we assume that only the input signal strengths (*k*_1_ and *k*_10_) vary between cells. The evolution of $\lambda \left(\sigma_{k_{1}},\sigma_{k_{10}},t\right)$ over time when varying the variances $\sigma_{k_{1}}^{2}$ and $\sigma_{k_{10}}^{2}$ is shown in Figure 7C (left column). Before *t* = 8 min, $\lambda \left(\sigma_{k_{1}},\sigma_{k_{10}},t\right)$ increases with $\sigma_{k_{1}}^{2}$, whereas $\sigma_{k_{10}}^{2}$ has no impact on $\lambda \left(\sigma_{k_{1}},\sigma_{k_{10}},t\right)$. Conversely, after *t* = 24 min, $\lambda \left(\sigma_{k_{1}},\sigma_{k_{10}},t\right)$ increases with $\sigma_{k_{10}}^{2}$ but $\sigma_{k_{1}}^{2}$ no longer affects output variability. Thus variability in active ERK abundance across the cell population is initially strongly influenced by the variability in pulse height, *k*_1_, and subsequently by the variability in the sustained or background signal, *k*_10_.

To investigate further the effect of the variability in all model parameters on cellular information processing, we also simulate the system under extrinsic noise (varying all model parameters between cells) and compute once more $\lambda \left(\sigma_{k_{1}},\sigma_{k_{10}},t\right)$ for different signal variabilities. It is apparent from Figure 7C (right column) that, under the extrinsic noise model, the level of cell-to-cell variability in the system’s output remains substantially high even when the variability in the system’s input has been decreased considerably (λ ≃ 0.45 when $\sigma_{k_{1}}$ and $\sigma_{k_{10}}$ are divided by 20). Again the presence of extrinsic noise weakens the efficiency of signal transduction.

## Discussion

In this study, we have used quantitative image cytometry to elucidate the causes of population heterogeneity in the MAPK signaling cascade and presented a comprehensive analysis of cell-to-cell variability in the activation dynamics of the MEK-ERK system to environmental stimuli. With a reliable model for the (de-)phosphorylation mechanisms in hand, we were able to dissect the nature of the cell-to-cell variability inherent in the data. Recent models for ERK phosphorylation proposed in the literature (Ortega et al., 2006, Sturm et al., 2010, Harrington et al., 2013, Ferrell and Ha, 2014, Voliotis et al., 2014) allow for very rich dynamics, and a priori it is therefore impossible to make an appeal to the large number of MEK, ERK, and other molecules present in the eukaryotic cell, in order to rule out a role for intrinsic noise. The statistical framework developed for this study, however, gives a clear verdict in favor of extrinsic noise as the dominant factor for the observed cell-to-cell variability in the MEK-ERK system.

With the primary role of extrinsic noise established, we analyzed the contributions to cell-to-cell variability that arise from the different extrinsic sources of noise. Upstream of MEK many potential sources of noise and cell-to-cell variability have been identified in the literature, and our framework captures their contribution. In this study, the focus has been on the contributions to noise that arise from within the MEK-ERK module—this should be taken as akin to establishing the noise characteristics of, e.g., a transistor in electronics. We do find that there is considerable extrinsic variability in the module dynamics. However, the overall contribution of these within-module extrinsic sources of noise to the total dynamics quantitatively studied here are smaller than the upstream sources’ contributions.

We then investigated how this extrinsic noise interferes with signal transduction. Our analysis shows that the overall joint distribution of phosphorylated MEK and ERK can be understood largely in terms of the upstream noise. The full model that accounts for extrinsic variability both upstream and within-module can be argued to capture more of the nuances seen in the empirical data but otherwise does not differ very much. However, these two scenarios have profoundly different impact on the ability of the MEK-ERK module to transmit upstream information to the activity profile of phosphorylated ERK: without extrinsic noise in the core module, variable (including noisy) signals would be faithfully reproduced downstream. But the extrinsic variability in the module parameters distorts these signals and leads to a drastic reduction in the mutual information between incoming signal and ERK activity.

Our results can be interpreted in two ways: we may simply regard the MEK-ERK module as poorly engineered as its behavior depends on the cellular context, or we may view this as a bet hedging (Stumpf et al., 2002, Kussell and Leibler, 2005) strategy, which poises different cells to respond differentially to stimulation, thereby reducing the risk of an inappropriate population wide response to noisy signals. In development and tissue homeostasis (Rué and Martinez Arias, 2015) (and in regenerative medicine), it may be important to find ways to regulate population-level behavior; e.g., using inter- and intra-cellular feedback mechanisms that control cell-to-cell variability further (Michailovici et al., 2014).

The study presented here is based on experiments carried out in PC12 cell lines (Greene and Tischler, 1976), which, unlike in vitro setups, provide the cell physiological context. The activity of upstream and downstream processes affecting ERK may depend on cell type; this has, for example, been shown for nuclear shuttling, where even subtle differences between different cell lines can affect, e.g., the activity of nuclear ERK (Harrington et al., 2012). Our deliberate focus on the core MEK-ERK dynamics is less prone to such strong cell-type specificity over the timescales considered, whereas the potential of feedback from either ERK or any of its many downstream targets onto the MAPK cascade or proteins further upstream should be carefully considered in different cell types. The additional richness in behavior that such feedback (Ortega et al., 2006, Sturm et al., 2010) or explicit consideration of nuclear shuttling (Harrington et al., 2013) of ERK and MEK can induce warrants further investigation (Ozaki et al., 2010); here, over the time course considered, and in light of the data available such effects are marginal, but this may change as longer or more complex temporal stimulation patterns are considered. At the single-cell level, both feedback and shuttling are therefore clearly worth of further investigation.

It is important to keep in mind that no model will ever be able to contain all the constituent parts of any biological system of any real-world relevance. Therefore, extrinsic noise will always be an issue for modeling molecular and cellular systems. There are practical limitations to the current approach; notably it is not possible to fully describe correlations among the different extrinsic sources of noise (the number of parameters that we would have to estimate is simply too large); for example, interactions between kinases and phosphatases (Swain and Siggia, 2002) in MAPK may shape the response dynamics and such interactions are hard to capture in the extrinsic noise model. We believe, however, that the in silico approach developed here can serve to highlight such factors and may therefore be a guide to deciding which system aspects ought to be modeled explicitly. By pinpointing the sources of extrinsic noise, which are typically not obvious a priori, sound statistical modeling is able to provide deeper mechanistic insights and highlight where a model ought to be extended, or whether this is indeed necessary.

## Experimental Procedures

### Experimental Data Collecting Process

The concentrations of molecular species were measured using quantitative image cytometry (QIC) (Ozaki et al., 2010, Saito et al., 2013). PC12 cells were seeded at a density of 10^4^ cells per well in 96-well poly-L-lysine-coated glass-bottomed plates (Thermo Fisher Scientific). 24 hr after seeding, the medium was replaced with DMEM containing 25 mM HEPES and 0.1% of BSA. 18 hr after serum starvation, the stimulus is applied by replacing the starvation serum with a medium containing the stimulant (5 or 0.5 or 0.1 ng/ml). Our setup carries out stimulation in an incubator and achieved 1-min interval stimulation at 37°C under 5% CO_2_ in saturated air humidity. The cells are then fixed with 4% paraformaldehyde for 10 min and immunostained. Cells were subjected to QIC analysis with mouse anti-ppERK1/2 Sigma-Aldrich M8159 antibody and rabbit anti-pMEK1/2 Cell Signaling Technology 9121. Note that anti-pMEK antibody detects both singly (pS217 or pS221 alone) and doubly (pS217/221) phosphorylated MEK. Mouse monoclonal anti-ERK antibody (#4696, Cell Signaling Technology) and rabbit polyclonal anti-ERK antibody(#9102, Cell Signaling Technology) were used in Figure S2.

All images were analyzed with Cell Profiler (Kamentsky et al., 2011). The nuclear region was identified based on Hoechst imaging, and the cellular region was identified based on CellMask-stained images going out from the nuclear region. Total cellular signal intensity in nuclear regions and cellular regions were measured for ppERK and pMEK, respectively. We used these intensities as the concentrations of molecules. We used the cellular region in pixels as the cell size and the intensity of CellMask in the cellular region as a measure of cell volume.

The antibody against pMEK used detects both MEK1 and MEK2, all isoforms that phosphorylates ERK. In this study, we assume that the total phosphorylated MEK corresponds to doubly phosphorylated MEK. It is noted that no current technology is available to quantify the amounts of singly and doubly phosphorylated MEK. The antibody against ppERK detects both ERK1 and ERK2, all isoforms that are expressed in PC12 cells. To the best of our knowledge, there is no functional difference between the isoforms for both MEK and ERK at least in PC12 cells. Therefore, we did not make distinction between isoforms in this study.

### Parameter Inference and Model Evidence

We use a Bayesian approach in order to infer the parameters of the system (see Supplemental Experimental Procedures for a detailed list of the model parameters) and rank the candidate mechanistic models. Bayesian parameter inference is centered around the posterior probability distribution, $p\left(\theta |x^{*}\right)$, which strikes a compromise between prior knowledge, p(θ), about parameter vectors, θ, and the capacity of a parameter to explain the observed data, $x^{*}$, measured by the likelihood $p\left(x^{*}|\theta \right)$, via

$$p\left(\theta |x^{*}\right)=\frac{p\left(\theta \right)\;p\left(x^{*}|\theta \right)}{\int p\left(\tilde{\theta }\right)\;p\left(x^{*}|\tilde{\theta }\right)d\tilde{\theta }}\;.\;$$

Here, we evaluate the posterior using a sequential Monte Carlo (SMC) sampler proposed by Del Moral et al. (2006), which is easily parallelized. The output of the algorithm is a set of weighted parameter vectors ${\left\{\theta^{\left(i\right)},\omega^{\left(i\right)}\right\}}_{1\leq i\leq N}$. Here the parameter vector associated to the highest weight is called the *inferred parameter vector*. Technical details about our implementation of the SMC sampler algorithm are given in Supplemental Experimental Procedures.

The SMC sampler algorithm also enables us to evaluate the *model evidence* (Kirk et al., 2013), which is the probability to observe the data $x^{*}$ under the model *M* (given the alternative models considered),

$$p\left(x^{*}|M\right)=\int p\left(\theta |x^{*},M\right)p\left(\theta |M\right)d\theta \;.$$

The model evidence allows us to rank candidate models in terms of their ability to explain the observed data *x*^∗^: the best model is the one with the highest model evidence. In addition, the Bayes factor assesses the plausibility of two candidate models $M_{1}$ and $M_{2}$:

$$BF_{1,2}=\frac{p\left(x^{*}|M_{1}\right)}{p\left(x^{*}|M_{2}\right)}\;.\;$$

Whenever *BF*_1,2_ is larger than 30, the evidence in favor of model *M*1 is considered very strong (Jeffreys, 1961). We use our own implementation of the SMC sampler algorithm in Python as well as an interface to simulate the models in a computational efficient manner using a graphics processing unit (GPU) accelerated ordinary differential equation (ODE) solver (Zhou et al., 2011) and a C++ ODE solver for stiff models (Hindmarsh et al., 2005).

### Likelihood Functions

At each time point t∈T={0,2,4,…50}, the total amount of doubly phosphorylated MEK and ERK are measured in $N_{t}$ different cells. We denote by $x_{i,t}^{*}$ and $y_{i,t}^{*}$ the concentration of the two proteins in the *i*-th cell, $1\leq i\leq N_{t}$, and by ${\left\{{\bar{x}}_{t}^{*}\right\}}_{t\in \text{T}}$ and ${\left\{{\bar{y}}_{t}^{*}\right\}}_{t\in \text{T}}$ the observed average trajectories. In addition, we denote by $x_{t}\left(\theta \right)$ and $y_{t}\left(\theta \right)$ the solution of the system of ODE given the parameter vector θ at time *t*.

Assuming an independent Gaussian measurement error for each time point with constant variance *v*, the likelihood function for the average data measurements is

$$p\left({\left\{{\bar{x}}_{t}^{*},\;{\bar{y}}_{t}^{*}\right\}}_{t\in \text{T}}|\theta \right)=\prod_{t\in \text{T}}\phi \left({\bar{x}}_{t}^{*};x_{t}\left(\theta \right),v\right)\phi \left({\bar{y}}_{t}^{*};y_{t}\left(\theta \right),v\right),$$

where ϕ(.;m,v) is the probability density function of a normal distribution of mean *m* and variance *v*. The variance *v* is inferred simultaneously with the other parameters.

In order to derive the likelihood function in the intrinsic noise model efficiently (Golightly and Wilkinson, 2015), we use the linear noise approximation (LNA). The LNA provides a system of ODEs, which describes how the means and the variances of the molecular species vary over time. These equations are produced using the *StochSens* package (Komorowski et al., 2012). With $m_{t}^{x}\left(\theta \right)$, $m_{t}^{y}\left(\theta \right)$, $v_{t}^{x}\left(\theta \right)$, and $v_{t}^{y}\left(\theta \right)$ denoting the solutions of the ODEs describing the means and variances for the parameter θ at time *t*, the likelihood $p\left({\left\{x_{i,t}^{*},y_{i,t}^{*}\right\}}_{i,t}|\theta \right)$ is equal to

$$\prod_{t\in \text{T}}\prod_{i=1}^{N_{t}}\phi \left(x_{i,t}^{*};m_{t}^{x}\left(\theta \right),v_{t}^{x}\left(\theta \right)\right)\phi \left(y_{i,t}^{*};m_{t}^{y}\left(\theta \right),v_{t}^{y}\left(\theta \right)\right).$$

Extrinsic noise is modeled by considering that each cell has a different set of parameters. The distribution of each parameter across the cell population is assumed to be log-normal. We assume that these distributions are independent and denote by *μ*_*θ*_ and *σ*_*θ*_^2^ the vector of the means and variances of these distribution, respectively. Due to computational cost, we do not consider any correlation between the parameters. There is no closed-form expression for the probability $p\left({\left\{x_{i,t}^{*},y_{i,t}^{*}\right\}}_{i,t}|\mu_{\theta },\sigma_{\theta }^{2}\right)$, and we use the so-called Unscented Transform (UT) (Silk et al., 2011), which, given the first two moments $\mu_{\theta }$ and $\sigma_{\theta }^{2}$ of the distribution in the parameter space, provides an approximation of the evolution of the means and variances of the two species of interest.

We denote by $m_{t}^{x}\left(\mu_{\theta },\sigma_{\theta }^{2}\right)$ and $m_{t}^{y}\left(\mu_{\theta },\sigma_{\theta }^{2}\right)$ the resulting mean behaviors of the two species at time *t*, and by $v_{t}^{x}\left(\mu_{\theta },\sigma_{\theta }^{2}\right)$ and $v_{t}^{y}\left(\mu_{\theta },\sigma_{\theta }^{2}\right)$ the associated variances. Assuming that the concentration of the doubly phosphorylated MEK and ERK proteins are log-normally distributed, we obtain that the likelihood $\;p\left({\left\{x_{i,t}^{*},y_{i,t}^{*}\right\}}_{i,t}|\mu_{\theta },\sigma_{\theta }^{2}\right)$ is

$$\prod_{t\in \text{T}}\prod_{i=1}^{N_{t}}\psi \left(x_{i,t}^{*};m_{t}^{x}\left(\mu_{\theta },\sigma_{\theta }^{2}\right),v_{t}^{x}\left(\mu_{\theta },\sigma_{\theta }^{2}\right)\right)\psi \left(y_{i,t}^{*};m_{t}^{y}\left(\mu_{\theta },\sigma_{\theta }^{2}\right),v_{t}^{y}\left(\mu_{\theta },\sigma_{\theta }^{2}\right)\right).$$

Here ψ(.;m,v) is the probability density function of a log-normal distribution with mean *m* and variance *v*. The Supplemental Experimental Procedures contains additional technical details on the computation and the UT algorithm.

### Mutual Information

The mutual information between two species (ppERK and ppMEK) is computed based on measurements of the protein concentrations in single-cells at different time points (Mc Mahon et al., 2015). For each time point, we estimate the mutual information using a kernel density estimate of the joint distribution. We use a Gaussian kernel with a diagonal covariance matrix and marginal variances equal to 1.06*σN*^–1*/*5^ where σ is the marginal variance of the data and *N* is the number of data points (Silverman, 1986).

## Author Contributions

S.F., C.P.B., S.K., and M.P.H.S. designed the study; T.K., K.K., T.T., T.W., and S.K. performed the data collection and initial processing; S.F., C.P.B., P.D.W.K., and S.S.M. performed modeling and statistical analysis; S.F., C.P.B., P.D.W.K., S.K., and M.P.H.S. wrote the paper; all authors approved the final version of the manuscript.

## Supplemental Information

Document S1. Supplemental Experimental Procedures and Figures S1–S7

Document S2. Article plus Supplemental Information
