# Supplementary material for: Robustness of MEK-ERK Dynamics and Origins of Cell-to-Cell Variability in MAPK Signaling
Source: Cell Rep. 2016 Jun 2;15(11):2524–35. doi: 10.1016/j.celrep.2016.05.024 (PMC4914773; doi:10.1016/j.celrep.2016.05.024)
Supplement: Document S1. Supplemental Experimental Procedures and Figures S1–S7 [file mmc1.pdf]

Cell Reports, Volume *15*

Supplemental Information

Robustness of MEK-ERK Dynamics and Origins of Cell-to-Cell Variability in MAPK Signaling

Sarah Filippi, Chris P. Barnes, Paul D.W. Kirk, Takamasa Kudo, Katsuyuki Kunida, Siobhan S. McMahon, Takaho Tsuchiya, Takumi Wada, Shinya Kuroda, and Michael P.H. Stumpf

Supplemental Figures

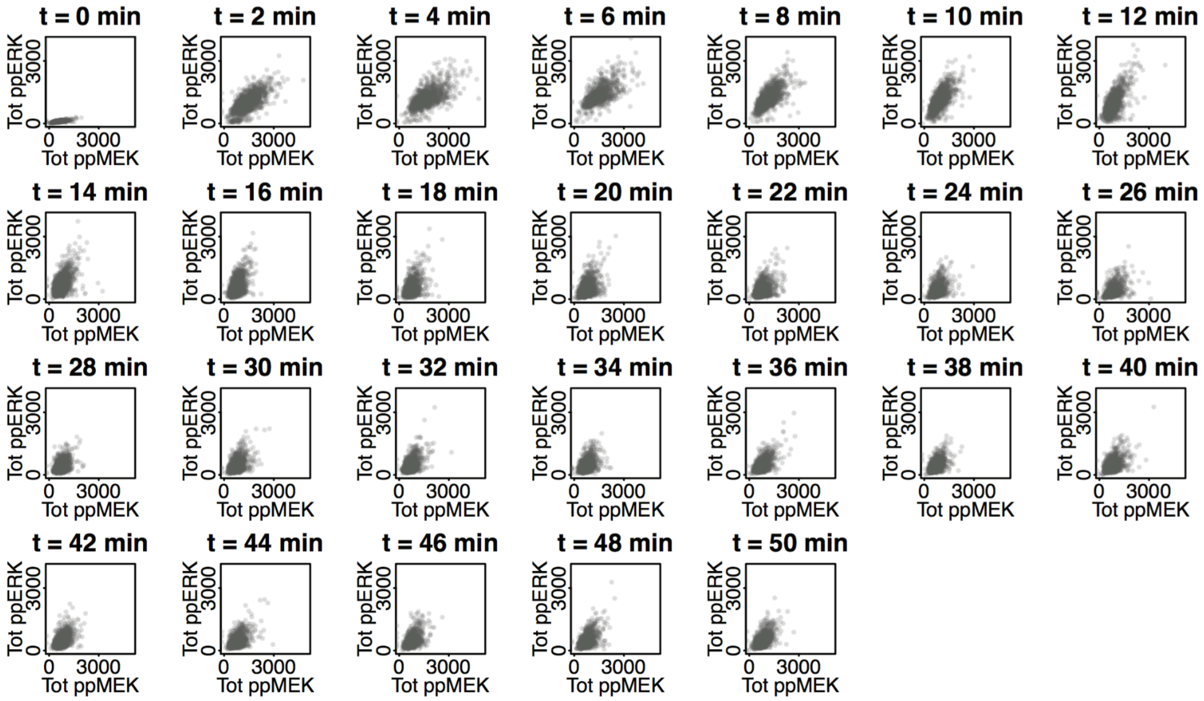

Figure S1: **Measurement of the evolution of the joint protein distributions of phosphorylated MEK and phosphorylated ERK. (Related to Figure 2B)** The joint distribution of total phosphorylated MEK and ERK every two minutes. Each dot corresponds to a single cell.

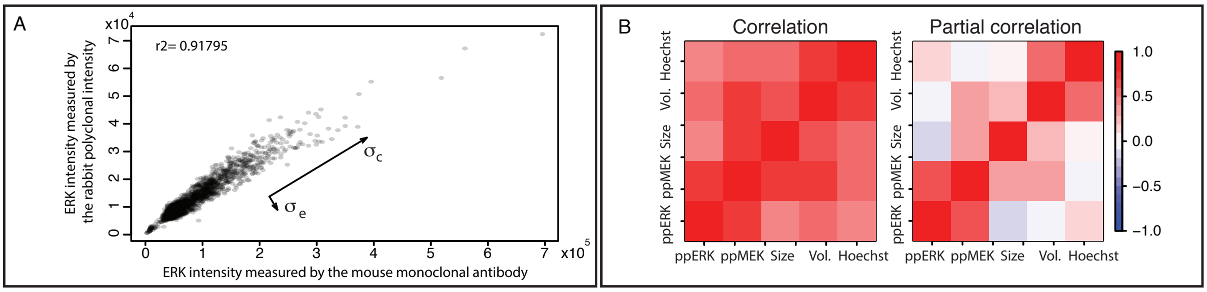

Figure S2: **Elucidating the origin of cell-to-cell variability. (Related to Figure 2) (A)** Estimation of experimental noise (Uda et al., 2013). Correlation of signal intensity of the total amount of ERKs measured by the two different antibodies. Each dot denotes the signal intensity of a single cell. We measured the total amount of ERKs by double-staining with mouse monoclonal and rabbit polyclonal antibodies. The correlation coefficient, r was 0.92. The cell-to-cell variation and experimental noise correspond to the variation along the major axis (σ_c) and that orthogonal to the major axis (σ_e), respectively. Copyright permission from Science. **(B)** In addition to the experimental measurements for the total amount of doubly phosphorylated ERK and MEK our assay also obtained measurements for cell-size, cell volume and Hoechst intensity in each cell. We computed the correlation and partial correlations between these 5 measurements using the R package GeneNet (Schäfer et al., 2001). Partial correlation is a much more powerful measure of statistical dependencies than correlation as has been discussed in detail by several authors (Kolaczyk, 2009; Schäfer and Strimmer, 2005; Thorne et al., 2013); it allows to measure dependences between each pairs of variables conditional on all other variables. Here, we compute the partial correlation between every pair of variables given the three other variables as controlling variables. For example, the partial correlation between the total amount of doubly phosphorylated ERK and MEK is the correlation between the residuals resulting from the linear regression of the total amount of doubly phosphorylated ERK and MEK respectively given the cell size, the cell volume and the Hoechst intensity. Both plots are on the same scale, see colour bar.

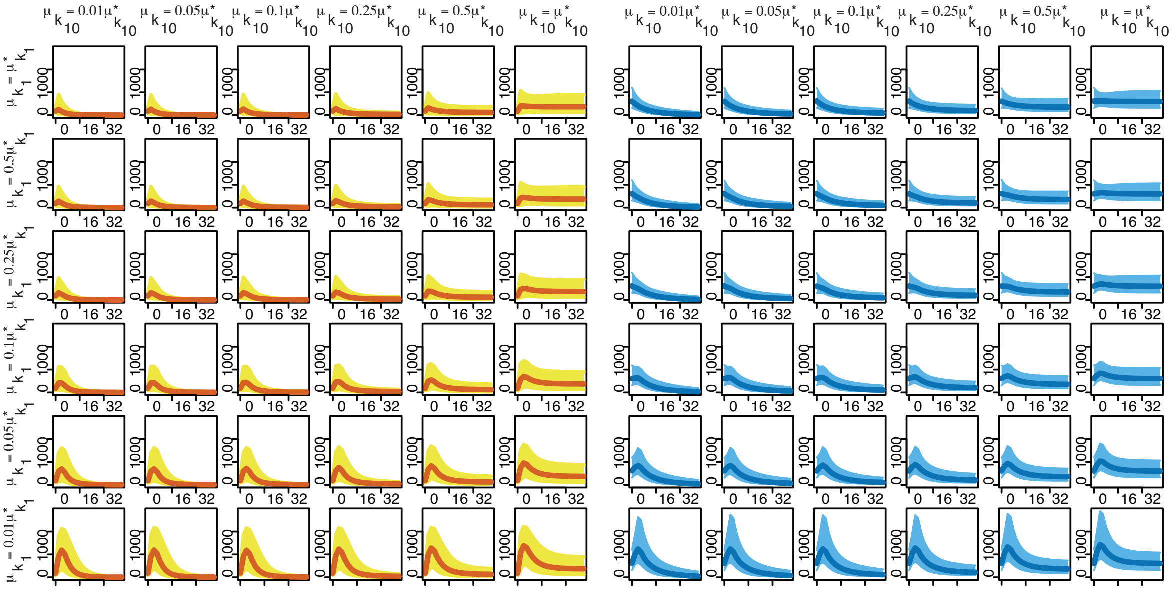

Figure S3: **Prediction of the impact of NGF intensity on cell-to-cell variability under the extrinsic noise model. (Related to Figure 5)** Predicted total amounts (medians and predicted 90% credible intervals) of doubly phosphorylated ERK (orange) and MEK (blue) under the extrinsic noise model are shown for decreasing values of the hyper-parameters μ_{k_1} and $\mu_{k_{10}}$. The reference hyper-parameters, $\mu^*_{k_1}$ and $\mu^*_{k_{10}}$, are the one inferred from the data.

Supplemental Experimental Procedure

Contents

S1 Description of the mechanistic models and the parameters **5**
S1.1 Systems of ordinary differential equations to model processive and distributive phosphorylation and dephosphorylation mechanisms . . . 5
S1.2 Model parameters . . . 9
S1.3 Observed species . . . 10
S1.4 Model selection: distributive phosphorylation and dephosphorylation best explains the average behaviour . . . 10

S2 Details on the implementation of the SMC sampler algorithm **12**

S3 The intrinsic noise model **13**
S3.1 Investigating the precision of the Linear Noise Approximation . . . 13
S3.2 Efficient sampling of the parameter space . . . 13

S4 The extrinsic noise model **15**
S4.1 Using Unscented Transform to derive a likelihood function for the extrinsic noise model . . . 15
S4.2 Extrinsic noise: identification of model parameters that significantly vary between cells . . . 16

S1 Description of the mechanistic models and the parameters

ERK activation requires phosphorylation at both its tyrosine and threonine phosphorylation sites by its cognate kinase MEK (Ferrell and Bhatt, 1997a), and two mechanisms for phosphorylation and dephosphorylation have been proposed (Ferrell and Bhatt, 1997b; Gunawardena, 2007; Toni et al., 2012), which are here referred to as processive and distributive. In the processive (P) mechanism, the kinase binds the protein and catalyzes the phosphorylation at both sites before dissociating from the doubly phosphorylated substrate protein. In the distributive (D) mechanism phosphorylation occurs in two steps where the kinase binds to the protein twice in order to phosphorylate the two site successively. Previous studies (Toni et al., 2012) have shown that *in vivo* phosphorylation (as well as dephosphorylation) occurs in a distributive way. In this section, we describe the four mechanistic models and confirm that this distributive mechanism best captures the observed average behavior in our data.

S1.1 Systems of ordinary differential equations to model processive and distributive phosphorylation and dephosphorylation mechanisms

In this section we derive the mathematical equations for different potential mechanistic models of the ERK (de-)phosphorylation process. In the following M denotes doubly phosphorylated MEK, which acts as a kinase, and E, pE and ppE denote un-, singly and doubly phosphorylated ERK, respectively; Pt represents the phosphotase activity. The phosphorylation and dephosphorylation processes of ERK involves these 5 species as well as the following protein complexes: $E \cdot M$, $pE \cdot M$, $ppE \cdot Pt$ and $pE \cdot Pt$.

The mechanistic models are described by the following reactions (see also Figure S4):

Phosphorylation

$$\text{Processive:}\ E + M \underset{k_4}{\overset{k_3}{\rightleftharpoons}} E \cdot M \xrightarrow{k_5} pE \cdot M \xrightarrow{k_6} ppE + M$$

$$\text{Distributive:}\ E + M \underset{k_4}{\overset{k_3}{\rightleftharpoons}} E \cdot M \xrightarrow{k_7} pE + M \underset{k_9}{\overset{k_8}{\rightleftharpoons}} pE \cdot M \xrightarrow{k_6} ppE + M$$

Dephosphorylation

$$\text{Processive:}\ ppE + Pt \underset{k_4'}{\overset{k_3'}{\rightleftharpoons}} ppE \cdot Pt \xrightarrow{k_5'} pE \cdot Pt \xrightarrow{k_6'} E + Pt$$

$$\text{Distributive:}\ ppE + Pt \underset{k_4'}{\overset{k_3'}{\rightleftharpoons}} ppE \cdot Pt \xrightarrow{k_7'} pE + Pt \underset{k_9'}{\overset{k_8'}{\rightleftharpoons}} pE \cdot Pt \xrightarrow{k_6'} E + Pt$$

The reaction rates are shown above or below the corresponding reactions.

In the derivation of the mechanistic models we incorporate the known experimental and biophysical constraints. First, since the concentration of active MEK depends on the upstream signals, its evolution is described by two additional reactions: a production reaction and a degradation reaction, which are given by

$$\emptyset \xrightarrow{g(k_1, T, k_{10}, t)} M$$
$$M \xrightarrow{k_2} \emptyset,$$

where g is given by

$$g(k_1, T, k_{10}, t) = k_{10} + \frac{k_1}{\exp(t - T) + 1}\ .$$

In addition, some of the binding and un-binding reactions are reversible, whereas the phosphorylation and dephosphorylation reactions are not. The last assumption concerns the phosphorylation of ERK at the second

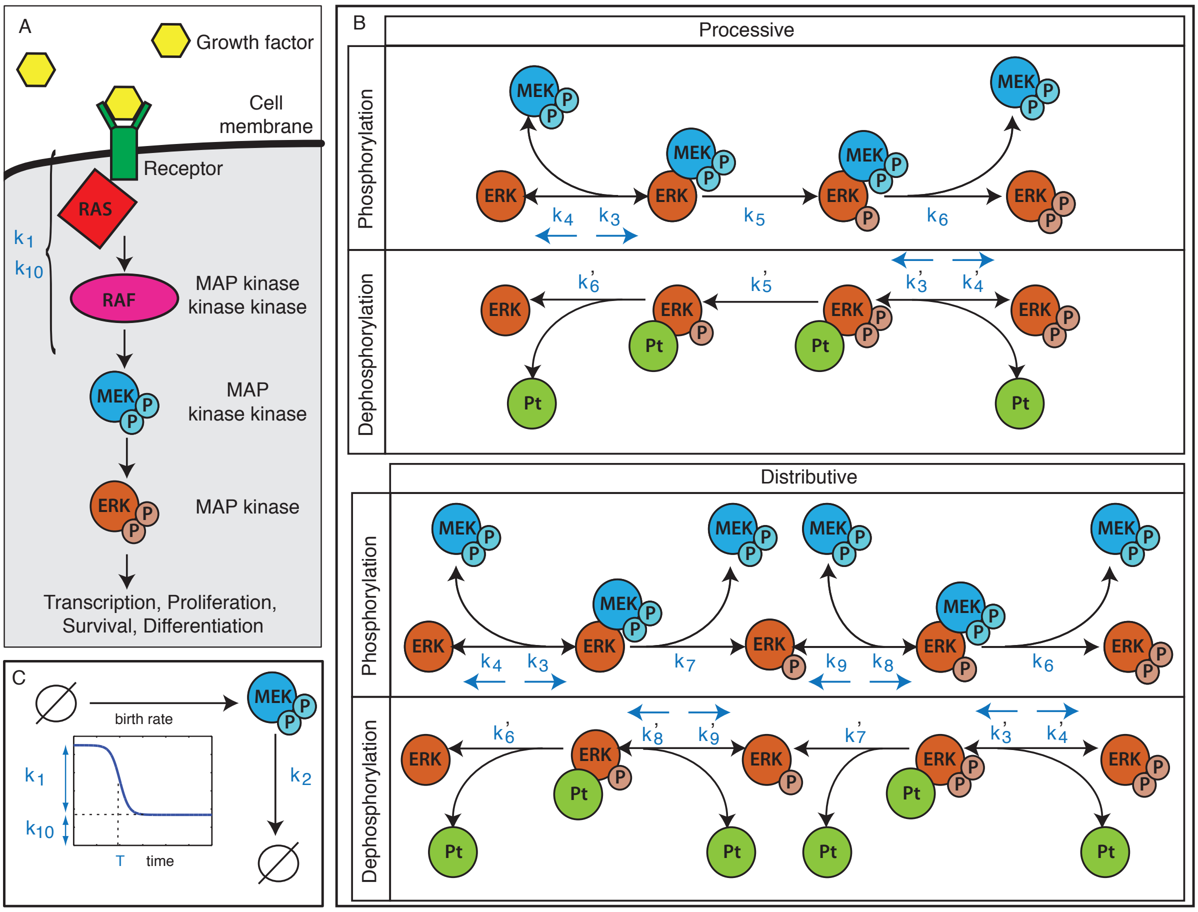

Figure S4: **MAPK Signalling. (Related to Figure 1) (A)** The RAS-RAF-ERK signal transduction cascade in response to a neural growth factor (NGF), which activates the membrane-bound GTPase (RAS); this leads to the activation of the RAF kinase and subsequently to the phosphorylation of MEK; active MEK in turn phosphorylates ERK. **(B)** Processive and distributive phosphorylation and dephosphorylation processes of ERK. Pt denotes the cognate ERK phosphatase. The reaction rates are shown next to their associated reactions. **(C)** The impact of the NGF stimulus and the upstream reactions on the evolution of the concentration of active MEK are modelled using a time dependent function which depends on three parameters: k_1 describes the pulse height, k_{10} the background signal and T the time at which the influence of the upstream reactions drops down. In addition active MEK is degraded with rate k_2.

site in the processive mechanism. We describe both this second phosphorylation and the unbinding of the double phosphorylated ERK to the active MEK in a single reaction. Moreover, since we work on time-scales where transcriptional responses can be ignored, we fix the total amounts (i.e. the sum of free and complex bound forms) of Pt, denoted by Pt_{tot} as well as the total amount of E, including all isophorms, denoted by E_{tot} (see Ozaki *et al.*, 2010).

By considering all possible combinations of processive (P) and distributive (D) phosphorylation and dephosphorylation reactions we can construct four possible models: DD, DP, PD and PP, where, for example, DP means distributive phosphorylation and processive dephosphorylation. The evolution of the concentrations (here denoted by $[A]_t$ for the concentration of species A at time t) of the 9 species is described by a set of ordinary differential equations.

Distributive phosphorylation - Distributive dephosphorylation (DD)

$$\begin{aligned}
\frac{d[M]_t}{dt} &= g(k_1, T, k_{10}, t) - k_2[M]_t - k_3[E]_t[M]_t + k_4[E.M]_t + k_6[pE.M]_t + k_7[E.M]_t \\
&\quad - k_8[pE]_t[M]_t + k_9[pE.M]_t \\
\frac{d[E]_t}{dt} &= -k_3[E]_t[M]_t + k_4[E.M]_t + k_6'[pE.Pt]_t \\
\frac{d[E.M]_t}{dt} &= k_3[E]_t[M]_t - k_4[E.M]_t - k_7[E.M]_t \\
\frac{d[pE]_t}{dt} &= k_7[E.M]_t - k_8[pE]_t[M]_t + k_9[pE.M]_t + k_7'[ppE.Pt]_t - k_8'[pE]_t[Pt]_t + k_9'[pE.Pt]_t \\
\frac{d[pE.M]_t}{dt} &= -k_6[pE.M]_t + k_8[pE]_t[M]_t - k_9[pE.M]_t \\
\frac{d[ppE]_t}{dt} &= k_6[pE.M]_t - k_3'[ppE]_t[Pt]_t + k_4'[ppE.Pt]_t \\
\frac{d[Pt]_t}{dt} &= -k_3'[ppE]_t[Pt]_t + k_4'[ppE.Pt]_t + k_6'[pE.Pt]_t + k_7'[ppE.Pt]_t - k_8'[pE]_t[Pt]_t + k_9'[pE.Pt]_t \\
\frac{d[ppE.Pt]_t}{dt} &= k_3'[ppE]_t[Pt]_t - k_4'[ppE.Pt]_t - k_7'[ppE.Pt]_t \\
\frac{d[pE.Pt]_t}{dt} &= -k_6'[pE.Pt]_t + k_8'[pE]_t[Pt]_t - k_9'[pE.Pt]_t
\end{aligned}$$

Distributive phosphorylation - Processive dephosphorylation (DP)

$$\frac{d[M]_t}{dt} = g(k_1, T, k_{10}, t) - k_2[M]_t - k_3[E]_t[M]_t + k_4[E.M]_t + k_6[pE.M]_t + k_7[E.M]_t - k_8[pE]_t[M]_t + k_9[pE.M]_t$$

$$\frac{d[E]_t}{dt} = -k_3[E]_t[M]_t + k_4[E.M]_t + k'_6[pE.Pt]_t$$

$$\frac{d[E.M]_t}{dt} = k_3[E]_t[M]_t - k_4[E.M]_t - k_7[E.M]_t$$

$$\frac{d[pE]_t}{dt} = k_7[E.M]_t - k_8[pE]_t[M]_t + k_9[pE.M]_t$$

$$\frac{d[pE.M]_t}{dt} = -k_6[pE.M]_t + k_8[pE]_t[M]_t - k_9[pE.M]_t$$

$$\frac{d[ppE]_t}{dt} = k_6[pE.M]_t - k'_3[ppE]_t[Pt]_t + k'_4[ppE.Pt]_t$$

$$\frac{d[Pt]_t}{dt} = -k'_3[ppE]_t[Pt]_t + k'_4[ppE.Pt]_t + k'_6[pE.Pt]_t$$

$$\frac{d[ppE.Pt]_t}{dt} = k'_3[ppE]_t[Pt]_t - k'_4[ppE.Pt]_t - k'_5[ppE.Pt]_t$$

$$\frac{d[pE.Pt]_t}{dt} = k'_5[ppE.Pt]_t - k'_6[pE.Pt]_t$$

Processive phosphorylation - Distributive dephosphorylation (PD)

$$\frac{d[M]_t}{dt} = g(k_1, T, k_{10}, t) - k_2[M]_t - k_3[E]_t[M]_t + k_4[E.M]_t + k_6[pE.M]_t$$

$$\frac{d[E]_t}{dt} = -k_3[E]_t[M]_t + k_4[E.M]_t + k'_6[pE.Pt]_t$$

$$\frac{d[E.M]_t}{dt} = k_3[E]_t[M]_t - k_4[E.M]_t - k_5[E.M]_t$$

$$\frac{d[pE]_t}{dt} = k'_7[ppE.Pt]_t - k'_8[pE]_t[Pt]_t + k'_9[pE.Pt]_t$$

$$\frac{d[pE.M]_t}{dt} = k_5[E.M]_t - k_6[pE.M]_t$$

$$\frac{d[ppE]_t}{dt} = k_6[pE.M]_t - k'_3[ppE]_t[Pt]_t + k'_4[ppE.Pt]_t$$

$$\frac{d[Pt]_t}{dt} = -k'_3[ppE]_t[Pt]_t + k'_4[ppE.Pt]_t + k'_6[pE.Pt]_t + k'_7[ppE.Pt]_t - k'_8[pE]_t[Pt]_t + k'_9[pE.Pt]_t$$

$$\frac{d[ppE.Pt]_t}{dt} = k'_3[ppE]_t[Pt]_t - k'_4[ppE.Pt]_t - k'_7[ppE.Pt]_t$$

$$\frac{d[pE.Pt]_t}{dt} = -k'_6[pE.Pt]_t + k'_8[pE]_t[Pt]_t - k'_9[pE.Pt]_t$$

Processive phosphorylation - Processive dephosphorylation (PP)

$$
\begin{aligned}
\frac{d[M]_t}{dt} &= g(k_1, T, k_{10}, t) - k_2[M]_t - k_3[E]_t[M]_t + k_4[E.M]_t + k_6[pE.M]_t \\
\frac{d[E]_t}{dt} &= -k_3[E]_t[M]_t + k_4[E.M]_t + k'_6[pE.Pt]_t \\
\frac{d[E.M]_t}{dt} &= k_3[E]_t[M]_t - k_4[E.M]_t - k_5[E.M]_t \\
\frac{d[pE.M]_t}{dt} &= k_5[E.M]_t - k_6[pE.M]_t \\
\frac{d[ppE]_t}{dt} &= k_6[pE.M]_t - k'_3[ppE]_t[Pt]_t + k'_4[ppE.Pt]_t \\
\frac{d[Pt]_t}{dt} &= -k'_3[ppE]_t[Pt]_t + k'_4[ppE.Pt]_t + k'_6[pE.Pt]_t \\
\frac{d[ppE.Pt]_t}{dt} &= k'_3[ppE]_t[Pt]_t - k'_4[ppE.Pt]_t - k'_5[ppE.Pt]_t \\
\frac{d[pE.Pt]_t}{dt} &= k'_5[ppE.Pt]_t - k'_6[pE.Pt]_t
\end{aligned}
$$

In order to simulate these ODE systems one needs to determine the initial conditions for the concentrations of the 9 molecular species. We assume that the initial concentrations of the complexes $E.M$, pE, $pE.M$, $ppE.Pt$ and $pE.Pt$ are equal to 0. In addition, the initial conditions of the species E and ppE are constrained so that $[E]_t+[ppE]_t = E_{tot}$ and the molecular concentration of ppE and M are determined by the observation. Therefore, if we denote by ppE_0 and M_0 the initial concentrations of the two observed molecular species, we have the following initial conditions:

$$
\begin{aligned}
[M]_0 &= M_0 \\
[E]_0 &= E_{tot} - ppE_0 \\
[ppE]_0 &= ppE_0 \\
[Pt]_0 &= Pt_{tot}
\end{aligned}
$$

S1.2 Model parameters

In the rest of this supplemental material and in the manuscript, the term ”model parameter” includes reaction rates, the 4 parameters describing the impact of upstream signals on active MEK (T, k_1, k_2 and k_{10}) and the 4 parameters related to the initial molecular concentration (M_0, ppE_0, E_{tot} and Pt_{tot}). In the following table, we summarise the number of parameters for each model.

Model	Reaction rates	Parameters related to upstream signals	Parameters related to initial concentrations	Total number of parameters
DD	12	4	4	20
DP	10	4	4	18
PD	10	4	4	18
PP	8	4	4	16

The model parameters are inferred using a Bayesian approach, therefore, a prior distribution over the parameter space need to be specified. We used uniform priors for each parameter based on the broader prior range proposed by Toni et al. (2012). Below we summarise the prior ranges used for each of the parameters (when performing parameter inference based on the average data, we set $M_0 = 636$ and $ppE_0 = 188$ according to the observed data).

Parameter	Lower limit	Upper limit	Parameter	Lower limit	Upper limit
k_1	0	100	k_{10}	1	2
T	200	400	k_2	2.10^{-3}	3.10^{-3}
k_3	0	1	k'_3	1	1000
k_4	0	1	k'_4	1	10^5
k_5	0	10^5	k'_5	0	10^5
k_6	100	1000	k'_6	0	100
k_7	1	500	k'_7	0	10
k_8	0	1	k'_8	0	1000
k_9	0	10	k'_9	0	10^5
E_{tot}	500	$1.8.10^4$	M_0	0	1000
Pt_{tot}	200	10^4	ppE_0	0	500

S1.3 Observed species

Quantitative image cytometry enables us to quantify the concentration of doubly phosphorylated ERK and MEK. More precisely, the total amount of free and complex bound forms of respectively doubly phosphorylated ERK and MEK are measured, i.e.

$$\text{total amount of doubly phosphorylated ERK} = ppE + ppE.Pt$$
$$\text{total amount of doubly phosphorylated MEK} = M + E.M + pE.M \ .$$

In the following and in the manuscript, we denote by

$$x_t = [ppE]_t + [ppE.Pt]_t$$

and

$$y_t = [M]_t + [E.M]_t + [pE.M]_t \ .$$

The quantities involved here are the solutions of the system of differential equations described above and typically depend on a vector of model parameter θ (see subsection S1.2).

S1.4 Model selection: distributive phosphorylation and dephosphorylation best explains the average behaviour

We use Bayesian parameter inference and model selection to determine which mechanism best captures the observed average behavior. Assuming an independent Gaussian measurement error for each time point with constant variance, we obtain the likelihood derived in *Experimental Procedure*. The best fits to the data for the four models are shown in Figure S5A. Although both the DD (distributive phosphorylation and dephosphorylation) and PD (processive phosphorylation and distributive dephosphorylation) models provide good fits to the data, the DD model receives much higher support by the Bayesian model ranking procedure (see Figure S5B). We will therefore base our analysis of the origins of cell-to-cell variability on this DD model with 20 model parameters including 12 reaction rates, 4 parameters describing the impact of the NGF stimulus and upstream signals and 4 parameters controlling the initial concentrations of the species involved in the ERK-MEK system.

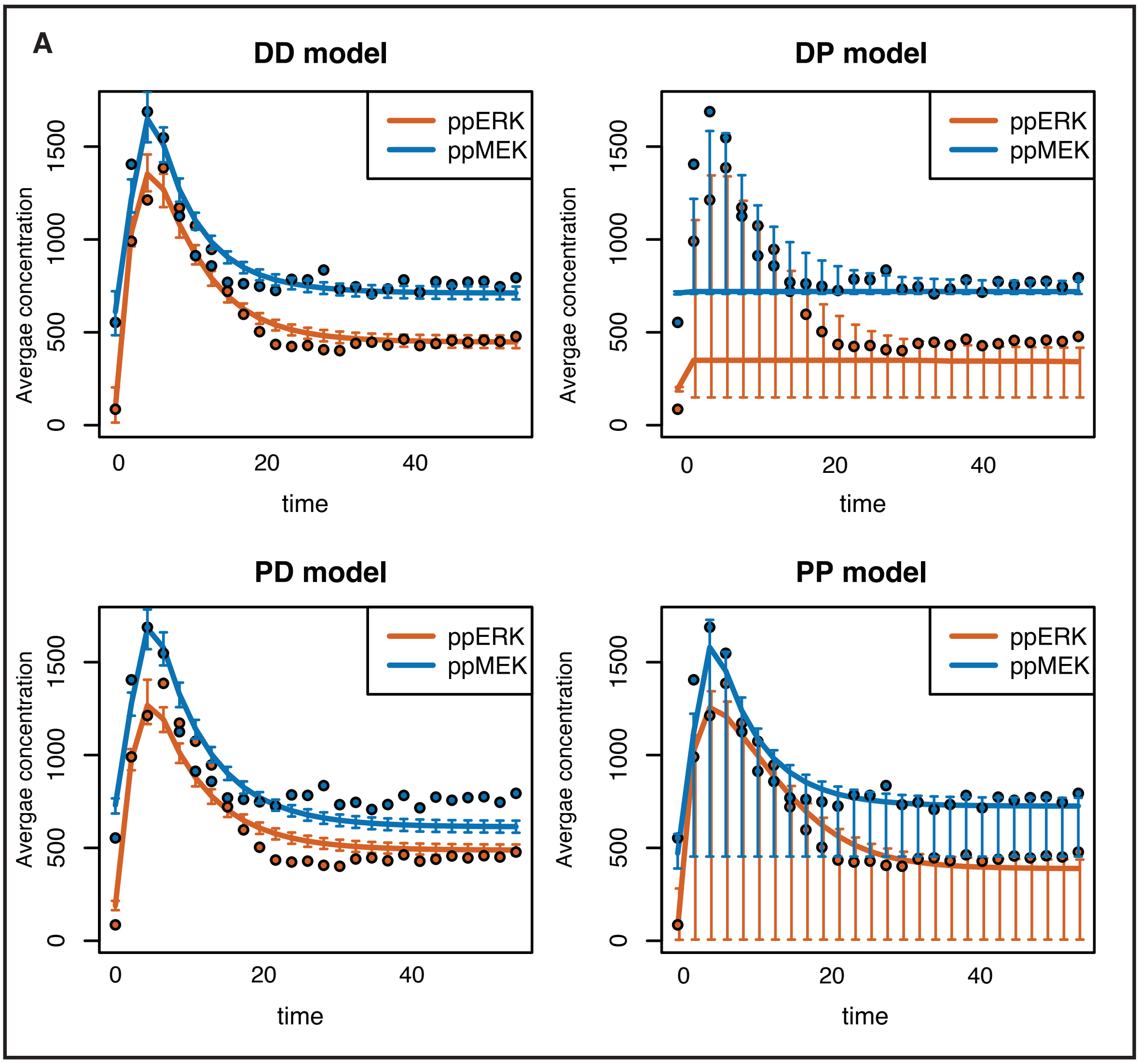

B

Model	log-evidence	Bayes factor wrt DD
DD	-227 (±0.8)	
DP	-264 (±8)	1.2x10^{16}
PD	-260 (±8)	2.2x10^{14}
PP	-245 (±3)	6.5x10^{7}

Figure S5: **The distributive phosphorylation and dephosphorylation mechanism best explains the average behavior. (Related to Figure 1) (A)** Fits to the average data (dots) for the four mechanistic models. The lines represent the median of the evolution of the average concentration of the two species (total amount of doubly phosphorylated ERK in orange and total amount of doubly phosphorylated MEK in blue) and the error bars designate the 0.025 and 0.975 quantiles for a set of 1000 parameters sampled from the posterior distribution. Both the DD and the DP models provide good visual fits. **(B)** Model ranking through the evidence shows that the DD model has the highest log-evidence and is therefore more strongly supported by the average data.

S2 Details on the implementation of the SMC sampler algorithm

Parameter inference is performed using the Sequential Monte Carlo sampler algorithm proposed by Del Moral et al. (2006), which is emerging as a powerful alternative to conventional Markov chain Monte Carlo (MCMC) methods (Robert and Casella, 2004). The algorithm sequentially generates samples from the probability distributions, $p(\theta)^{1-\phi}p(\theta|x^*)^{\phi}$, for ϕ varying from 0 to 1, by sampling parameter vectors from the prior distribution, $p(\theta)$, and exploiting the likelihood function in order to guide the set of parameter vectors toward a region of high posterior probability. The exact version of the algorithm we use is detailed below (see Algorithm 1). We use $N = 10^4$ particles per population. To determine the next value of ϕ at the beginning of each population (step 5), we ensure that the effective sample size (ESS), which is equal to $\left(\sum_{n=1}^{N} \omega_a^{(n)}\right)^{-1}$ and only depends on the previous weighted population $\{(\theta_{a-1}^{(n)}, \omega_{a-1}^{(n)})\}_{1\leq n\leq N}$ as well as on ϕ and ϕ_{old}, is between 0.5 and 0.9. In addition, to perturb the particles (step 10), we use an MCMC kernel which consists of 5 steps of Metropolis Hasting perturbations with an adaptive multi-variate normal random-walk proposal (Del Moral et al., 2006).

Algorithm 1: The SMC sampler

Input: No. of particles per population N.
Output: Set of weighted particles $\{\theta_p^{(n)}, \omega_p^{(n)}\}_{1\leq n\leq N}$.

1 Initialise $a = 1$, $\phi = 0$;
2 Sample particles $\theta_1^{(n)}$ from prior ;
3 Set weights $\omega_1^{(n)} = \frac{1}{N}$;
4 **while** $\phi < 1$ **do**
5   Set $\phi_{old} = \phi$; a = a+1;
6   Determine next value of ϕ;
7   Resample particles $\{\theta_{a-1}^{(n)}\}$ from weighted multinomial distribution $\{(\theta_{a-1}^{(n)}, \omega_{a-1}^{(n)})\}$;
8   Reset weights $\omega_{a-1}^{(n)} = \frac{1}{N}, \forall n = 1, \ldots, N$;
9   **for** $1 \leq n \leq N$ **do**
10   Draw $\theta_a^{(n)} \sim K_a(\cdot \mid \theta_{a-1}^{(n)})$, where K_a is a MCMC kernel;
11   Update particle weight $\widetilde{\omega}_a^{(n)} = \omega_{a-1}^{(n)} \cdot p(\theta_{a-1}^{(n)}|x^*)^{\phi-\phi_{old}}$;
12   **end**
13   Normalise particle weights $\omega_a^{(n)} = \widetilde{\omega}_a^{(n)} / \sum_{m=1}^{N} \widetilde{\omega}_a^{(m)}$.
14 **end**

S3 The intrinsic noise model

The Linear Noise Approximation (LNA) is used to define a likelihood function in the case of the intrinsic noise model. It is a Gaussian approximation to Markov Jump processes defined by the Chemical Master Equation (Komorowski et al., 2009). Biochemical reactions are modelled through a stochastic dynamic model and the LNA provides us with equations for the average behaviour over the population of cells as well as the evolution of the variance and covariances with time. With $m_t^x(\theta)$, $m_t^y(\theta)$, $v_t^x(\theta)$ and $v_t^y(\theta)$ denoting the solutions of the ODEs describing the means and variances for the parameter θ at time t, the likelihood $p(\{x_{i,t}^*, y_{i,t}^*\}_{i,t}|\theta)$ is equal to

$$\prod_{t\in\mathbb{T}}\prod_{i=1}^{N_t}\Phi(x_{i,t}^*; m_t^x(\theta), v^x(\theta))\Phi(y_{i,t}^*; m_t^y(\theta), v^y(\theta))$$

where $\Phi(\cdot; m, v)$ is the probability density function of a normal distribution of mean m and variance v.

S3.1 Investigating the precision of the Linear Noise Approximation

In order to investigate the accuracy of the Linear Noise Approximation, we generated 100 trajectories simulating the DD model with the Gillespie algorithm (which took around 12 CPU days per trajectory), computed the evolution of the mean and the variance over the 100 trajectories and compared it to the solution of the ODE equations provided by the LNA. As can be seen in Figure S6A, the Linear Noise approximations of the evolution of the means and the variances are very accurate.

S3.2 Efficient sampling of the parameter space

In the main manuscript, we demonstrate that we can confidently implicate extrinsic noise as the dominant factor giving rise to cell-to-cell variability in the MEK/ERK module and that intrinsic noise does not explain the level of observed cell-to-cell variability. To substantiate this further (and to ensure that we explore the parameter space more widely during the parameter inference step of the intrinsic noise) we use Latin hyper-cube sampling (McKay et al., 1979) to generate a set of 10^6 parameter vectors using the Matlab function *lhsdesign.* and systematically analyse the evolution of the molecular concentrations of MEK and ERK for each of these parameters. Only 20 parameter vectors out of the 10^6 lead to stable solutions for which the obtained variances of doubly phosphorylated ERK and MEK is higher than 10^5 (at either 6 or 8 minutes after stimulation; but for none of these parameters do we observe a variance of doubly phosphorylated ERK that is anywhere close to the experimental observations.

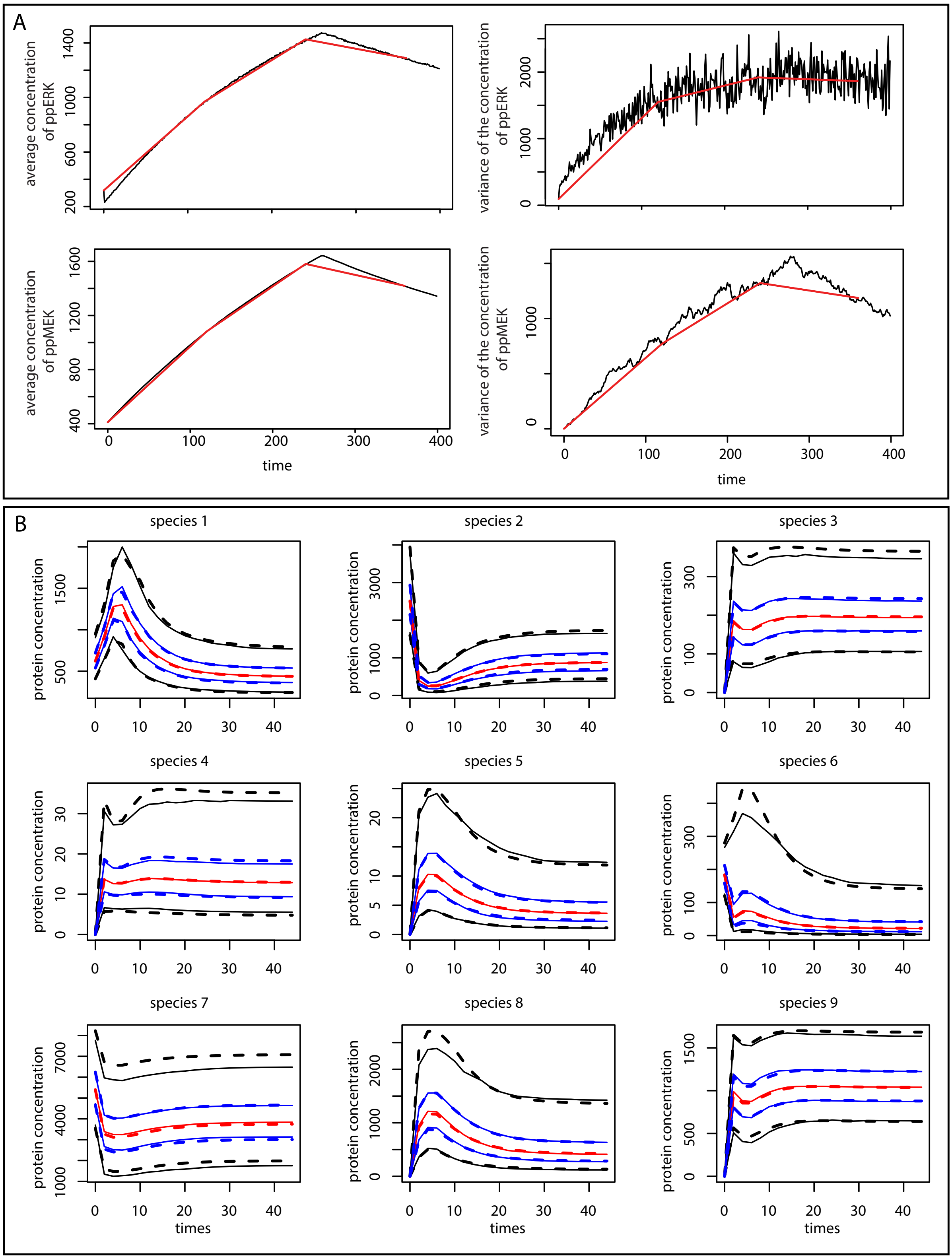

Figure S6: **Accuracy of likelihood approximations. (Related to Figures 3 and 4)** **(A)**The evolution of the mean and variance for the two molecular species of interest. The black lines corresponds to the mean and variances over the trajectories simulated using the Gillespie algorithm and the red lines are the approximation provided by the LNA. **(B)** Comparison of the distribution of the evolution of the protein concentrations (for all the 9 species) for one set of hyper-parameters given by the UT method and by a Monte-Carlo approximation with 1000 particles. Each plot is associated with one of the 9 species. The continuous lines correspond to the Monte-Carlo approximation and the dashed lines correspond to the UT approximation. The red lines represent the median, the blue lines represent the 0.25 and 0.75 quantiles and the black lines represent the 0.025 and the 0.975 quantiles of the distributions.

S4 The extrinsic noise model

S4.1 Using Unscented Transform to derive a likelihood function for the extrinsic noise model

To mathematically describe the cell-to-cell variability due to extrinsic noise a different parameter vector is associated with each cell. Recall that for each time $t \in \mathbb{T} = \{0, 2, 4, \ldots 50\}$, we measure the total amount of doubly phosphorylated MEK and ERK, denoted respectively by $x^*_{i,t}$ and $y^*_{i,t}$, in cells $1 \le i \le N_t$. Let denote by $\theta_{i,t}$ the vector of parameters associated to the i-th cell measured at time t. With a single measurement per cell, it is not possible to infer every single parameter $\theta_{i,t}$. Instead, we study the distribution of the parameters $\{\theta_{i,t}\}_{i,t}$; here, we consider a log-normal distribution with mean μ_θ and covariance matrix Σ_θ.

We aim at inferring the hyper-parameters μ_θ and Σ_θ of the constructed hierarchical model given the observed data $\{x^*_{i,t}, y^*_{i,t}\}_{i,t}$. Note that the model contains 20 parameters (see section S1.2) therefore μ_θ is of length 20 and Σ_θ is a 20×20 symmetric matrix. Due to the prohibitive computational cost of inferring these 210 values, we simplify the model by assuming that the covariance matrix Σ_θ is diagonal; we denote by σ^2_θ the vector containing the diagonal elements. The likelihood function is defined as follows

$$p(\{x^*_{i,t}, y^*_{i,t}\}_{i,t} | \mu_\theta, \sigma^2_\theta) = \prod_t \prod_i \int \mathbb{1}_{(x^*_{i,t}, y^*_{i,t}) = f(\theta_{i,t}, t)} p(\theta_{i,t} | \mu_\theta, \sigma^2_\theta) d\theta_{i,t} \tag{1}$$

where $f(\theta_{i,t}, t)$ describes the simulated concentration of the two species of interest at time t when simulating the model with parameter $\theta_{i,t}$; as described above $p(\theta_{i,t} | \mu_\theta, \sigma^2_\theta)$ is the density of a log-normal distribution with mean μ_θ and covariance matrix $\Sigma_\theta = \text{diag}(\sigma^2_\theta)$. This likelihood function can not be computed in closed-form and needs to be estimated. We propose to use the *Unscented Transform* (UT) (Julier, 2002) to approximate the two moments of the distribution $p(\{x^*_{i,t}, y^*_{i,t}\}_{i,t} | \mu_\theta, \Sigma_\theta)$. We denote by $m^x_t(\mu_\theta, \sigma^2_\theta)$ (resp. $m^y_t(\mu_\theta, \sigma^2_\theta)$) and $v^x_t(\mu_\theta, \sigma^2_\theta)$ (resp. $v^y_t(\mu_\theta, \sigma^2_\theta)$) the mean and the variance of this distributions as a function of the hyper–parameters μ_θ and σ^2_θ .

The UT is a mathematical tool, which allows us to approximate the moments of the output of a non-linear function given the moments of the input. The first step of the UT algorithm is to determine a set of weighted particles $\{\xi_j\}_j$, called *sigma points*, which capture both the mean μ_θ and the variances σ^2_θ. Since we assume a log-normal distribution over the parameter space, we consider the variable $\tilde{\theta}$, which is distributed according to a normal distribution with mean $\tilde{\mu}_\theta = \log(\mu_\theta) - 1/2 \log(\sigma^2_\theta / \mu^2_\theta + 1)$ and variance $\tilde{\sigma}^2_\theta = \log(\sigma^2_\theta / \mu^2_\theta + 1)$. Denoting by D the dimension of the parameter space, the sigma-points are defined as follows:

$$\begin{aligned}
\xi_0 &= \tilde{\mu}_\theta \\
\xi_j &= \tilde{\mu}_\theta + \alpha\sqrt{D+\kappa}\,[\tilde{\sigma}_\theta]_j && j = 1, \cdots, D \\
\xi_j &= \tilde{\mu}_\theta - \alpha\sqrt{D+\kappa}\,[\tilde{\sigma}_\theta]_j && j = D+1, \cdots, 2D,
\end{aligned}$$

where $[\tilde{\sigma}_\theta]_j$ represents a vector full of zeros except on the j-th element which is equal to the j-the element of the vector $\tilde{\sigma}_\theta$. The sigma-point weights $\{w^m_j, w^v_j\}_{0 \le j \le 2D}$ are given by,

$$\begin{aligned}
w^m_0 &= \frac{\alpha^2(D+\kappa) - D}{\alpha^2(D+\kappa)} \\
w^v_0 &= \frac{\alpha^2(D+\kappa) - D}{\alpha^2(D+\kappa)} + 1 - \alpha^2 + \beta \\
w^m_j &= w^v_j = \frac{1}{2\alpha^2(D+\kappa)} && j = 1, \cdots, 2D\,.
\end{aligned}$$

The parameters κ, α and β may be chosen to control the positive definiteness of the covariance matrices, spread of the sigma-points and error in the kurtosis respectively (Silk, 2013). Here we use $\kappa = 0$, $\alpha = 0.7$ and $\beta = 2$.

Once the sigma-points have been determined, the ODE system is solved for each sigma-points separately. More precisely, for each $0 \leq j \leq 2D$, we solve the ODE system with the parameter $\exp(\xi_j)$, resulting in solutions $x_{j,t}$ and $y_{j,t}$. Assuming a log-normal distribution in the molecular concentration space, the means and variances of the distribution at each time point can then be computed as

$$m_t^x(\mu_\theta, \sigma_\theta^2) = \sum_{j=0}^{2D} w_j^m \log(x_{j,t})$$

$$m_t^y(\mu_\theta, \sigma_\theta^2) = \sum_{j=0}^{2D} w_j^m \log(y_{j,t})$$

$$v_t^x(\mu_\theta, \sigma_\theta^2) = \sum_{j=0}^{2D} w_j^v (\log(x_{j,t}) - m_t^x(\mu_\theta, \sigma_\theta^2))^2$$

$$v_t^y(\mu_\theta, \sigma_\theta^2) = \sum_{j=0}^{2D} w_j^v (\log(x_{j,t}) - m_t^y(\mu_\theta, \sigma_\theta^2))^2 \ .$$

Therefore the likelihood in equation (1) is approximated as follows

$$p(\{x_{i,t}^*, y_{i,t}^*\}_{i,t} | \mu_\theta, \sigma_\theta^2) = \prod_{t \in \mathbb{T}} \prod_{i=1}^{N_t} \Psi\left(x_{i,t}^*; m_t^x(\mu_\theta, \sigma_\theta^2), v_t^x(\mu_\theta, \sigma_\theta^2)\right) \Psi\left(y_{i,t}^*; m_t^y(\mu_\theta, \sigma_\theta^2), v_t^y(\mu_\theta, \sigma_\theta^2)\right)$$

where $\Psi(\cdot)$ is the pdf of a log-normal distribution.

In Figure S6B we compare the approximation of the likelihood function $p(\{x_{i,t}^*, y_{i,t}^*\}_{i,t} | \mu_\theta, \sigma_\theta^2)$ given by the UT algorithm to a Monte-Carlo approximation. In the Monte-Carlo approximation, 1000 set of parameters are sampled from log-normal distributions (with mean μ_θ and variance σ_θ^2). For each parameter set, we solve the system of ODE to obtain trajectories of every species in the system and then we compute the median and quantiles of the obtained trajectories.

S4.2 Extrinsic noise: identification of model parameters that significantly vary between cells

Under the extrinsic noise model, all model parameters differ between cells. In each cell every parameter, k, is drawn from a log-normal distribution with mean μ_k and variance σ_k^2 (called hyper-parameters). Our Bayesian inference procedure based on the single-cell data allows us to obtain posterior distributions for every hyper-parameters. For the DD model, there are 20 model parameters and therefore 40 hyper-parameters.

To investigate which parameters contribute most to the observed cell-to-cell variability, we analyse the posterior distribution of the coefficient of variation for each parameter (σ_k/μ_k for each k) shown in Figure S7. The coefficients of variation take value between 0 and 1. We distinguish 3 types of posterior distributions: (i) posterior distributions with a support that covers more than 60% of $[0, 1]$ (framed in blue), (ii) posterior distributions close to 0 (posterior framed in yellow has 0.25th percentile lower than 0.05 and a 0.75th percentile lower than 0.3), and (iii) the other posteriors distributions which are more tightly constrained and significantly different to 0 (framed in red). The parameters that contribute most to the observed cell-to-cell variability are those for which the posterior distribution of the coefficient of variation is consistently and significantly different from zero. Indeed, a posterior distribution of a coefficient of variation very close to 0 indicates that the model parameter does not need to vary between cells; a posterior of a coefficient of variation not constrained and including most of the $[0, 1]$ support demonstrates that the variation of this model parameter between cells is not crucial to explain the level of cell-to-cell variability. Therefore only the parameters framed in red in Figure S7 appear to play an important role in the cell-to-cell variability. These parameters are k_1, k_2, k_{10}, ppE_0 and M_0.

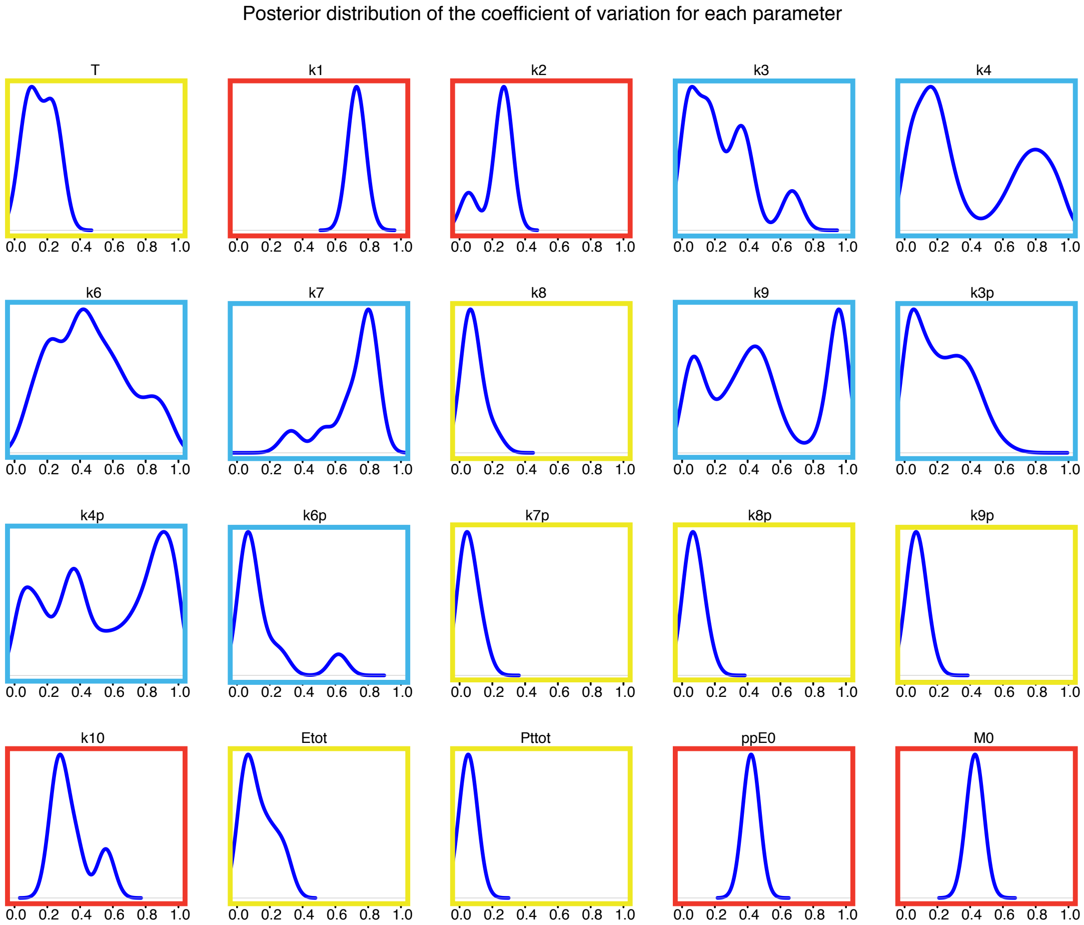

Figure S7: **Posterior distribution of coefficient of variation. (Related to Figures 4, 5 and 6)** The posterior distribution obtained using SMC sampler for the coefficient of variation of each parameter are shown here. We distinguish 3 types of posterior distributions: (i) posterior distributions with a support that covers more than 60% of $[0, 1]$ (framed in blue), (ii) posterior distributions close to 0 (posterior framed in yellow has 0.25th percentile lower than 0.05 and a 0.75th percentile lower than 0.3), and (iii) the other posteriors distributions which are more tightly constrained and significantly different to 0 (framed in red).

Supplemental References

Del Moral, P., Doucet, A. and Jasra, A. (2006). Sequential monte carlo samplers. Journal of the Royal Statistical Society: Series B (Statistical Methodology) *68*, 411–436.

Ferrell, J. E. and Bhatt, R. R. (1997a). Mechanistic studies of the dual phosphorylation of mitogen-activated protein kinase. Journal of Biological Chemistry *272*, 19008–19016.

Ferrell, J. E. and Bhatt, R. R. (1997b). Mechanistic studies of the dual phosphorylation of mitogen-activated protein kinase. Journal of Biological Chemistry *272*, 19008–19016.

Gunawardena, J. (2007). Distributivity and processivity in multisite phosphorylation can be distinguished through steady-state invariants. Biophysical journal *93*, 3828–3834.

Julier, S. J. (2002). The scaled unscented transformation. In American Control Conference, Proceedings of the IEEE vol. 6, pp. 4555–4559,.

Kolaczyk, E. D. (2009). Statistical Analysis Of Network Data: Methods And Models. Springer.

Komorowski, M., Finkenstädt, B., Harper, C. and Rand, D. (2009). Bayesian inference of biochemical kinetic parameters using the linear noise approximation. BMC bioinformatics *10*, 343.

McKay, M. D., Beckman, R. J. and Conover, W. J. (1979). Comparison of three methods for selecting values of input variables in the analysis of output from a computer code. Technometrics *21*, 239–245.

Robert, C. P. and Casella, G. (2004). Monte Carlo statistical methods, vol. 319,. Citeseer.

Schäfer, J., Opgen-Rhein, R. and Strimmer, K. (2001). Reverse engineering genetic networks using the GeneNet package. Journal of the American Statistical Association *96*, 1151–1160.

Schäfer, J. and Strimmer, K. (2005). A shrinkage approach to large-scale covariance matrix estimation and implications for functional genomics. Statistical applications in genetics and molecular biology *4*, Article32.

Silk, D. (2013). Unscented approaches to inference and design for systems and synthetic biology. PhD thesis, Imperial College London.

Thorne, T. W., Fratta, P., Hanna, M. G., Cortese, A., Plagnol, V., Fisher, E. M., Stumpf, M. P. H. and Stumpf, M. P. H. (2013). Graphical modelling of molecular networks underlying sporadic inclusion body myositis. Molecular Biosystems *9*, 1736–1742.

Toni, T., Ozaki, Y.-i., Kirk, P., Kuroda, S. and Stumpf, M. P. H. (2012). Elucidating the in vivo phosphorylation dynamics of the ERK MAP kinase using quantitative proteomics data and Bayesian model selection. Molecular BioSystems *8*, 1921–1929.

Uda, S., Saito, T. H., Kudo, T., Kokaji, T., Tsuchiya, T., Kubota, H., Komori, Y., Ozaki, Y.-i. and Kuroda, S. (2013). Robustness and compensation of information transmission of signaling pathways. Science *341*, 558–561.
