# Supplementary material for: Robustness of MEK-ERK Dynamics and Origins of Cell-to-Cell Variability in MAPK Signaling
Source: Cell Rep. 2016 Jun 2;15(11):2524–35. doi: 10.1016/j.celrep.2016.05.024 (PMC4914773; doi:10.1016/j.celrep.2016.05.024)
Supplement: Document S2. Article plus Supplemental Information [file mmc2.pdf]

Cell Reports

Article

Robustness of MEK-ERK Dynamics and Origins of Cell-to-Cell Variability in MAPK Signaling

Graphical Abstract

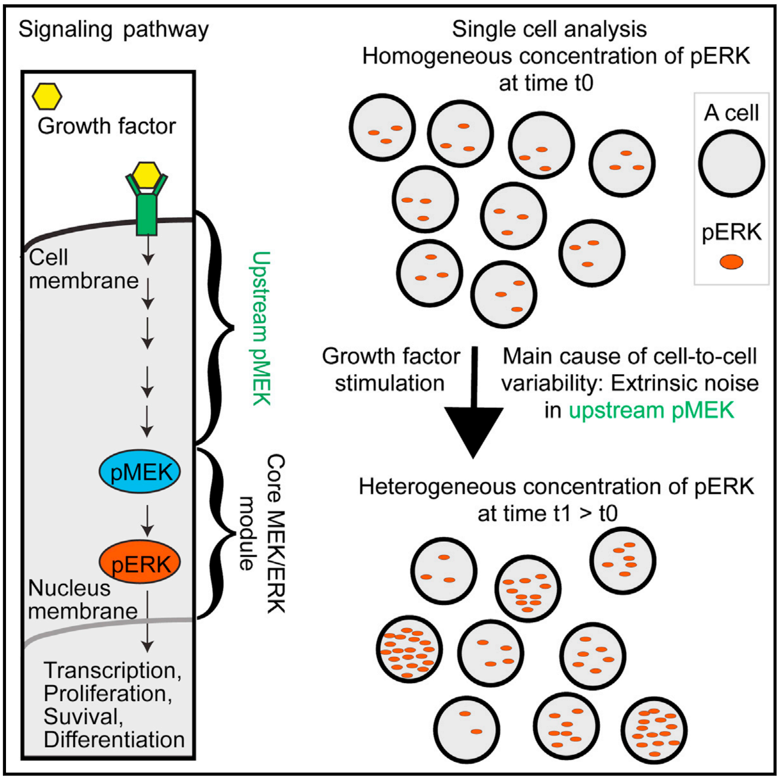

Highlights

- Active MEK and ERK levels differ profoundly among genetically identical cells
- A statistical framework is developed to identify the causes of this variability
- Analysis shows that extrinsic noise upstream MEK-ERK module causes cell variability
- Within-module extrinsic variability distorts signals

Authors

Sarah Filippi, Chris P. Barnes, Paul D.W. Kirk, ..., Takumi Wada, Shinya Kuroda, Michael P.H. Stumpf

Correspondence

m.stumpf@imperial.ac.uk

In Brief

Cellular signaling processes can exhibit pronounced cell-to-cell variability in genetically identical cells, but the origins of such variability remain poorly understood. Filippi et al. present a comprehensive analysis of cell-to-cell variability in the ERK phosphorylation process by combining a statistical modeling approach with high-throughput image cytometry measurements.

Filippi et al., 2016, Cell Reports *15*, 2524–2535
June 14, 2016 © 2016 The Author(s).
http://dx.doi.org/10.1016/j.celrep.2016.05.024

CellPress

OPEN ACCESS
CellPress

Cell Reports
Article

Robustness of MEK-ERK Dynamics and Origins of Cell-to-Cell Variability in MAPK Signaling

Sarah Filippi,[1] Chris P. Barnes,[2,3] Paul D.W. Kirk,[1] Takamasa Kudo,[4] Katsuyuki Kunida,[4] Siobhan S. McMahon,[1] Takaho Tsuchiya,[4] Takumi Wada,[4] Shinya Kuroda,[4,5] and Michael P.H. Stumpf[1,6,*]
[1]Centre for Integrative Systems Biology and Bioinformatics, Imperial College London, London SW7 2AZ, UK
[2]Department of Cell and Developmental Biology, University College London, London WC1E 6BT, UK
[3]Department of Genetics, Evolution and Environment, University College London, London WC1E 6BT, UK
[4]Department of Biological Sciences, Graduate School of Science, University of Tokyo, Tokyo 113-8654, Japan
[5]CREST, Japan Science and Technology Agency, Bunkyo-ku, Tokyo 113-0033, Japan
[6]Institute of Chemical Biology, Imperial College London, London SW7 2AZ, UK
*Correspondence: m.stumpf@imperial.ac.uk
http://dx.doi.org/10.1016/j.celrep.2016.05.024

SUMMARY

Cellular signaling processes can exhibit pronounced cell-to-cell variability in genetically identical cells. This affects how individual cells respond differentially to the same environmental stimulus. However, the origins of cell-to-cell variability in cellular signaling systems remain poorly understood. Here, we measure the dynamics of phosphorylated MEK and ERK across cell populations and quantify the levels of population heterogeneity over time using high-throughput image cytometry. We use a statistical modeling framework to show that extrinsic noise, particularly that from upstream MEK, is the dominant factor causing cell-to-cell variability in ERK phosphorylation, rather than stochasticity in the phosphorylation/dephosphorylation of ERK. We furthermore show that without extrinsic noise in the core module, variable (including noisy) signals would be faithfully reproduced downstream, but the within-module extrinsic variability distorts these signals and leads to a drastic reduction in the mutual information between incoming signal and ERK activity.

INTRODUCTION

The behavior of eukaryotic cells is determined by an intricate interplay between signaling, gene regulation, and epigenetic processes. Within a cell, each single molecular reaction occurs stochastically, and the expression levels of molecules can vary considerably in individual cells (Bowsher and Swain, 2012). These non-genetic differences frequently add up to macroscopically observable phenotypic variation (Spencer et al., 2009; Balázsi et al., 2011; Spiller et al., 2010). Such variability can have organism-wide consequences, especially when small differences in the initial cell populations are amplified among their progeny (Quaranta and Garbett, 2010; Pujadas and Feinberg, 2012). Cancer is the canonical example of a disease caused by a sequence of chance events that may be the result of amplifying physiological background levels of cell-to-cell variability (Roberts and Der, 2007).

Better understanding of the molecular mechanisms behind the initiation, enhancement, attenuation, and control of this cellular heterogeneity should help us to address a host of fundamental questions in cell biology and experimental and regenerative medicine. Noise at the molecular level has been amply demonstrated in the literature, in the contexts of both gene expression (Elowitz et al., 2002; Swain et al., 2002; Hilfinger and Paulsson, 2011) and signal transduction (Colman-Lerner et al., 2005; Jeschke et al., 2013). The molecular causes underlying population heterogeneity are only beginning to be understood, and each new study adds nuance and detail to our emerging understanding. Two notions have come to dominate the literature: intrinsic and extrinsic causes of cell-to-cell variability (Swain et al., 2002; Komorowski et al., 2010; Hilfinger and Paulsson, 2011; Toni and Tidor, 2013; Bowsher and Swain, 2012). The former refers to the chance events governing the molecular collisions in biochemical reactions. Each reaction occurs at a random time leading to stochastic differences between cells over time. The latter subsumes all those aspects of the system that are not explicitly modeled. This includes the impact of stochastic dynamics in any components upstream and/or downstream of the biological system of interest, which may be caused, for example, by the stage of the cell cycle and the multitude of factors deriving from it.

It has now become possible to track populations of eukaryotic cells at single-cell resolution over time and measure the changes in the abundances of proteins (Selimkhanov et al., 2014). For example, rich temporal behavior of p53 (Geva-Zatorsky et al., 2006; Batchelor et al., 2011) and Nf-κb (Nelson et al., 2004; Ashall et al., 2009; Paszek et al., 2010) has been characterized in single-cell time-lapse imaging studies. Given such data, and with a suitable model for system dynamics and extrinsic noise in hand it is possible, in principle, to locate the causes of cell-to-cell variability and quantify their contributions to system dynamics. Here, we develop a statistical framework for just this purpose, and we apply it to measurements obtained by quantitative image cytometry (Ozaki et al., 2010): data are obtained at discrete time points but encompass thousands of cells, which allows one to investigate the causes of cell-to-cell variability (Johnston, 2014). The in silico statistical model selection framework

This is an open access article under the CC BY license (http://creativecommons.org/licenses/by/4.0/).

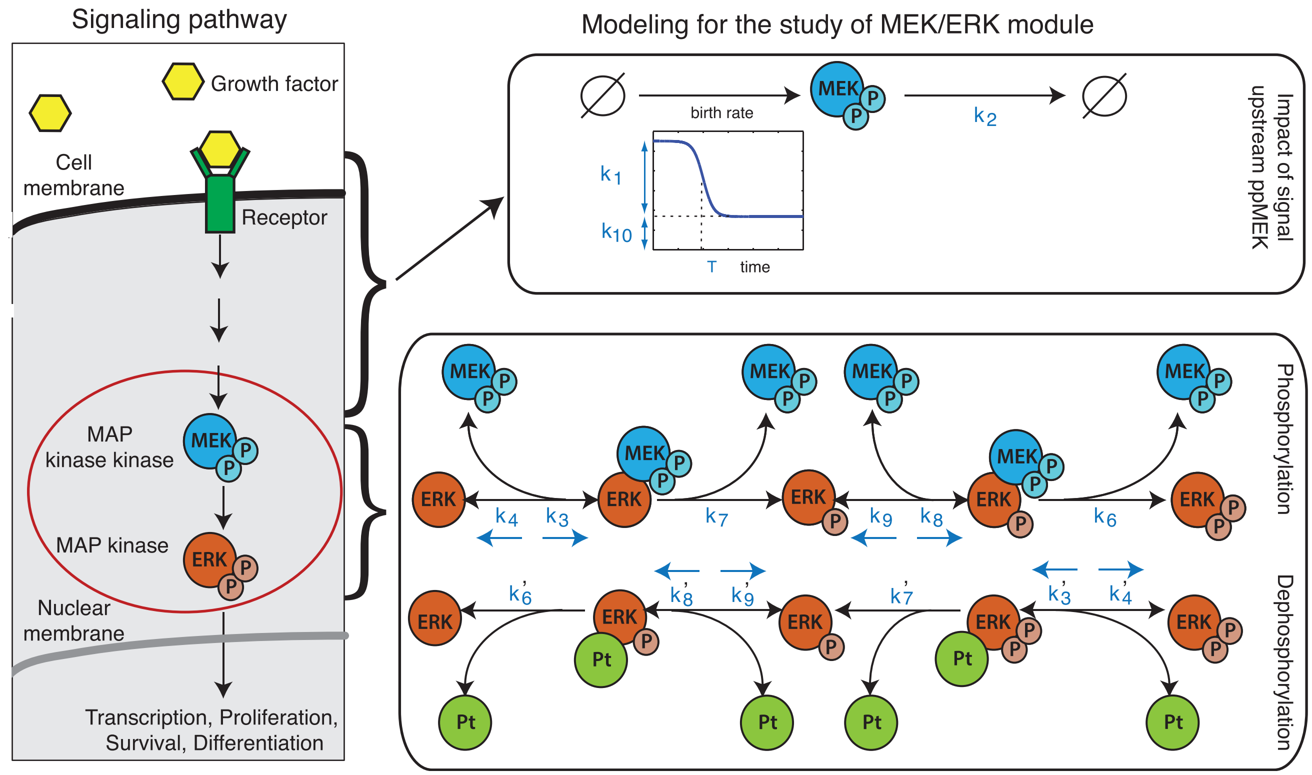

Figure 1. The MEK/ERK System and Modeling of the MEK/ERK Module

The binding of a growth factor to its receptor activates a succession of reactions that lead to the phosphorylation of MEK; active MEK, in turn, phosphorylates ERK. In this study, we focus on the MEK/ERK module (circled in red). The impact of the stimulus and the upstream reactions on the evolution of the concentration of active MEK are modeled using a time-dependent function, which depends on three parameters (k_1, k_{10}, and T). In addition, active MEK is degraded with rate k_2. The detailed mechanism of phosphorylation and dephosphorylation of ERK is represented in the bottom-right part of the figure. Pt denotes the cognate ERK phosphatase. The reaction rates are shown next to their associated reactions.

also has the advantage that it can be applied in situations where, e.g., dual reporter assays, which explicitly separate out extrinsic and intrinsic sources of variability (Hilfinger and Paulsson, 2011), cannot be applied.

With this framework in hand we consider the dynamics of the central MEK-ERK core module of the MAPK signaling cascade, see Figure 1 (Santos et al., 2007; Inder et al., 2008). MAPK mediated signaling affects cell-fate decision-making processes (Eser et al., 2011)—including proliferation, differentiation, apoptosis, and cell stasis—and cell motility, and the mechanisms of MAPK cascades and their role in cellular information processing have been investigated extensively (Kiel and Serrano, 2009; Mody et al., 2009; Sturm et al., 2010; Takahashi et al., 2010; Aoki et al., 2011; Piala et al., 2014; Voliotis et al., 2014). Here, we take an engineering perspective and aim to characterize how MEK and ERK transmit signals. The upstream sources of noise in signaling involving MAPK cascades have been amply documented (see, e.g., Schoeberl et al., 2002; Santos et al., 2012; Sasagawa et al., 2005), as have their downstream consequences, e.g., in the context of stem cell-fate decision making (Miyanari and Torres-Padilla, 2012; Schröter et al., 2015). The manner in which MEK and ERK modulate this variability is less well understood in detail. Our aim is to answer three related questions: (1) are the dynamics of the MEK-ERK module noisy; (2) where might this noise originate; and (3) how does noise in the MEK-ERK system affect the ability of this important molecular system to relay information reliably?

Below we will first quantify the levels of cell-to-cell variability sources of noise in the system, with a special focus on the dynamics of active, i.e., phosphorylated, MEK and ERK; after this we will identify the sources of such noise and compare their relative contributions to cell-to-cell variability. We will show that our analysis is robust to both qualitative as well as quantitative changes in the upstream stimulation. With this in hand, we can then turn to an investigation of the effects cell-to-cell variability has on the ability of cell populations to respond to fluctuating signals.

RESULTS

Quantifying Temporal Evolution of Cell-to-Cell Variability

We investigate the causes of cellular heterogeneity in vivo during ERK activation by phosphorylated MEK in PC12 cells. This cell-to-cell variability study is based on measurements of the concentration of phosphorylated MEK and ERK at the single-cell level obtained by quantitative image cytometry. Cells are plated in medium containing a fixed amount of neuronal growth factor

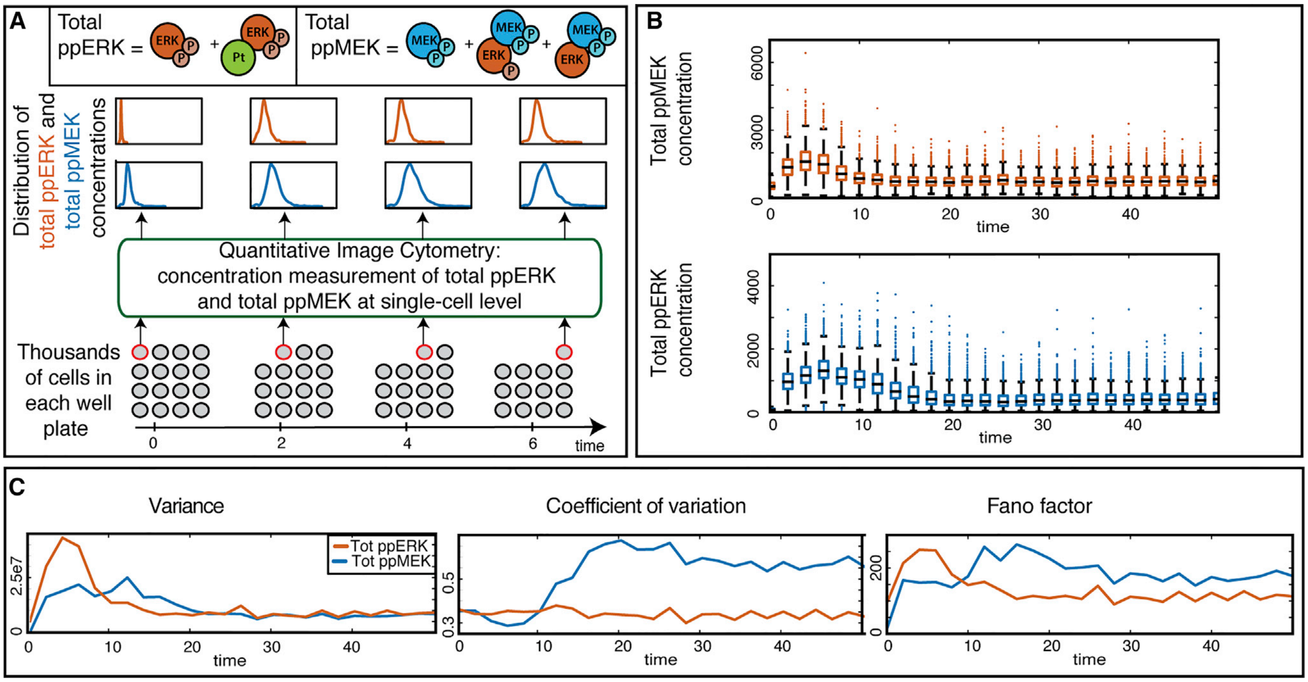

Figure 2. Measurement of the Evolution of the Joint Protein Distributions of Phosphorylated MEK and Phosphorylated ERK

(A) Cells are plated in a medium and stimulated with NGF at $t = 0$. Every 2 min, thousands of cells are stimulated and the amount of total phosphorylated MEK and ERK (i.e., the sum of free and complex-bound forms) is measured at single-cell level using quantitative image cytometry, providing a series of cross-sectional snapshots of the joint distributions of the level of phosphorylated MEK and ERK.

(B) Boxplots showing the distributions of the measured protein concentrations at each time point (the edges of the colored boxes are the 0.25 and 0.75 quantiles; the central mark is the median).

(C) The temporal evolution of the variance, the coefficient of variation and the Fano factor for the distributions of the total amount of phosphorylated MEK and ERK.

(NGF) as the stimulus at time t = 0. Every 2 min, cells in one well are stimulated in order to quantify the concentration of the two proteins of interest providing us with a series of cross-sectional snapshots of the joint protein distributions of the total amount of phosphorylated MEK and ERK, see Figure 2A.

The observed distributions of the total amount of phosphorylated MEK and ERK are illustrated in Figures 2B and S1, and Figure 2C shows the evolution of the variance, the coefficient of variation and the Fano factor over time for both proteins. The variance over the cell population of the concentration is of the order of 10^5 and significantly varies with time. We have examined experimental noise versus cell-to-cell variability of total ERKs in unstimulated PC12 cells (Figure S2) (Uda et al., 2013) and found that the experimental noise is negligible. In addition, cell size, cell volume, and Hoechst level (the dye used to quantify nucleic acid levels) make only negligible contributions to observed levels of cell-to-cell variability (Figure S3). We can thus rule out cell cycle, etc. as explanations for or cause of the temporal variability in the amount of active ERK.

Statistical Investigation of the Cell-to-Cell Variability in the Core MEK-ERK Module

The analysis of the origins of cell-to-cell variability in the core MEK-ERK module (i.e., the MEK-ERK interactions as indicated by the red circle in Figure 1, left panel) requires us to determine the modes of ERK phosphorylation and dephosphorylation. ERK activation involves phosphorylation at both its tyrosine and threonine phosphorylation sites by its cognate kinase MEK (Ferrell and Bhatt, 1997; Ferrell and Ha, 2014). Previous studies (Toni et al., 2012) have shown that in vivo phosphorylation (as well as dephosphorylation) occurs in two steps where the kinase binds to the protein twice in order to phosphorylate the two sites successively (see Figure 1, bottom right). Using a Bayesian model selection approach, we confirm that this distributive mechanism best captures the observed average behavior in our data (see Figure S2). We therefore base our analysis of the origins of cell-to-cell variability on this mechanistic model with 20 model parameters including 12 reaction rates, four parameters describing the impact of the NGF stimulus and upstream signals and four parameters controlling the initial concentrations of the species involved in the MEK-ERK core system (see Figure 1 and Supplemental Experimental Procedures).

In this model of the MEK-ERK core module, we assume that the total abundance of ERK remains constant over the length of the experiment and is described by one of the model parameters. Previously, we had shown experimentally that total abundance of ERK does not change, while the levels of phosphorylation change considerably (Ozaki et al., 2010); therefore, it is indeed appropriate to model the cell-to-cell variability of total ERK as (extrinsic) parameter variability.

The workflow adopted in this analysis is summarized in Figure 3. Given the mechanistic model of ERK phosphorylation

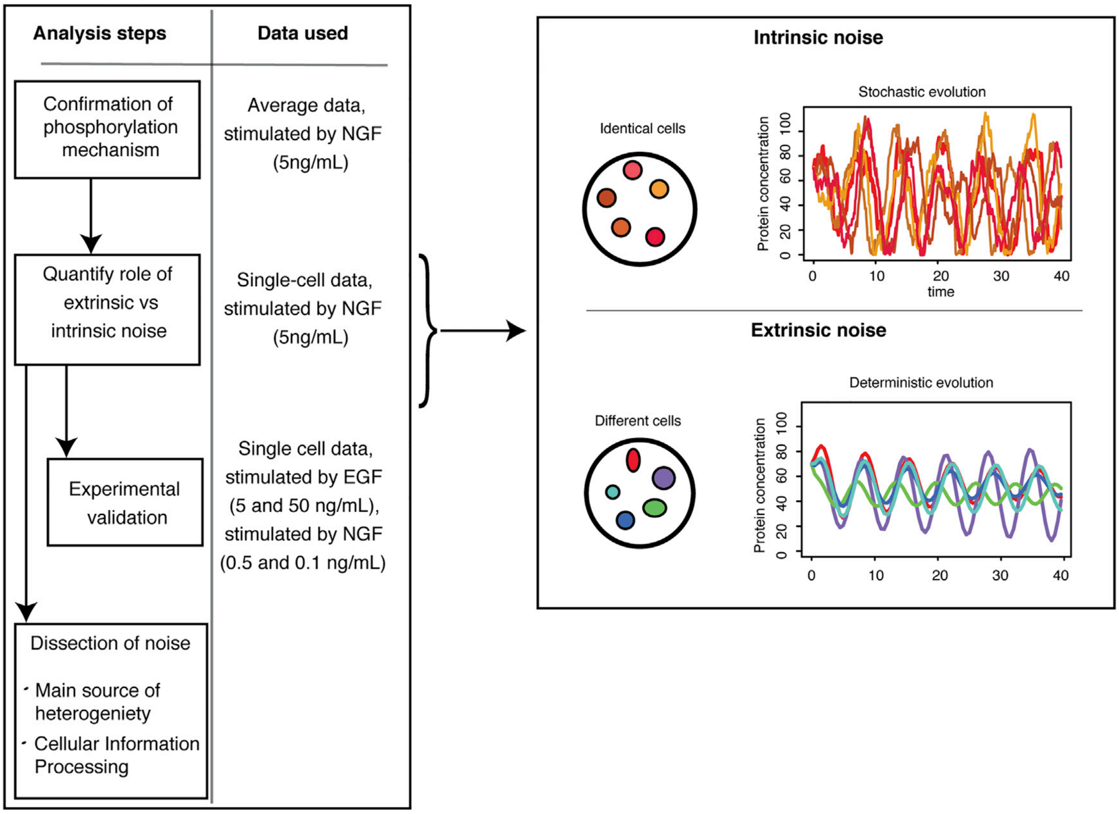

Figure 3. Elucidating the Origin of Cell-to-Cell Variability

(Left) Flowchart of the analysis of origin of cell-to-cell variability as performed in this paper, highlighting which data are used at each steps.

(Right) Within-cell variability can be caused by intrinsic noise, resulting from the stochastic nature of biochemical reactions, or extrinsic noise, arising from inherent differences between the cells.

described above, we will start by quantifying the relative contribution of intrinsic noise and extrinsic noise in the MEK-ERK core module. As illustrated in Figure 3, intrinsic noise results from the stochastic nature of biochemical reactions, while extrinsic noise arises from inherent differences between the cells. We will then experimentally validate our model of cell-to-cell variability by considering the response of the MEK-ERK system to different stimuli, and we will finish with a detailed analysis of the main source of cellular heterogeneity in the MEK-ERK core and the overall impact on MAPK-mediated cellular information processing.

Relative Contribution of Extrinsic and Intrinsic Noise in the MEK-ERK Core Module

While it is straightforward to model extrinsic and intrinsic noise, quantifying their relative contributions to real molecular systems has thus far only been possible for systems where two-reporter assays are available (Elowitz et al., 2002; Swain et al., 2002). Here, we develop a statistical framework that allows us to obtain quantitative insights into the roles of these two sources of noise for signaling systems where direct measurements are typically not possible.

Intrinsic variability between cells arises from the stochastic nature of biochemical reactions. Each reaction occurs at a random time, so, even if the molecular species concentrations are identical in every cell at the beginning of the experiment, their evolution will inevitably vary from one cell to another. This intrinsic variability has traditionally been modeled using stochastic simulation algorithms such as the Gillespie algorithm. Here, we aim to examine whether there exists parameter sets for which the stochasticity of the biochemical reactions induces a similar variability between cells to that observed in the experimental data. In order to infer such parameter sets, we use the linear noise approximation (LNA) (Elf and Ehrenberg, 2003; Ferm et al., 2008), which provides an explicit Gaussian likelihood for stochastic biochemical reactions (see Experimental Procedures).

Extrinsic sources of variability stem from all those elements of the “real system” that are not explicitly modeled; these typically include factors such as inherent differences between the cells in terms of protein concentrations at the start of the experiment, and other biophysical parameters. To capture such effects, we allow model parameters to differ between cells (Shahrezaei et al., 2008; Toni and Tidor, 2013): the parameters for each cell are drawn from a log-normal distribution (with means and variances that will be inferred from the data). The potential sources of extrinsic noise in the MEK-ERK system are differences in the reaction rates between cells in the (de-)phosphorylation process

of ERK, different initial concentrations of ERK and MEK, and differences in the upstream signaling cascades feeding into the MEK dynamics. The log-normal distribution has two advantages: it allows only positive values for reaction rates, and it allows parameters to vary over orders of magnitude if indicated by the data.

Using the Bayesian framework developed in the Experimental Procedures, we analyze the roles of intrinsic and extrinsic noise in the single-cell data. The resulting statistical model evidence indicates that the extrinsic noise best explains the data. The evolution of the obtained distributions for MEK and ERK are shown and compared to the data in Figure 4A: only the extrinsic noise model can explain the observed high levels of cell-to-cell variability.

Variation in initial conditions is also not sufficient to generate the observed cell-to-cell variability; this is easily seen by sampling different values for the initial concentration of the species involved in the MEK-ERK system according to a log-normal distribution with mean and variance (given by the inferred means and variances in the extrinsic noise case) and simulating the model with intrinsic noise for each of these initial conditions. The total variance, which is the sum of (1) the mean over the different initial conditions of the variance due to the intrinsic noise, and (2) the variance over the different initial conditions of the mean over the intrinsic variability, is shown in Figure 4B. This shows that the variance including variation in initial conditions does not differ appreciably from the variance of intrinsic noise alone.

In a biological system, we expect extrinsic and intrinsic sources of noise: the cells are likely to be different in terms of initial molecular concentrations and the biochemical reactions occur at random times. We therefore compare the variances of the observed molecular species under extrinsic noise alone with the total variances under both extrinsic and intrinsic noise. From Figure 4B, it is apparent that the contribution of intrinsic noise to the total variation is negligible.

In order to validate the model further, we consider the response of the MEK-ERK system to different stimuli; while the upstream dynamics will be different (different receptors and different upstream intermediates as well as dependence on stimulus strength and temporal pattern [Fujita et al., 2010; Toyoshima et al., 2012]), the core MEK-ERK model, if parameterized correctly, should capture the dynamics. Here, we therefore use the hyper-parameters inferred previously except for those that correspond to the upstream dynamics, which we inferred directly from the EGF and NGF time courses. We find that extrinsic noise model explains the response of the MEK-ERK system to stimulation by EGF (Figure 5A) and different NGF stimulus intensities (Figures 5B and S4). The model with extrinsic noise shows good qualitative and quantitative agreement between model predictions and the new data obtained for different stimulus. Thus, our extrinsic noise model is capable of predicting the response of the core MEK-ERK module to other stimuli than those used in the model development. EGF and NGF are known to give rise to very different downstream behavior (Santos et al., 2007), but the modular nature of MAPK signaling (Mody et al., 2009) means that the characterization of the MEK-ERK component for one input (a given concentration of NGF) already yields a model that can also explain the response to other stimuli.

Fluctuations in the Upstream Reactions and in the Degradation Rate of the Kinase Explain Most of the Cell-to-Cell Variability

Our Bayesian analysis allows us to assess directly which parameters differ most between cells. For each parameter, we have estimates of the coefficient of variation across cells, and the parameters that contribute most to the observed cell-to-cell variability are those for which the inferred coefficient of variation is consistently and significantly different from zero (see Figure S6). We find five strongly contributing factors: three model parameters (k_1, k_2, and k_{10}) and the two initial conditions that describe the level of background activity present in the cell at the point of stimulation. The degradation rate of active MEK (k_2) affects the steady-state levels of cell-to-cell variability; the role of degradation reactions in determining levels of noise (and thus cell-to-cell variability) has been previously studied (Komorowski et al., 2013). The pulse height, k_1, and the background upstream signal, k_{10}, jointly characterize the impact of the NGF stimulus and the upstream reactions on the evolution of active MEK (see Figure 1, top right). The origins of noise upstream of MEK are well documented and therefore expected; here, our focus is on how MEK-ERK core dynamics modulate such variability. In Figure 6A, we illustrate the predominant role that these three model parameters that describe the effect of the upstream signal (k_1, k_2, and k_{10}) have on the extent of cell-to-cell variability in this system.

To further investigate the role of the noise upstream to MEK compared to the noise in the core MEK-ERK module, we compare the joint distribution of the total amount of phosphorylated MEK and ERK when the system is simulated under the full extrinsic noise model or only varying the “driving” parameters k_1 and k_{10} between cells (see Figure 6B). Simply varying the “driving” parameters can explain the evolution of the variance and correlation between the two proteins; the joint distribution of active MEK and ERK is only slightly better captured when we consider all the factors in the full extrinsic noise model.

Impact of Cell-to-Cell Variability on Cellular Information Processing

We conclude our analysis by investigating the role that noise plays in mediating the response of the MEK-ERK module to external stimuli. We compute the mutual information between the total amount of phosphorylated MEK and ERK at different time points, simulating the system under extrinsic noise or varying only the parameters that seems to be related to most of the cellular variability (k_1, k_2, and k_{10})—all other model parameters are fixed to the inferred posterior mean values. We observe in Figure 7A that the presence of extrinsic noise decreases the level of transfer of information between the two species of interest. Thus, in a heterogeneous population of cells the statistical dependence between active MEK and ERK or, in other words, the expected information flowing through the MEK-ERK module is decreased. In light of the modest effect that within-module extrinsic noise appears to have on overall patterns of cell-to-cell variability in Figure 6, this profound change to the information transmission reliability might seem surprising. But it does reflect the complex behavior of the mutual information that can result from the interplay between system dynamics and extrinsic noise,

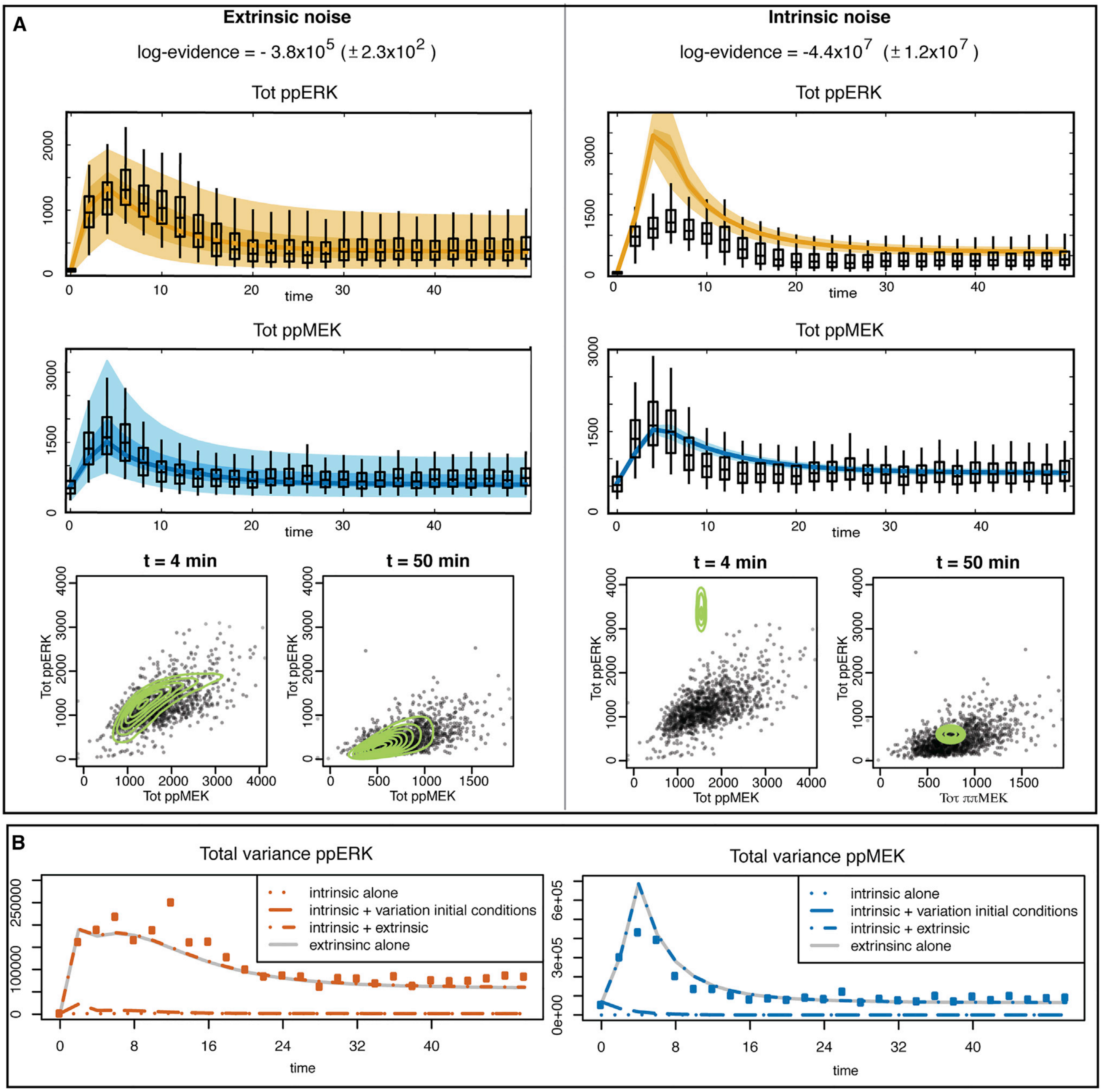

Figure 4. Intrinsic Noise Alone Cannot Explain the Observed Variability between Cells

(A) In the top part of the figure, the evolution of the inferred marginal distributions over the cell population for the two noise models are displayed and compared to the single-cell data distributions (boxplots). The lines represent the median of the distributions, while the shaded regions indicate the regions delimited by 5th and 95th percentiles (lighter regions) and the one delimited by 25th and 75th percentiles (darker regions). The medians and percentiles shown here are the average of the medians and percentiles computed for 1,000 sets of parameters sampled from the posterior distribution. The logarithm of the evidence is shown for both noise models; these are strongly supportive of the extrinsic noise models. In the bottom part of the figure, the inferred joint distributions of the total amount of phosphorylated MEK and ERK over the cell population (contour green line) for the two noise models are displayed and compared to the single-cell data distributions (gray dots) for two time points (4 and 50 min).

(B) Temporal evolution of the predicted variances over the cell population for the intrinsic noise model alone (dotted lines), the intrinsic noise model combined with a variation in initial conditions between cells (dashed lines), the intrinsic noise together with extrinsic noise (dash-dot lines), and the extrinsic noise alone (gray continuous line) show that the contribution of intrinsic noise is negligible. The squared dots represent the measured variance of the concentration of the two proteins over the cell populations.

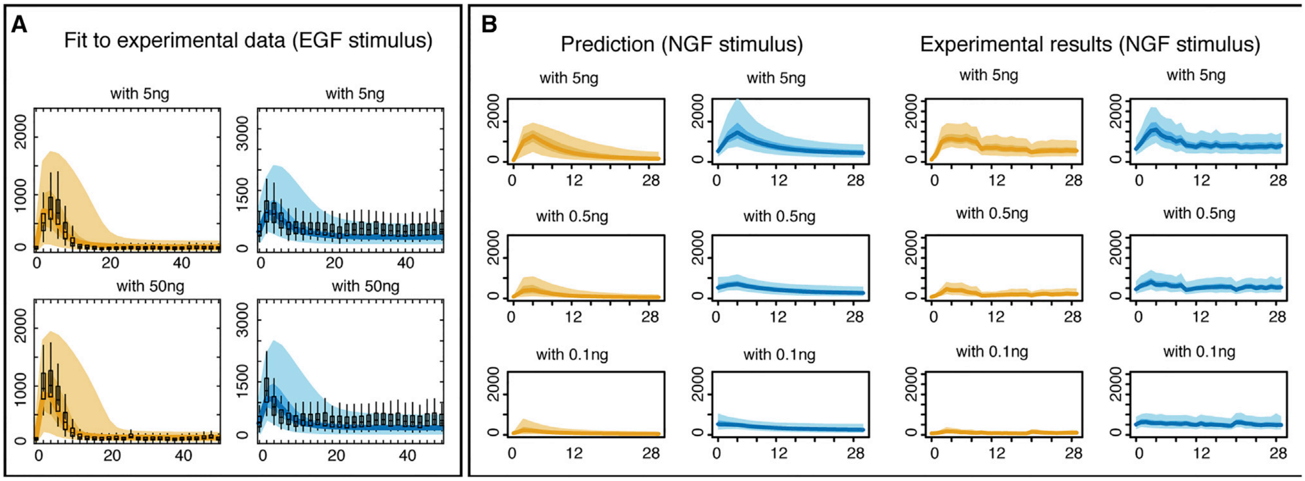

Figure 5. Prediction of the Impact of Growth Factor on Cell-to-Cell Variability

(A) Evolution of the inferred distributions of the total amount of phosphorylated ERK and MEK in response to stimulation by EGF with two levels of intensity. The hyper-parameters for the initial conditions and the reaction rates are fixed to the previously estimated values (using the single-cell data in response to NGF stimulus), whereas the hyper-parameters describing the impact of the stimulus and upstream signals on the kinase are inferred here separately. The single-cell data distributions (boxplots) are compared to the inferred distributions (the lines represent the median of the distributions, while the shaded regions indicate the regions delimited by 5th and 95th percentiles for the lighter regions and the one delimited by 25th and 75th percentiles for the darker regions).

(B) The predictions for the behavior of the total amount of phosphorylated ERK and MEK under the extrinsic noise model (left columns) are compared to experimental measurements (right columns) for different level of NGF intensity. The solid lines indicate the median value, while the shaded regions indicate the regions delimited by 5th and 95th percentiles (lighter zones) and by 25th and 75th percentiles (darker zones).

and which has been demonstrated theoretically elsewhere (Mc Mahon et al., 2015). In other words, variability in the upstream signals is faithfully transmitted (active ERK is approximately proportional to active MEK) if we ignore variability in the other factors. But when this is taken into account, this relationship becomes less well defined and at the population level the flow of information through MEK-ERK is radically decreased.

To follow on from this, we analyze the level of cell-to-cell variability in the system's output (i.e., the total amount of phosphorylated ERK) as a function of how variable the inputs (captured by the transient and sustained upstream intensities, k_1 and k_{10}, and their respective variances over the cell population $\sigma^2_{k_1}$ and $\sigma^2_{k_{10}}$) are. We simulate system output for given values of $\sigma^2_{k_1}$ and $\sigma^2_{k_{10}}$ and compute the ratio

$$\lambda\left(\sigma_{k_1},\sigma_{k_{10}},t\right)=\frac{s\left(\sigma_{k_1},\sigma_{k_{10}},t\right)}{s\left(\sigma^*_{k_1},\sigma^*_{k_{10}},t\right)},$$

where $s(\sigma_{k_1},\sigma_{k_{10}},t)$ is the SD of the output at time t (see Figure 7B). Note that $\sigma^*_{k_1}=\sqrt{\mu_{k_1}}$ and $\sigma^*_{k_{10}}=\sqrt{\mu_{k_{10}}}$ are the maximum values of these SD, where μ_{k_1} and $\mu_{k_{10}}$ are the means over the cell population for, respectively, k_1 and k_{10}. The ratio $\lambda(\sigma_{k_1},\sigma_{k_{10}},t)$ quantifies the change in the level of cell-to-cell variability in the system's output as the noise level in the input is decreased.

In the first instance, we assume that only the input signal strengths (k_1 and k_{10}) vary between cells. The evolution of $\lambda(\sigma_{k_1},\sigma_{k_{10}},t)$ over time when varying the variances $\sigma^2_{k_1}$ and $\sigma^2_{k_{10}}$ is shown in Figure 7C (left column). Before t = 8 min, $\lambda(\sigma_{k_1},\sigma_{k_{10}},t)$ increases with $\sigma^2_{k_1}$, whereas $\sigma^2_{k_{10}}$ has no impact on $\lambda(\sigma_{k_1},\sigma_{k_{10}},t)$. Conversely, after $t = 24$ min, $\lambda(\sigma_{k_1},\sigma_{k_{10}},t)$ increases with $\sigma^2_{k_{10}}$ but $\sigma^2_{k_1}$ no longer affects output variability. Thus variability in active ERK abundance across the cell population is initially strongly influenced by the variability in pulse height, k_1, and subsequently by the variability in the sustained or background signal, k_{10}.

To investigate further the effect of the variability in all model parameters on cellular information processing, we also simulate the system under extrinsic noise (varying all model parameters between cells) and compute once more $\lambda(\sigma_{k_1},\sigma_{k_{10}},t)$ for different signal variabilities. It is apparent from Figure 7C (right column) that, under the extrinsic noise model, the level of cell-to-cell variability in the system's output remains substantially high even when the variability in the system's input has been decreased considerably ($\lambda \simeq 0.45$ when σ_{k_1} and $\sigma_{k_{10}}$ are divided by 20). Again the presence of extrinsic noise weakens the efficiency of signal transduction.

DISCUSSION

In this study, we have used quantitative image cytometry to elucidate the causes of population heterogeneity in the MAPK signaling cascade and presented a comprehensive analysis of cell-to-cell variability in the activation dynamics of the MEK-ERK system to environmental stimuli. With a reliable model for the (de-)phosphorylation mechanisms in hand, we were able to dissect the nature of the cell-to-cell variability inherent in the data. Recent models for ERK phosphorylation proposed in the literature (Ortega et al., 2006; Sturm et al., 2010; Harrington et al., 2013; Ferrell and Ha, 2014; Voliotis et al., 2014) allow for very rich dynamics, and a priori it is therefore impossible to

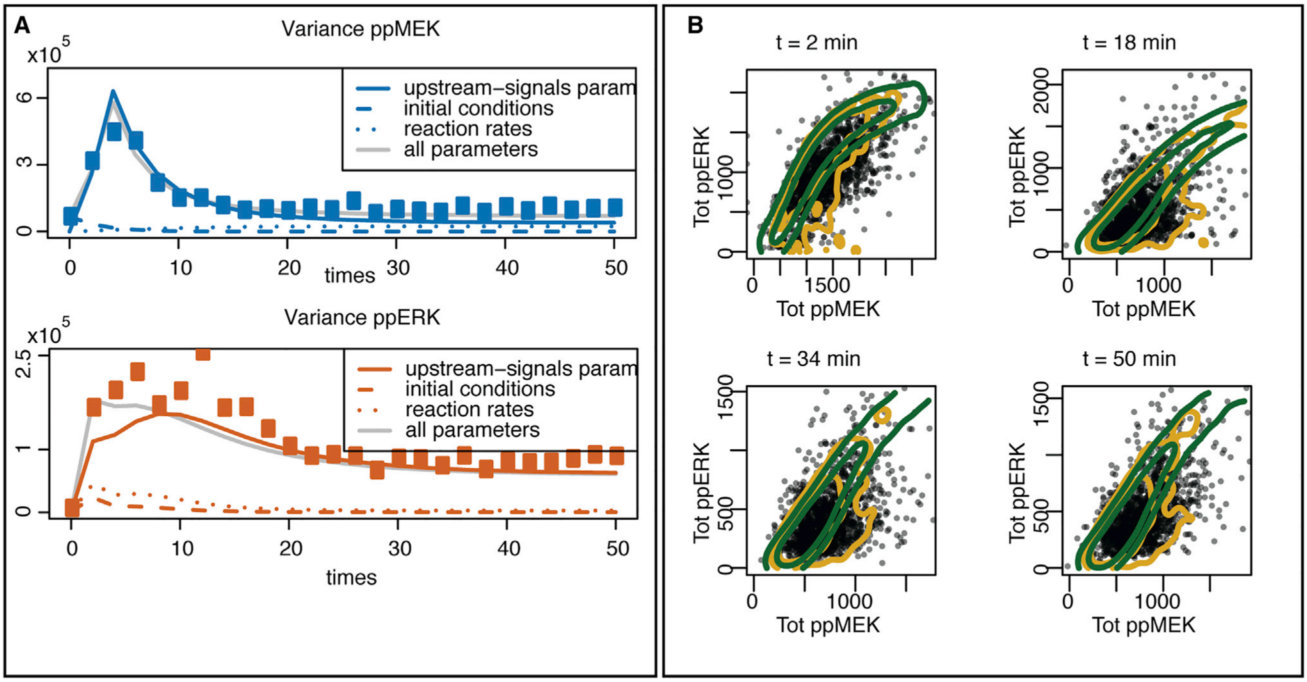

Figure 6. Factors Contributing to Cell-to-Cell Variability

(A) Evolution of the predicted variances when only some of the model parameters vary from one cell to another: either the parameters that describe the effect of the upstream signals (continuous lines), the parameters controlling the initial conditions (dashed lines), or the reaction rates (dotted lines). The light gray continuous line is the predicted variance when all model parameters differ between cells. The squared dots represent the measured variance of the concentration of the two proteins over the cell populations.

(B) Comparison of the observed (black dots) and the simulated joint distribution of the concentration of ppERK and ppMEK at different time points. The lines indicate the contour of the joint distribution when the system is simulated under the full extrinsic noise model (yellow) or only varying the parameters k_1 and k_{10} between cells (green).

make an appeal to the large number of MEK, ERK, and other molecules present in the eukaryotic cell, in order to rule out a role for intrinsic noise. The statistical framework developed for this study, however, gives a clear verdict in favor of extrinsic noise as the dominant factor for the observed cell-to-cell variability in the MEK-ERK system.

With the primary role of extrinsic noise established, we analyzed the contributions to cell-to-cell variability that arise from the different extrinsic sources of noise. Upstream of MEK many potential sources of noise and cell-to-cell variability have been identified in the literature, and our framework captures their contribution. In this study, the focus has been on the contributions to noise that arise from within the MEK-ERK module—this should be taken as akin to establishing the noise characteristics of, e.g., a transistor in electronics. We do find that there is considerable extrinsic variability in the module dynamics. However, the overall contribution of these within-module extrinsic sources of noise to the total dynamics quantitatively studied here are smaller than the upstream sources' contributions.

We then investigated how this extrinsic noise interferes with signal transduction. Our analysis shows that the overall joint distribution of phosphorylated MEK and ERK can be understood largely in terms of the upstream noise. The full model that accounts for extrinsic variability both upstream and within-module can be argued to capture more of the nuances seen in the empirical data but otherwise does not differ very much. However, these two scenarios have profoundly different impact on the ability of the MEK-ERK module to transmit upstream information to the activity profile of phosphorylated ERK: without extrinsic noise in the core module, variable (including noisy) signals would be faithfully reproduced downstream. But the extrinsic variability in the module parameters distorts these signals and leads to a drastic reduction in the mutual information between incoming signal and ERK activity.

Our results can be interpreted in two ways: we may simply regard the MEK-ERK module as poorly engineered as its behavior depends on the cellular context, or we may view this as a bet hedging (Stumpf et al., 2002; Kussell and Leibler, 2005) strategy, which poises different cells to respond differentially to stimulation, thereby reducing the risk of an inappropriate population wide response to noisy signals. In development and tissue homeostasis (Rué and Martinez Arias, 2015) (and in regenerative medicine), it may be important to find ways to regulate population-level behavior; e.g., using inter- and intra-cellular feedback mechanisms that control cell-to-cell variability further (Michailovici et al., 2014).

The study presented here is based on experiments carried out in PC12 cell lines (Greene and Tischler, 1976), which, unlike in vitro setups, provide the cell physiological context. The activity of upstream and downstream processes affecting ERK may

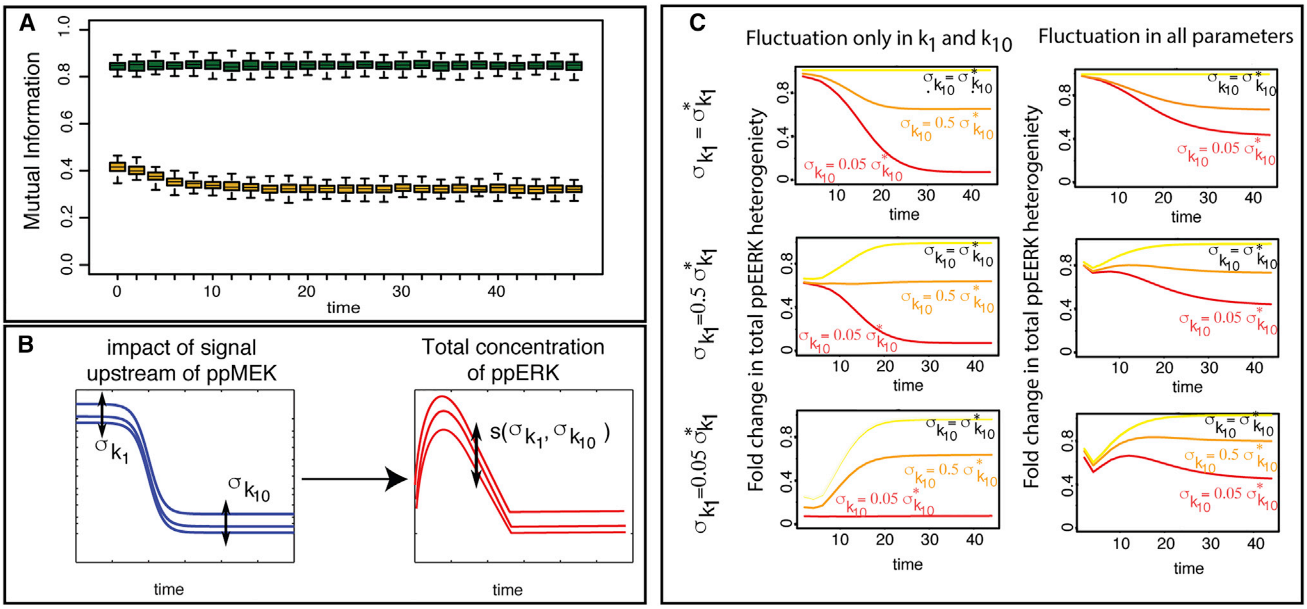

Figure 7. Information Processing of the MEK/ERK Module

(A) Mutual information between ppMEK and ppERK when the system is simulated under the full extrinsic noise model (yellow) or only varying the parameters k_1 and k_{10} between cells (green).

(B) The variability in the impact of reactions upstream ppMEK is summarized by the variances $\sigma^2_{k_1}$ and $\sigma^2_{k_{10}}$. The level of cell-to-cell variability in the total concentration of ppERK at time t depends on $\sigma^2_{k_1}$ and $\sigma^2_{k_{10}}$ and is denoted by $s(\sigma_{k_1}, \sigma_{k_{10}}, t)$.

(C) Illustration of the behavior of $\lambda(\sigma_{k_1}, \sigma_{k_{10}}, t)$ for decreasing values of σ_{k_1} and $\sigma_{k_{10}}$ — the red corresponding to the smallest and the yellow to the maximum SD . This subfigure is divided in two parts: in the left column, only parameters k_1 and k_{10} vary between cells (all other model parameters are fixed to their mean value), whereas in the right column the full extrinsic noise model is considered where all model parameters differ between cells. Each panel corresponds to a fixed value of σ_{k_1}, while each line corresponds to a fixed value of $\sigma_{k_{10}}$.

depend on cell type; this has, for example, been shown for nuclear shuttling, where even subtle differences between different cell lines can affect, e.g., the activity of nuclear ERK (Harrington et al., 2012). Our deliberate focus on the core MEK-ERK dynamics is less prone to such strong cell-type specificity over the timescales considered, whereas the potential of feedback from either ERK or any of its many downstream targets onto the MAPK cascade or proteins further upstream should be carefully considered in different cell types. The additional richness in behavior that such feedback (Ortega et al., 2006; Sturm et al., 2010) or explicit consideration of nuclear shuttling (Harrington et al., 2013) of ERK and MEK can induce warrants further investigation (Ozaki et al., 2010); here, over the time course considered, and in light of the data available such effects are marginal, but this may change as longer or more complex temporal stimulation patterns are considered. At the single-cell level, both feedback and shuttling are therefore clearly worth of further investigation.

It is important to keep in mind that no model will ever be able to contain all the constituent parts of any biological system of any real-world relevance. Therefore, extrinsic noise will always be an issue for modeling molecular and cellular systems. There are practical limitations to the current approach; notably it is not possible to fully describe correlations among the different extrinsic sources of noise (the number of parameters that we would have to estimate is simply too large); for example, interactions between kinases and phosphatases (Swain and Siggia, 2002) in MAPK may shape the response dynamics and such interactions are hard to capture in the extrinsic noise model. We believe, however, that the in silico approach developed here can serve to highlight such factors and may therefore be a guide to deciding which system aspects ought to be modeled explicitly. By pinpointing the sources of extrinsic noise, which are typically not obvious a priori, sound statistical modeling is able to provide deeper mechanistic insights and highlight where a model ought to be extended, or whether this is indeed necessary.

EXPERIMENTAL PROCEDURES

Experimental Data Collecting Process

The concentrations of molecular species were measured using quantitative image cytometry (QIC) (Ozaki et al., 2010; Saito et al., 2013). PC12 cells were seeded at a density of 10^4 cells per well in 96-well poly-L-lysine-coated glass-bottomed plates (Thermo Fisher Scientific). 24 hr after seeding, the medium was replaced with DMEM containing 25 mM HEPES and 0.1% of BSA. 18 hr after serum starvation, the stimulus is applied by replacing the starvation serum with a medium containing the stimulant (5 or 0.5 or 0.1 ng/ml). Our setup carries out stimulation in an incubator and achieved 1-min interval stimulation at 37°C under 5% CO_2 in saturated air humidity. The cells are then fixed with 4% paraformaldehyde for 10 min and immunostained. Cells were subjected to QIC analysis with mouse anti-ppERK1/2 Sigma-Aldrich M8159 antibody and rabbit anti-pMEK1/2 Cell Signaling Technology 9121. Note that anti-pMEK antibody detects both singly (pS217 or

pS221 alone) and doubly (pS217/221) phosphorylated MEK. Mouse monoclonal anti-ERK antibody (#4696, Cell Signaling Technology) and rabbit polyclonal anti-ERK antibody(#9102, Cell Signaling Technology) were used in Figure S2.

All images were analyzed with Cell Profiler (Kamentsky et al., 2011). The nuclear region was identified based on Hoechst imaging, and the cellular region was identified based on CellMask-stained images going out from the nuclear region. Total cellular signal intensity in nuclear regions and cellular regions were measured for ppERK and pMEK, respectively. We used these intensities as the concentrations of molecules. We used the cellular region in pixels as the cell size and the intensity of CellMask in the cellular region as a measure of cell volume.

The antibody against pMEK used detects both MEK1 and MEK2, all isoforms that phosphorylates ERK. In this study, we assume that the total phosphorylated MEK corresponds to doubly phosphorylated MEK. It is noted that no current technology is available to quantify the amounts of singly and doubly phosphorylated MEK. The antibody against ppERK detects both ERK1 and ERK2, all isoforms that are expressed in PC12 cells. To the best of our knowledge, there is no functional difference between the isoforms for both MEK and ERK at least in PC12 cells. Therefore, we did not make distinction between isoforms in this study.

Parameter Inference and Model Evidence

We use a Bayesian approach in order to infer the parameters of the system (see Supplemental Experimental Procedures for a detailed list of the model parameters) and rank the candidate mechanistic models. Bayesian parameter inference is centered around the posterior probability distribution, $p(\theta \mid x^*)$, which strikes a compromise between prior knowledge, $p(\theta)$, about parameter vectors, θ, and the capacity of a parameter to explain the observed data, x^*, measured by the likelihood $p(x^* \mid \theta)$, via

$$p(\theta \mid x^*) = \frac{p(\theta)\, p(x^* \mid \theta)}{\int p(\tilde{\theta})\, p(x^* \mid \tilde{\theta}) d\tilde{\theta}} .$$

Here, we evaluate the posterior using a sequential Monte Carlo (SMC) sampler proposed by Del Moral et al. (2006), which is easily parallelized. The output of the algorithm is a set of weighted parameter vectors $\{\theta^{(i)}, \omega^{(i)}\}_{1 \le i \le N}$. Here the parameter vector associated to the highest weight is called the *inferred parameter vector*. Technical details about our implementation of the SMC sampler algorithm are given in Supplemental Experimental Procedures.

The SMC sampler algorithm also enables us to evaluate the *model evidence* (Kirk et al., 2013), which is the probability to observe the data x^* under the model M (given the alternative models considered),

$$p(x^* \mid \mathcal{M}) = \int p(\theta \mid x^*, \mathcal{M}) p(\theta \mid \mathcal{M}) d\theta .$$

The model evidence allows us to rank candidate models in terms of their ability to explain the observed data x^*: the best model is the one with the highest model evidence. In addition, the Bayes factor assesses the plausibility of two candidate models $\mathcal{M}_1$ and $\mathcal{M}_2$:

$$BF_{1,2} = \frac{p(x^* \mid \mathcal{M}_1)}{p(x^* \mid \mathcal{M}_2)} .$$

Whenever $BF_{1,2}$ is larger than 30, the evidence in favor of model $M1$ is considered very strong (Jeffreys, 1961). We use our own implementation of the SMC sampler algorithm in Python as well as an interface to simulate the models in a computational efficient manner using a graphics processing unit (GPU) accelerated ordinary differential equation (ODE) solver (Zhou et al., 2011) and a C++ ODE solver for stiff models (Hindmarsh et al., 2005).

Likelihood Functions

At each time point $t \in \mathrm{T} = \{0, 2, 4, \ldots 50\}$, the total amount of doubly phosphorylated MEK and ERK are measured in N_t different cells. We denote by $x^*_{i,t}$ and $y^*_{i,t}$ the concentration of the two proteins in the i-th cell, $1 \le i \le N_t$, and by $\{\overline{x}^*_t\}_{t \in \mathrm{T}}$ and $\{\overline{y}^*_t\}_{t \in \mathrm{T}}$ the observed average trajectories. In addition, we denote by $x_t(\theta)$ and $y_t(\theta)$ the solution of the system of ODE given the parameter vector θ at time t.

Assuming an independent Gaussian measurement error for each time point with constant variance v, the likelihood function for the average data measurements is

$$p\left(\{\overline{x}^*_t, \overline{y}^*_t\}_{t \in \mathrm{T}} \mid \theta\right) = \prod_{t \in \mathrm{T}} \phi\left(\overline{x}^*_t; x_t(\theta), v\right) \phi\left(\overline{y}^*_t; y_t(\theta), v\right),$$

where $\phi(.; m, v)$ is the probability density function of a normal distribution of mean m and variance v. The variance v is inferred simultaneously with the other parameters.

In order to derive the likelihood function in the intrinsic noise model efficiently (Golightly and Wilkinson, 2015), we use the linear noise approximation (LNA). The LNA provides a system of ODEs, which describes how the means and the variances of the molecular species vary over time. These equations are produced using the *StochSens* package (Komorowski et al., 2012). With $m^x_t(\theta)$, $m^y_t(\theta)$, $v^x_t(\theta)$, and $v^y_t(\theta)$ denoting the solutions of the ODEs describing the means and variances for the parameter θ at time t, the likelihood $p(\{x^*_{i,t}, y^*_{i,t}\}_{i,t} \mid \theta)$ is equal to

$$\prod_{t \in \mathrm{T}} \prod_{i=1}^{N_t} \phi\left(x^*_{i,t}; m^x_t(\theta), v^x_t(\theta)\right) \phi\left(y^*_{i,t}; m^y_t(\theta), v^y_t(\theta)\right).$$

Extrinsic noise is modeled by considering that each cell has a different set of parameters. The distribution of each parameter across the cell population is assumed to be log-normal. We assume that these distributions are independent and denote by μ_θ and ${\sigma_\theta}^2$ the vector of the means and variances of these distribution, respectively. Due to computational cost, we do not consider any correlation between the parameters. There is no closed-form expression for the probability $p(\{x^*_{i,t}, y^*_{i,t}\}_{i,t} \mid \mu_\theta, \sigma^2_\theta)$, and we use the so-called Unscented Transform (UT) (Silk et al., 2011), which, given the first two moments μ_θ and σ^2_θ of the distribution in the parameter space, provides an approximation of the evolution of the means and variances of the two species of interest.

We denote by $m^x_t(\mu_\theta, \sigma^2_\theta)$ and $m^y_t(\mu_\theta, \sigma^2_\theta)$ the resulting mean behaviors of the two species at time t, and by $v^x_t(\mu_\theta, \sigma^2_\theta)$ and $v^y_t(\mu_\theta, \sigma^2_\theta)$ the associated variances. Assuming that the concentration of the doubly phosphorylated MEK and ERK proteins are log-normally distributed, we obtain that the likelihood $p(\{x^*_{i,t}, y^*_{i,t}\}_{i,t} \mid \mu_\theta, \sigma^2_\theta)$ is

$$\prod_{t \in \mathrm{T}} \prod_{i=1}^{N_t} \psi\left(x^*_{i,t}; m^x_t(\mu_\theta, \sigma^2_\theta), v^x_t(\mu_\theta, \sigma^2_\theta)\right) \psi\left(y^*_{i,t}; m^y_t(\mu_\theta, \sigma^2_\theta), v^y_t(\mu_\theta, \sigma^2_\theta)\right).$$

Here $\psi(.; m, v)$ is the probability density function of a log-normal distribution with mean m and variance v. The Supplemental Experimental Procedures contains additional technical details on the computation and the UT algorithm.

Mutual Information

The mutual information between two species (ppERK and ppMEK) is computed based on measurements of the protein concentrations in single-cells at different time points (Mc Mahon et al., 2015). For each time point, we estimate the mutual information using a kernel density estimate of the joint distribution. We use a Gaussian kernel with a diagonal covariance matrix and marginal variances equal to $1.06\sigma N^{-1/5}$ where σ is the marginal variance of the data and N is the number of data points (Silverman, 1986).

SUPPLEMENTAL INFORMATION

Supplemental Information includes Supplemental Experimental Procedures and seven figures and can be found with this article online at http://dx.doi.org/10.1016/j.celrep.2016.05.024.

AUTHOR CONTRIBUTIONS

S.F., C.P.B., S.K., and M.P.H.S. designed the study; T.K., K.K., T.T., T.W., and S.K. performed the data collection and initial processing; S.F., C.P.B.,

P.D.W.K., and S.S.M. performed modeling and statistical analysis; S.F., C.P.B., P.D.W.K., S.K., and M.P.H.S. wrote the paper; all authors approved the final version of the manuscript.

ACKNOWLEDGMENTS

P.D.W.K., T.K., S.K., and M.P.H.S. acknowledge financial support from the Human Frontiers Science Programme; S.F. was funded through an MRC Biocomputing fellowship; C.P.B. is a Wellcome Trust Career Development Fellow; S.F., C.P.B., P.D.W.K., S.K., and M.P.H.S. were also funded through a JST/BBSRC partnering award. M.P.H.S. is Royal Society Wolfson Research Merit Award holder.

Received: March 29, 2016
Revised: April 25, 2016
Accepted: May 4, 2016
Published: June 2, 2016

REFERENCES

Aoki, K., Yamada, M., Kunida, K., Yasuda, S., and Matsuda, M. (2011). Processive phosphorylation of ERK MAP kinase in mammalian cells. Proc. Natl. Acad. Sci. USA *108*, 12675–12680.

Ashall, L., Horton, C.A., Nelson, D.E., Paszek, P., Harper, C.V., Sillitoe, K., Ryan, S., Spiller, D.G., Unitt, J.F., Broomhead, D.S., et al. (2009). Pulsatile stimulation determines timing and specificity of NF-kappaB-dependent transcription. Science *324*, 242–246.

Balázsi, G., van Oudenaarden, A., and Collins, J.J. (2011). Cellular decision making and biological noise: from microbes to mammals. Cell *144*, 910–925.

Batchelor, E., Loewer, A., Mock, C., and Lahav, G. (2011). Stimulus-dependent dynamics of p53 in single cells. Mol. Syst. Biol. *7*, 488.

Bowsher, C.G., and Swain, P.S. (2012). Identifying sources of variation and the flow of information in biochemical networks. Proc. Natl. Acad. Sci. USA *109*, E1320–E1328.

Colman-Lerner, A., Gordon, A., Serra, E., Chin, T., Resnekov, O., Endy, D., Pesce, C.G., and Brent, R. (2005). Regulated cell-to-cell variation in a cell-fate decision system. Nature *437*, 699–706.

Del Moral, P., Doucet, A., and Jasra, A. (2006). Sequential Monte Carlo samplers. J. R. Stat. Soc. Ser. B. Stat. Methodol. *68*, 411–436.

Elf, J., and Ehrenberg, M. (2003). Fast evaluation of fluctuations in biochemical networks with the linear noise approximation. Genome Res. *13*, 2475–2484.

Elowitz, M.B., Levine, A.J., Siggia, E.D., and Swain, P.S. (2002). Stochastic gene expression in a single cell. Science *297*, 1183–1186.

Eser, U., Falleur-Fettig, M., Johnson, A., and Skotheim, J.M. (2011). Commitment to a cellular transition precedes genome-wide transcriptional change. Mol. Cell *43*, 515–527.

Ferm, L., Lötstedt, P., and Hellander, A. (2008). A hierarchy of approximations of the master equation scaled by a size parameter. J. Sci. Comput. *34*, 127–151.

Ferrell, J.E., Jr., and Bhatt, R.R. (1997). Mechanistic studies of the dual phosphorylation of mitogen-activated protein kinase. J. Biol. Chem. *272*, 19008–19016.

Ferrell, J.E., Jr., and Ha, S.H. (2014). Ultrasensitivity part II: multisite phosphorylation, stoichiometric inhibitors, and positive feedback. Trends Biochem. Sci. *39*, 556–569.

Fujita, K.A., Toyoshima, Y., Uda, S., Ozaki, Y., Kubota, H., and Kuroda, S. (2010). Decoupling of receptor and downstream signals in the Akt pathway by its low-pass filter characteristics. Sci. Signal. *3*, ra56.

Geva-Zatorsky, N., Rosenfeld, N., Itzkovitz, S., Milo, R., Sigal, A., Dekel, E., Yarnitzky, T., Liron, Y., Polak, P., Lahav, G., et al. (2006). Oscillations and variability in the p53 system. Mol. Sys. Biol., Published online June 13, 2006.

Golightly, A., and Wilkinson, D.J. (2015). Bayesian inference for Markov jump processes with informative observations. Stat. Appl. Genet. Mol. Biol. *14*, 169–188.

Greene, L.A., and Tischler, A.S. (1976). Establishment of a noradrenergic clonal line of rat adrenal pheochromocytoma cells which respond to nerve growth factor. Proc. Natl. Acad. Sci. USA *73*, 2424–2428.

Harrington, H.A., Komorowski, M., Beguerisse-Diaz, M., Ratto, G.M., and Stumpf, M.P.H. (2012). Mathematical modeling reveals the functional implications of the different nuclear shuttling rates of Erk1 and Erk2. Phys. Biol. *9*, 36001.

Harrington, H.A., Feliu, E., Wiuf, C., and Stumpf, M.M.P. (2013). Cellular compartments cause multistability and allow cells to process more information. Biophys. J. *104*, 1824–1831.

Hilfinger, A., and Paulsson, J. (2011). Separating intrinsic from extrinsic fluctuations in dynamic biological systems. Proc. Natl. Acad. Sci. USA *108*, 12167–12172.

Hindmarsh, A.C., Brown, P.N., Grant, K.E., Lee, S.L., Serban, R., Shumaker, D.E., and Woodward, C.S. (2005). SUNDIALS: suite of nonlinear and differential/algebraic equation solvers. ACM T. on Math. Software *31*, 363–396.

Inder, K., Harding, A., Plowman, S.J., Philips, M.R., Parton, R.G., and Hancock, J.F. (2008). Activation of the MAPK module from different spatial locations generates distinct system outputs. Mol. Biol. Cell *19*, 4776–4784.

Jeffreys, H. (1961). Theory of Probability (Oxford University Press).

Jeschke, M., Baumgärtner, S., and Legewie, S. (2013). Determinants of cell-to-cell variability in protein kinase signaling. PLoS Comput. Biol. *9*, e1003357.

Johnston, I.G. (2014). Efficient parametric inference for stochastic biological systems with measured variability. Stat. Appl. Genet. Mol. Biol. *13*, 379–390.

Kamentsky, L., Jones, T.R., Fraser, A., Bray, M.A., Logan, D.J., Madden, K.L., Ljosa, V., Rueden, C., Eliceiri, K.W., and Carpenter, A.E. (2011). Improved structure, function and compatibility for CellProfiler: modular high-throughput image analysis software. Bioinformatics *27*, 1179–1180.

Kiel, C., and Serrano, L. (2009). Cell type-specific importance of Ras-c-Raf complex association rate constants for MAPK signaling. Sci. Signal *2*, ra38.

Kirk, P., Thorne, T., and Stumpf, M.P. (2013). Model selection in systems and synthetic biology. Curr. Opin. Biotechnol. *24*, 767–774.

Komorowski, M., Finkenstädt, B., and Rand, D. (2010). Using a single fluorescent reporter gene to infer half-life of extrinsic noise and other parameters of gene expression. Biophys. J. *98*, 2759–2769.

Komorowski, M., Zurauskiene, J., and Stumpf, M.P.H. (2012). StochSens–Matlab package for sensitivity analysis of stochastic chemical systems. Bioinformatics *28*, 731–733.

Komorowski, M., Miękisz, J., and Stumpf, M.P. (2013). Decomposing noise in biochemical signaling systems highlights the role of protein degradation. Biophys. J. *104*, 1783–1793.

Kussell, E., and Leibler, S. (2005). Phenotypic diversity, population growth, and information in fluctuating environments. Science *309*, 2075–2078.

Mc Mahon, S.S., Lenive, O., Filippi, S., and Stumpf, M.P.H. (2015). Information processing by simple molecular motifs and susceptibility to noise. J. R. Soc. Interface *12*, 0597.

Michailovici, I., Harrington, H.A., Azogui, H.H., Yahalom-Ronen, Y., Plotnikov, A., Ching, S., Stumpf, M.P., Klein, O.D., Seger, R., and Tzahor, E. (2014). Nuclear to cytoplasmic shuttling of ERK promotes differentiation of muscle stem/progenitor cells. Development *141*, 2611–2620.

Miyanari, Y., and Torres-Padilla, M.-E. (2012). Control of ground-state pluripotency by allelic regulation of Nanog. Nature *483*, 470–473.

Mody, A., Weiner, J., and Ramanathan, S. (2009). Modularity of MAP kinases allows deformation of their signalling pathways. Nat. Cell Biol. *11*, 484–491.

Nelson, D.E., Ihekwaba, A.E., Elliott, M., Johnson, J.R., Gibney, C.A., Foreman, B.E., Nelson, G., See, V., Horton, C.A., Spiller, D.G., et al. (2004). Oscillations in NF-kappaB signaling control the dynamics of gene expression. Science *306*, 704–708.

Ortega, F., Garcés, J.L., Mas, F., Kholodenko, B.N., and Cascante, M. (2006). Bistability from double phosphorylation in signal transduction. Kinetic and structural requirements. FEBS J. *273*, 3915–3926.

Ozaki, Y., Uda, S., Saito, T.H., Chung, J., Kubota, H., and Kuroda, S. (2010). A quantitative image cytometry technique for time series or population analyses of signaling networks. PLoS ONE *5*, e9955.

Paszek, P., Ryan, S., Ashall, L., Sillitoe, K., Harper, C.V., Spiller, D.G., Rand, D.A., and White, M.R.H. (2010). Population robustness arising from cellular heterogeneity. Proc. Natl. Acad. Sci. USA *107*, 11644–11649.

Piala, A.T., Humphreys, J.M., and Goldsmith, E.J. (2014). MAP kinase modules: the excursion model and the steps that count. Biophys. J. *107*, 2006–2015.

Pujadas, E., and Feinberg, A.P. (2012). Regulated noise in the epigenetic landscape of development and disease. Cell *148*, 1123–1131.

Quaranta, V., and Garbett, S.P. (2010). Not all noise is waste. Nat. Methods *7*, 269–272.

Roberts, P.J., and Der, C.J. (2007). Targeting the Raf-MEK-ERK mitogen-activated protein kinase cascade for the treatment of cancer. Oncogene *26*, 3291–3310.

Rué, P., and Martinez Arias, A. (2015). Cell dynamics and gene expression control in tissue homeostasis and development. Mol. Syst. Biol. *11*, 792.

Saito, T.H., Uda, S., Tsuchiya, T., Ozaki, Y., and Kuroda, S. (2013). Temporal decoding of MAP kinase and CREB phosphorylation by selective immediate early gene expression. PLoS ONE *8*, e57037.

Santos, S.D.M., Verveer, P.J., and Bastiaens, P.I.H. (2007). Growth factor-induced MAPK network topology shapes Erk response determining PC-12 cell fate. Nat. Cell Biol. *9*, 324–330.

Santos, S.D.M., Wollman, R., Meyer, T., and Ferrell, J.E.J., Jr. (2012). Spatial positive feedback at the onset of mitosis. Cell *149*, 1500–1513.

Sasagawa, S., Ozaki, Y.-i., Fujita, K., and Kuroda, S. (2005). Prediction and validation of the distinct dynamics of transient and sustained ERK activation. Nat. Cell Biol. *7*, 365–373.

Schoeberl, B., Eichler-Jonsson, C., Gilles, E.D., and Müller, G. (2002). Computational modeling of the dynamics of the MAP kinase cascade activated by surface and internalized EGF receptors. Nat. Biotechnol. *20*, 370–375.

Schröter, C., Rué, P., Mackenzie, J.P., and Martinez Arias, A. (2015). FGF/MAPK signaling sets the switching threshold of a bistable circuit controlling cell fate decisions in embryonic stem cells. Development *142*, 4205–4216.

Selimkhanov, J., Taylor, B., Yao, J., Pilko, A., Albeck, J., Hoffmann, A., Tsimring, L., and Wollman, R. (2014). Systems biology. Accurate information transmission through dynamic biochemical signaling networks. Science *346*, 1370–1373.

Shahrezaei, V., Ollivier, J.F., and Swain, P.S. (2008). Colored extrinsic fluctuations and stochastic gene expression. Mol. Sys. Biol. *4*.

Silk, D., Kirk, P.D., Barnes, C.P., Toni, T., Rose, A., Moon, S., Dallman, M.J., and Stumpf, M.P.H. (2011). Designing attractive models via automated identification of chaotic and oscillatory dynamical regimes. Nat. Commun. *2*, 489.

Silverman, B.W. (1986). Density Estimation for Statistics and Data Analysis (CRC Press).

Spencer, S.L., Gaudet, S., Albeck, J.G., Burke, J.M., and Sorger, P.K. (2009). Non-genetic origins of cell-to-cell variability in TRAIL-induced apoptosis. Nature *459*, 428–432.

Spiller, D.G., Wood, C.D., Rand, D.A., and White, M.R.H. (2010). Measurement of single-cell dynamics. Nature *465*, 736–745.

Stumpf, M.P.H., Laidlaw, Z., and Jansen, V.A.A. (2002). Herpes viruses hedge their bets. Proc. Natl. Acad. Sci. USA *99*, 15234–15237.

Sturm, O.E., Orton, R., Grindlay, J., Birtwistle, M., Vyshemirsky, V., Gilbert, D., Calder, M., Pitt, A., Kholodenko, B., and Kolch, W. (2010). The mammalian MAPK/ERK pathway exhibits properties of a negative feedback amplifier. Sci. Signal. *3*, ra90.

Swain, P.S., and Siggia, E.D. (2002). The role of proofreading in signal transduction specificity. Biophys. J. *82*, 2928–2933.

Swain, P.S., Elowitz, M.B., and Siggia, E.D. (2002). Intrinsic and extrinsic contributions to stochasticity in gene expression. Proc. Natl. Acad. Sci. USA *99*, 12795–12800.

Takahashi, K., Tanase-Nicola, S., and ten Wolde, P.R. (2010). Spatio-temporal correlations can drastically change the response of a MAPK pathway. Proc. Natl. Acad. Sci. USA *107*, 2473–2478.

Toni, T., and Tidor, B. (2013). Combined model of intrinsic and extrinsic variability for computational network design with application to synthetic biology. PLoS Comput. Biol. *9*, e1002960.

Toni, T., Ozaki, Y., Kirk, P., Kuroda, S., and Stumpf, M.P.H. (2012). Elucidating the in vivo phosphorylation dynamics of the ERK MAP kinase using quantitative proteomics data and Bayesian model selection. Mol. Biosyst. *8*, 1921–1929.

Toyoshima, Y., Kakuda, H., Fujita, K.A., Uda, S., and Kuroda, S. (2012). Sensitivity control through attenuation of signal transfer efficiency by negative regulation of cellular signalling. Nat. Commun. *3*, 743.

Uda, S., Saito, T.H., Kudo, T., Kokaji, T., Tsuchiya, T., Kubota, H., Komori, Y., Ozaki, Y., and Kuroda, S. (2013). Robustness and compensation of information transmission of signaling pathways. Science *341*, 558–561.

Voliotis, M., Perrett, R.M., McWilliams, C., McArdle, C.A., and Bowsher, C.G. (2014). Information transfer by leaky, heterogeneous, protein kinase signaling systems. Proc. Natl. Acad. Sci. USA *111*, E326–E333.

Zhou, Y., Liepe, J., Sheng, X., Stumpf, M.P.H., and Barnes, C. (2011). GPU accelerated biochemical network simulation. Bioinformatics *27*, 874–876.

Cell Reports, Volume *15*

Supplemental Information

Robustness of MEK-ERK Dynamics and Origins

of Cell-to-Cell Variability in MAPK Signaling

Sarah Filippi, Chris P. Barnes, Paul D.W. Kirk, Takamasa Kudo, Katsuyuki Kunida, Siobhan S. McMahon, Takaho Tsuchiya, Takumi Wada, Shinya Kuroda, and Michael P.H. Stumpf

Supplemental Figures

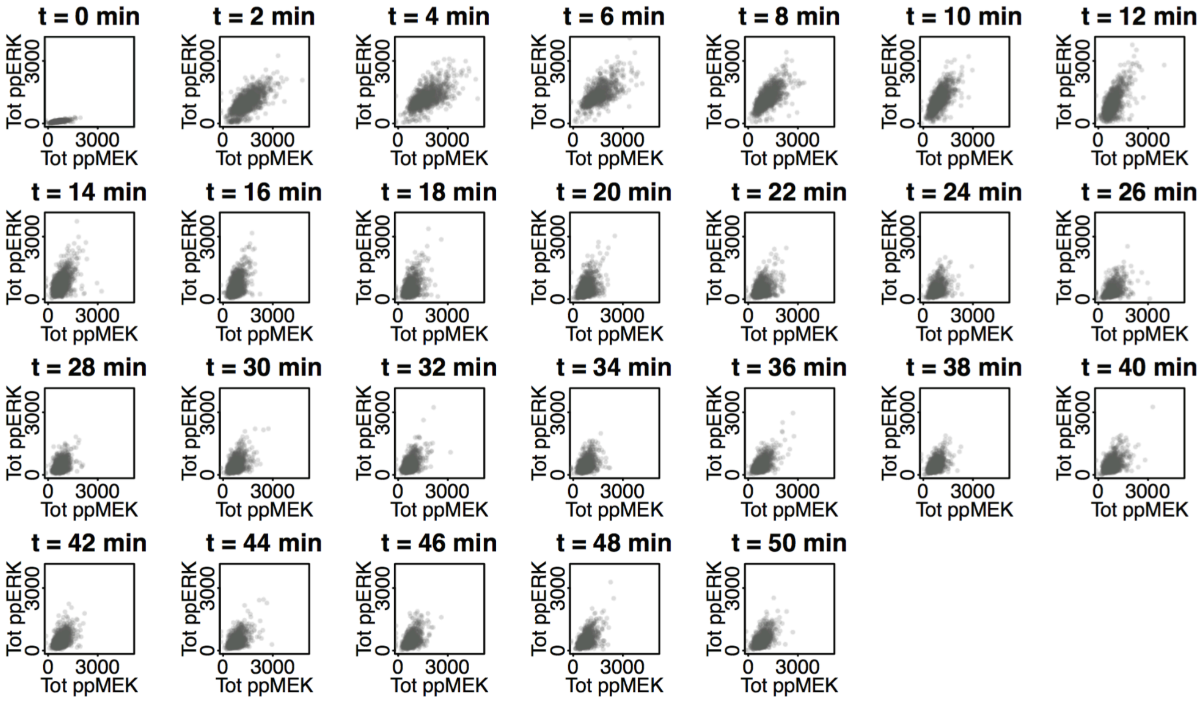

Figure S1: **Measurement of the evolution of the joint protein distributions of phosphorylated MEK and phosphorylated ERK. (Related to Figure 2B)** The joint distribution of total phosphorylated MEK and ERK every two minutes. Each dot corresponds to a single cell.

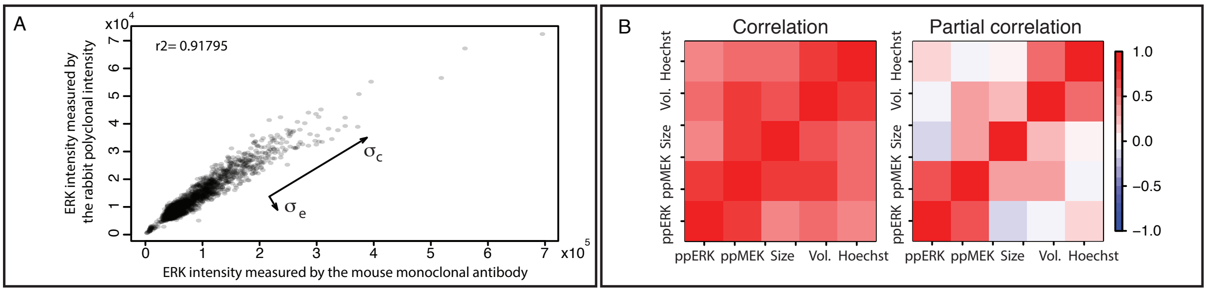

Figure S2: **Elucidating the origin of cell-to-cell variability. (Related to Figure 2) (A)** Estimation of experimental noise (Uda et al., 2013). Correlation of signal intensity of the total amount of ERKs measured by the two different antibodies. Each dot denotes the signal intensity of a single cell. We measured the total amount of ERKs by double-staining with mouse monoclonal and rabbit polyclonal antibodies. The correlation coefficient, r was 0.92. The cell-to-cell variation and experimental noise correspond to the variation along the major axis (σ_c) and that orthogonal to the major axis (σ_e), respectively. Copyright permission from Science. **(B)** In addition to the experimental measurements for the total amount of doubly phosphorylated ERK and MEK our assay also obtained measurements for cell-size, cell volume and Hoechst intensity in each cell. We computed the correlation and partial correlations between these 5 measurements using the R package GeneNet (Schäfer et al., 2001). Partial correlation is a much more powerful measure of statistical dependencies than correlation as has been discussed in detail by several authors (Kolaczyk, 2009; Schäfer and Strimmer, 2005; Thorne et al., 2013); it allows to measure dependences between each pairs of variables conditional on all other variables. Here, we compute the partial correlation between every pair of variables given the three other variables as controlling variables. For example, the partial correlation between the total amount of doubly phosphorylated ERK and MEK is the correlation between the residuals resulting from the linear regression of the total amount of doubly phosphorylated ERK and MEK respectively given the cell size, the cell volume and the Hoechst intensity. Both plots are on the same scale, see colour bar.

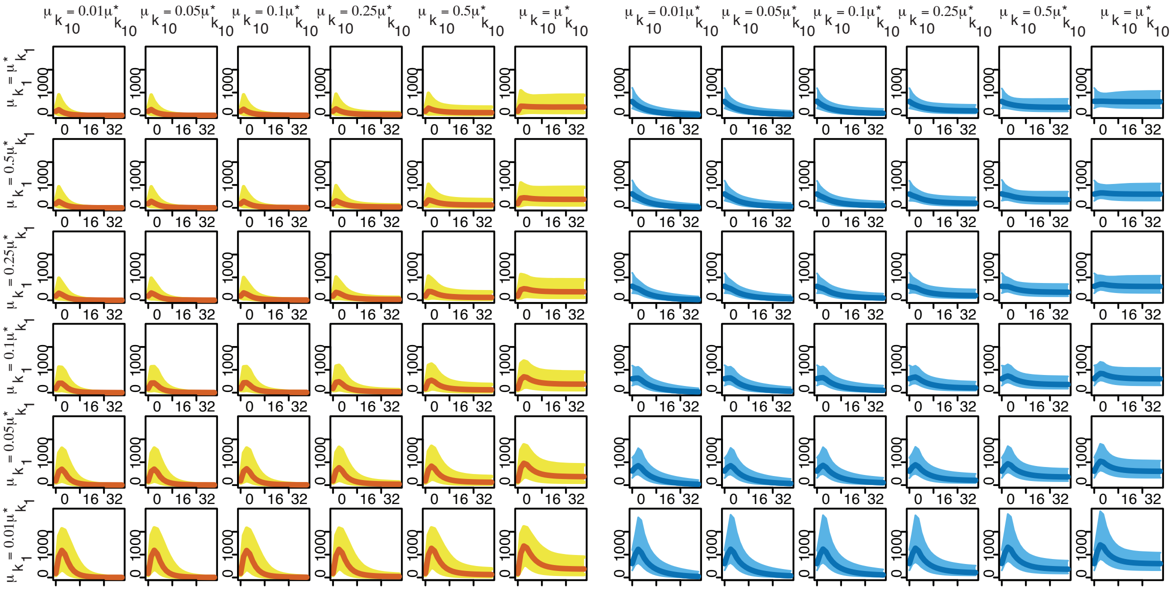

Figure S3: **Prediction of the impact of NGF intensity on cell-to-cell variability under the extrinsic noise model. (Related to Figure 5)** Predicted total amounts (medians and predicted 90% credible intervals) of doubly phosphorylated ERK (orange) and MEK (blue) under the extrinsic noise model are shown for decreasing values of the hyper-parameters μ_{k_1} and $\mu_{k_{10}}$. The reference hyper-parameters, $\mu^*_{k_1}$ and $\mu^*_{k_{10}}$, are the one inferred from the data.

Supplemental Experimental Procedure

Contents

S1 Description of the mechanistic models and the parameters **5**
S1.1 Systems of ordinary differential equations to model processive and distributive phosphorylation and dephosphorylation mechanisms 5
S1.2 Model parameters 9
S1.3 Observed species 10
S1.4 Model selection: distributive phosphorylation and dephosphorylation best explains the average behaviour 10

S2 Details on the implementation of the SMC sampler algorithm **12**

S3 The intrinsic noise model **13**
S3.1 Investigating the precision of the Linear Noise Approximation 13
S3.2 Efficient sampling of the parameter space 13

S4 The extrinsic noise model **15**
S4.1 Using Unscented Transform to derive a likelihood function for the extrinsic noise model 15
S4.2 Extrinsic noise: identification of model parameters that significantly vary between cells 16

S1 Description of the mechanistic models and the parameters

ERK activation requires phosphorylation at both its tyrosine and threonine phosphorylation sites by its cognate kinase MEK (Ferrell and Bhatt, 1997a), and two mechanisms for phosphorylation and dephosphorylation have been proposed (Ferrell and Bhatt, 1997b; Gunawardena, 2007; Toni et al., 2012), which are here referred to as processive and distributive. In the processive (P) mechanism, the kinase binds the protein and catalyzes the phosphorylation at both sites before dissociating from the doubly phosphorylated substrate protein. In the distributive (D) mechanism phosphorylation occurs in two steps where the kinase binds to the protein twice in order to phosphorylate the two site successively. Previous studies (Toni et al., 2012) have shown that *in vivo* phosphorylation (as well as dephosphorylation) occurs in a distributive way. In this section, we describe the four mechanistic models and confirm that this distributive mechanism best captures the observed average behavior in our data.

S1.1 Systems of ordinary differential equations to model processive and distributive phosphorylation and dephosphorylation mechanisms

In this section we derive the mathematical equations for different potential mechanistic models of the ERK (de-)phosphorylation process. In the following M denotes doubly phosphorylated MEK, which acts as a kinase, and E, pE and ppE denote un-, singly and doubly phosphorylated ERK, respectively; Pt represents the phosphotase activity. The phosphorylation and dephosphorylation processes of ERK involves these 5 species as well as the following protein complexes: $E \cdot M$, $pE \cdot M$, $ppE \cdot Pt$ and $pE \cdot Pt$.

The mechanistic models are described by the following reactions (see also Figure S4):

Phosphorylation

$$\text{Processive: } E + M \underset{k_4}{\overset{k_3}{\rightleftharpoons}} E \cdot M \xrightarrow{k_5} pE \cdot M \xrightarrow{k_6} ppE + M$$

$$\text{Distributive: } E + M \underset{k_4}{\overset{k_3}{\rightleftharpoons}} E \cdot M \xrightarrow{k_7} pE + M \underset{k_9}{\overset{k_8}{\rightleftharpoons}} pE \cdot M \xrightarrow{k_6} ppE + M$$

Dephosphorylation

$$\text{Processive: } ppE + Pt \underset{k_4^{'}}{\overset{k_3^{'}}{\rightleftharpoons}} ppE \cdot Pt \xrightarrow{k_5^{'}} pE \cdot Pt \xrightarrow{k_6^{'}} E + Pt$$

$$\text{Distributive: } ppE + Pt \underset{k_4^{'}}{\overset{k_3^{'}}{\rightleftharpoons}} ppE \cdot Pt \xrightarrow{k_7^{'}} pE + Pt \underset{k_9^{'}}{\overset{k_8^{'}}{\rightleftharpoons}} pE \cdot Pt \xrightarrow{k_6^{'}} E + Pt$$

The reaction rates are shown above or below the corresponding reactions.

In the derivation of the mechanistic models we incorporate the known experimental and biophysical constraints. First, since the concentration of active MEK depends on the upstream signals, its evolution is described by two additional reactions: a production reaction and a degradation reaction, which are given by

$$\emptyset \xrightarrow{g(k_1,T,k_{10},t)} M$$
$$M \xrightarrow{k_2} \emptyset,$$

where g is given by

$$g(k_1, T, k_{10}, t) = k_{10} + \frac{k_1}{\exp(t - T) + 1}\ .$$

In addition, some of the binding and un-binding reactions are reversible, whereas the phosphorylation and dephosphorylation reactions are not. The last assumption concerns the phosphorylation of ERK at the second

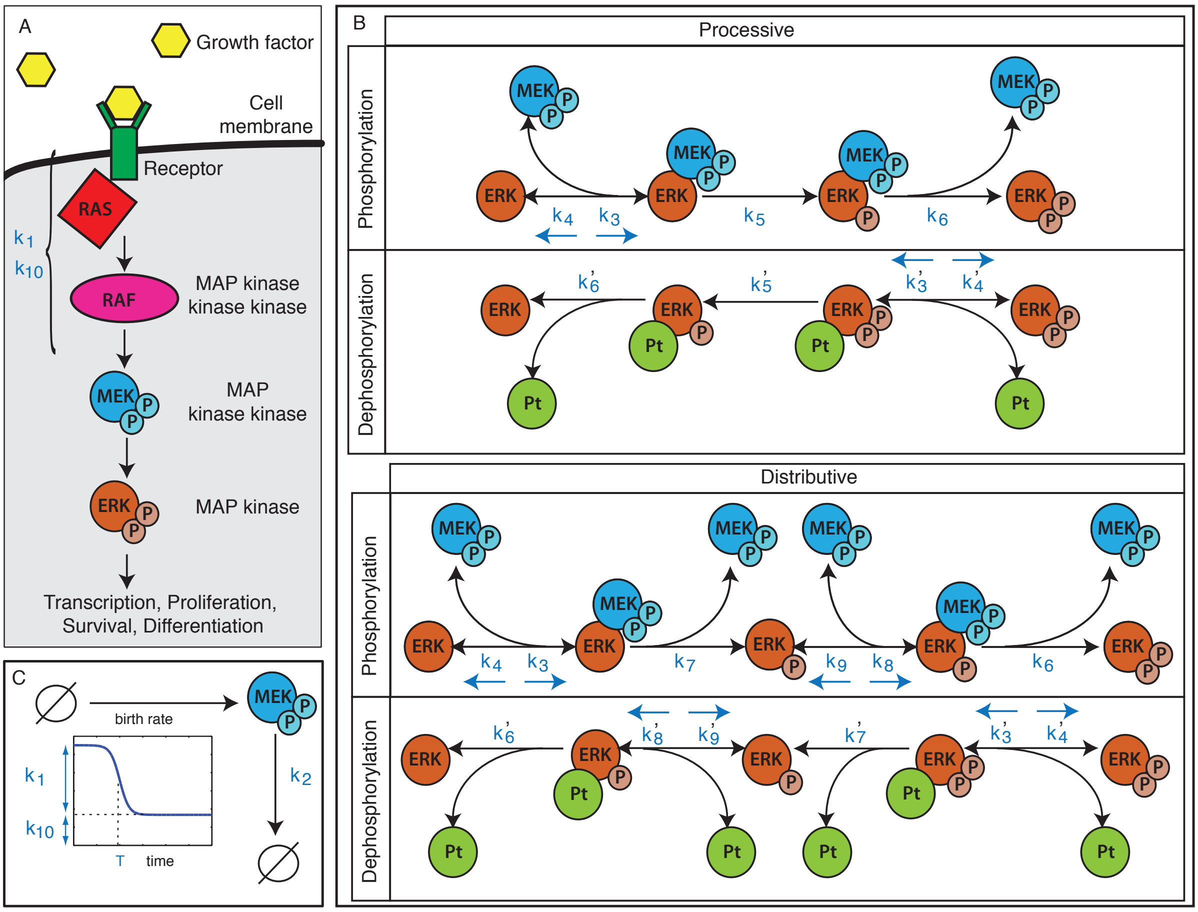

Figure S4: **MAPK Signalling. (Related to Figure 1) (A)** The RAS-RAF-ERK signal transduction cascade in response to a neural growth factor (NGF), which activates the membrane-bound GTPase (RAS); this leads to the activation of the RAF kinase and subsequently to the phosphorylation of MEK; active MEK in turn phosphorylates ERK. **(B)** Processive and distributive phosphorylation and dephosphorylation processes of ERK. Pt denotes the cognate ERK phosphatase. The reaction rates are shown next to their associated reactions. **(C)** The impact of the NGF stimulus and the upstream reactions on the evolution of the concentration of active MEK are modelled using a time dependent function which depends on three parameters: k_1 describes the pulse height, k_{10} the background signal and T the time at which the influence of the upstream reactions drops down. In addition active MEK is degraded with rate k_2.

site in the processive mechanism. We describe both this second phosphorylation and the unbinding of the double phosphorylated ERK to the active MEK in a single reaction. Moreover, since we work on time-scales where transcriptional responses can be ignored, we fix the total amounts (i.e. the sum of free and complex bound forms) of Pt, denoted by Pt_{tot} as well as the total amount of E, including all isophorms, denoted by E_{tot} (see Ozaki *et al.*, 2010).

By considering all possible combinations of processive (P) and distributive (D) phosphorylation and dephosphorylation reactions we can construct four possible models: DD, DP, PD and PP, where, for example, DP means distributive phosphorylation and processive dephosphorylation. The evolution of the concentrations (here denoted by $[A]_t$ for the concentration of species A at time t) of the 9 species is described by a set of ordinary differential equations.

<u>Distributive phosphorylation - Distributive dephosphorylation (DD)</u>

$$\begin{aligned}
\frac{d[M]_t}{dt} &= g(k_1, T, k_{10}, t) - k_2[M]_t - k_3[E]_t[M]_t + k_4[E.M]_t + k_6[pE.M]_t + k_7[E.M]_t \\
&\quad - k_8[pE]_t[M]_t + k_9[pE.M]_t \\
\frac{d[E]_t}{dt} &= -k_3[E]_t[M]_t + k_4[E.M]_t + k_6'[pE.Pt]_t \\
\frac{d[E.M]_t}{dt} &= k_3[E]_t[M]_t - k_4[E.M]_t - k_7[E.M]_t \\
\frac{d[pE]_t}{dt} &= k_7[E.M]_t - k_8[pE]_t[M]_t + k_9[pE.M]_t + k_7'[ppE.Pt]_t - k_8'[pE]_t[Pt]_t + k_9'[pE.Pt]_t \\
\frac{d[pE.M]_t}{dt} &= -k_6[pE.M]_t + k_8[pE]_t[M]_t - k_9[pE.M]_t \\
\frac{d[ppE]_t}{dt} &= k_6[pE.M]_t - k_3'[ppE]_t[Pt]_t + k_4'[ppE.Pt]_t \\
\frac{d[Pt]_t}{dt} &= -k_3'[ppE]_t[Pt]_t + k_4'[ppE.Pt]_t + k_6'[pE.Pt]_t + k_7'[ppE.Pt]_t - k_8'[pE]_t[Pt]_t + k_9'[pE.Pt]_t \\
\frac{d[ppE.Pt]_t}{dt} &= k_3'[ppE]_t[Pt]_t - k_4'[ppE.Pt]_t - k_7'[ppE.Pt]_t \\
\frac{d[pE.Pt]_t}{dt} &= -k_6'[pE.Pt]_t + k_8'[pE]_t[Pt]_t - k_9'[pE.Pt]_t
\end{aligned}$$

Distributive phosphorylation - Processive dephosphorylation (DP)

$$\frac{d[M]_t}{dt} = g(k_1, T, k_{10}, t) - k_2[M]_t - k_3[E]_t[M]_t + k_4[E.M]_t + k_6[pE.M]_t + k_7[E.M]_t - k_8[pE]_t[M]_t + k_9[pE.M]_t$$

$$\frac{d[E]_t}{dt} = -k_3[E]_t[M]_t + k_4[E.M]_t + k_6'[pE.Pt]_t$$

$$\frac{d[E.M]_t}{dt} = k_3[E]_t[M]_t - k_4[E.M]_t - k_7[E.M]_t$$

$$\frac{d[pE]_t}{dt} = k_7[E.M]_t - k_8[pE]_t[M]_t + k_9[pE.M]_t$$

$$\frac{d[pE.M]_t}{dt} = -k_6[pE.M]_t + k_8[pE]_t[M]_t - k_9[pE.M]_t$$

$$\frac{d[ppE]_t}{dt} = k_6[pE.M]_t - k_3'[ppE]_t[Pt]_t + k_4'[ppE.Pt]_t$$

$$\frac{d[Pt]_t}{dt} = -k_3'[ppE]_t[Pt]_t + k_4'[ppE.Pt]_t + k_6'[pE.Pt]_t$$

$$\frac{d[ppE.Pt]_t}{dt} = k_3'[ppE]_t[Pt]_t - k_4'[ppE.Pt]_t - k_5'[ppE.Pt]_t$$

$$\frac{d[pE.Pt]_t}{dt} = k_5'[ppE.Pt]_t - k_6'[pE.Pt]_t$$

Processive phosphorylation - Distributive dephosphorylation (PD)

$$\frac{d[M]_t}{dt} = g(k_1, T, k_{10}, t) - k_2[M]_t - k_3[E]_t[M]_t + k_4[E.M]_t + k_6[pE.M]_t$$

$$\frac{d[E]_t}{dt} = -k_3[E]_t[M]_t + k_4[E.M]_t + k_6'[pE.Pt]_t$$

$$\frac{d[E.M]_t}{dt} = k_3[E]_t[M]_t - k_4[E.M]_t - k_5[E.M]_t$$

$$\frac{d[pE]_t}{dt} = k_7'[ppE.Pt]_t - k_8'[pE]_t[Pt]_t + k_9'[pE.Pt]_t$$

$$\frac{d[pE.M]_t}{dt} = k_5[E.M]_t - k_6[pE.M]_t$$

$$\frac{d[ppE]_t}{dt} = k_6[pE.M]_t - k_3'[ppE]_t[Pt]_t + k_4'[ppE.Pt]_t$$

$$\frac{d[Pt]_t}{dt} = -k_3'[ppE]_t[Pt]_t + k_4'[ppE.Pt]_t + k_6'[pE.Pt]_t + k_7'[ppE.Pt]_t - k_8'[pE]_t[Pt]_t + k_9'[pE.Pt]_t$$

$$\frac{d[ppE.Pt]_t}{dt} = k_3'[ppE]_t[Pt]_t - k_4'[ppE.Pt]_t - k_7'[ppE.Pt]_t$$

$$\frac{d[pE.Pt]_t}{dt} = -k_6'[pE.Pt]_t + k_8'[pE]_t[Pt]_t - k_9'[pE.Pt]_t$$

Processive phosphorylation - Processive dephosphorylation (PP)

$$\frac{d[M]_t}{dt} = g(k_1, T, k_{10}, t) - k_2[M]_t - k_3[E]_t[M]_t + k_4[E.M]_t + k_6[pE.M]_t$$
$$\frac{d[E]_t}{dt} = -k_3[E]_t[M]_t + k_4[E.M]_t + k'_6[pE.Pt]_t$$
$$\frac{d[E.M]_t}{dt} = k_3[E]_t[M]_t - k_4[E.M]_t - k_5[E.M]_t$$
$$\frac{d[pE.M]_t}{dt} = k_5[E.M]_t - k_6[pE.M]_t$$
$$\frac{d[ppE]_t}{dt} = k_6[pE.M]_t - k'_3[ppE]_t[Pt]_t + k'_4[ppE.Pt]_t$$
$$\frac{d[Pt]_t}{dt} = -k'_3[ppE]_t[Pt]_t + k'_4[ppE.Pt]_t + k'_6[pE.Pt]_t$$
$$\frac{d[ppE.Pt]_t}{dt} = k'_3[ppE]_t[Pt]_t - k'_4[ppE.Pt]_t - k'_5[ppE.Pt]_t$$
$$\frac{d[pE.Pt]_t}{dt} = k'_5[ppE.Pt]_t - k'_6[pE.Pt]_t$$

In order to simulate these ODE systems one needs to determine the initial conditions for the concentrations of the 9 molecular species. We assume that the initial concentrations of the complexes $E.M$, pE, $pE.M$, $ppE.Pt$ and $pE.Pt$ are equal to 0. In addition, the initial conditions of the species E and ppE are constrained so that $[E]_t + [ppE]_t = E_{tot}$ and the molecular concentration of ppE and M are determined by the observation. Therefore, if we denote by ppE_0 and M_0 the initial concentrations of the two observed molecular species, we have the following initial conditions:

$$[M]_0 = M_0$$
$$[E]_0 = E_{tot} - ppE_0$$
$$[ppE]_0 = ppE_0$$
$$[Pt]_0 = Pt_{tot}$$

S1.2 Model parameters

In the rest of this supplemental material and in the manuscript, the term "model parameter" includes reaction rates, the 4 parameters describing the impact of upstream signals on active MEK (T, k_1, k_2 and k_{10}) and the 4 parameters related to the initial molecular concentration (M_0, ppE_0, E_{tot} and Pt_{tot}). In the following table, we summarise the number of parameters for each model.

Model	Reaction rates	Parameters related to upstream signals	Parameters related to initial concentrations	Total number of parameters
DD	12	4	4	20
DP	10	4	4	18
PD	10	4	4	18
PP	8	4	4	16

The model parameters are inferred using a Bayesian approach, therefore, a prior distribution over the parameter space need to be specified. We used uniform priors for each parameter based on the broader prior range proposed by Toni et al. (2012). Below we summarise the prior ranges used for each of the parameters (when performing parameter inference based on the average data, we set $M_0 = 636$ and $ppE_0 = 188$ according to the observed data).

Parameter	Lower limit	Upper limit	Parameter	Lower limit	Upper limit
k_1	0	100	k_{10}	1	2
T	200	400	k_2	2.10^{-3}	3.10^{-3}
k_3	0	1	k'_3	1	1000
k_4	0	1	k'_4	1	10^5
k_5	0	10^5	k'_5	0	10^5
k_6	100	1000	k'_6	0	100
k_7	1	500	k'_7	0	10
k_8	0	1	k'_8	0	1000
k_9	0	10	k'_9	0	10^5
E_{tot}	500	$1.8.10^4$	M_0	0	1000
Pt_{tot}	200	10^4	ppE_0	0	500

S1.3 Observed species

Quantitative image cytometry enables us to quantify the concentration of doubly phosphorylated ERK and MEK. More precisely, the total amount of free and complex bound forms of respectively doubly phosphorylated ERK and MEK are measured, i.e.

$$\text{total amount of doubly phosphorylated ERK} = ppE + ppE.Pt$$
$$\text{total amount of doubly phosphorylated MEK} = M + E.M + pE.M \ .$$

In the following and in the manuscript, we denote by

$$x_t = [ppE]_t + [ppE.Pt]_t$$

and

$$y_t = [M]_t + [E.M]_t + [pE.M]_t \ .$$

The quantities involved here are the solutions of the system of differential equations described above and typically depend on a vector of model parameter θ (see subsection S1.2).

S1.4 Model selection: distributive phosphorylation and dephosphorylation best explains the average behaviour

We use Bayesian parameter inference and model selection to determine which mechanism best captures the observed average behavior. Assuming an independent Gaussian measurement error for each time point with constant variance, we obtain the likelihood derived in *Experimental Procedure*. The best fits to the data for the four models are shown in Figure S5A. Although both the DD (distributive phosphorylation and dephosphorylation) and PD (processive phosphorylation and distributive dephosphorylation) models provide good fits to the data, the DD model receives much higher support by the Bayesian model ranking procedure (see Figure S5B). We will therefore base our analysis of the origins of cell-to-cell variability on this DD model with 20 model parameters including 12 reaction rates, 4 parameters describing the impact of the NGF stimulus and upstream signals and 4 parameters controlling the initial concentrations of the species involved in the ERK-MEK system.

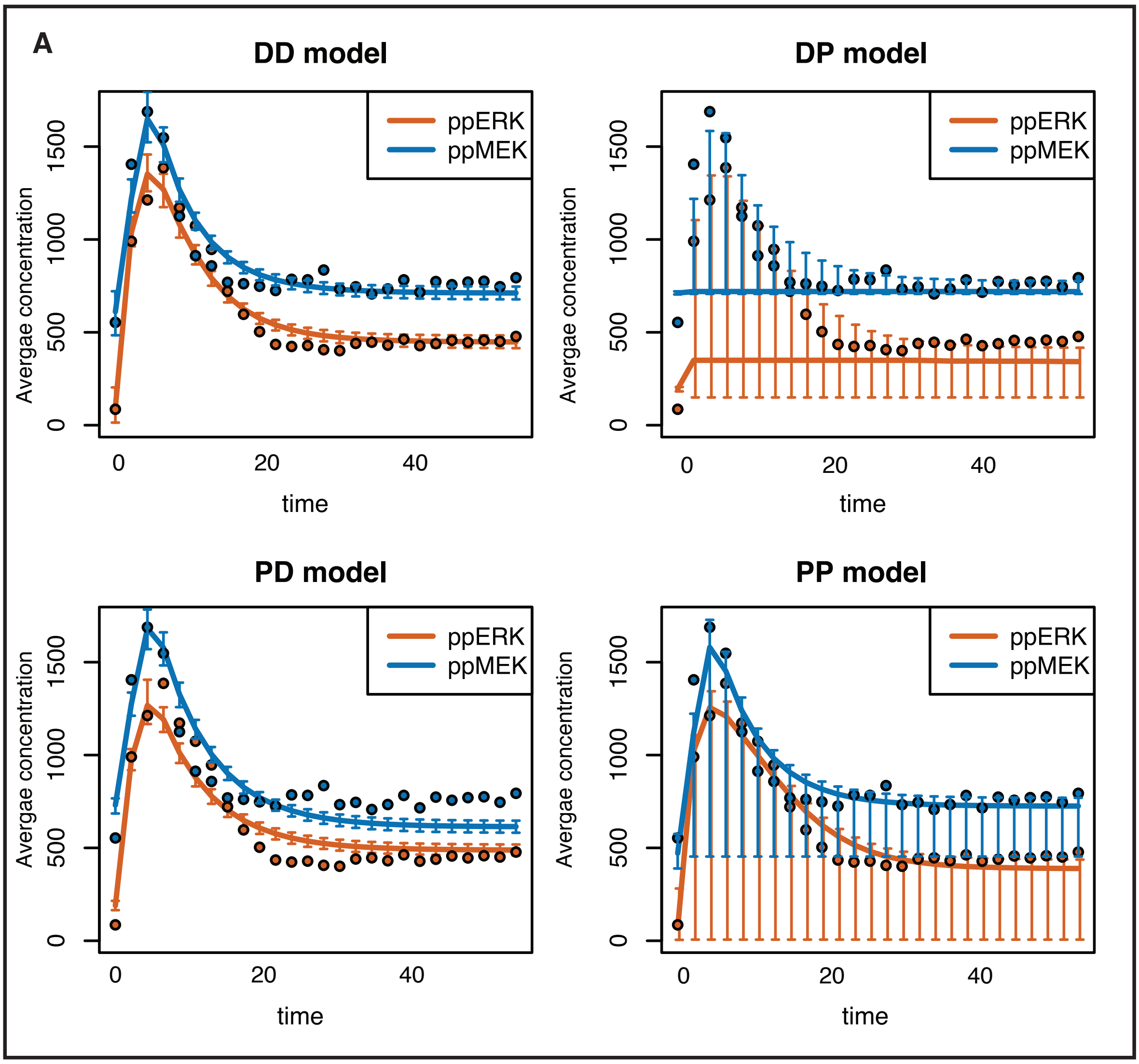

B

Model	log-evidence	Bayes factor wrt DD
DD	-227 (± 0.8)	
DP	-264 (±8)	1.2×10^{16}
PD	-260 (± 8)	2.2×10^{14}
PP	-245 (± 3)	6.5×10^{7}

Figure S5: **The distributive phosphorylation and dephosphorylation mechanism best explains the average behavior. (Related to Figure 1) (A)** Fits to the average data (dots) for the four mechanistic models. The lines represent the median of the evolution of the average concentration of the two species (total amount of doubly phosphorylated ERK in orange and total amount of doubly phosphorylated MEK in blue) and the error bars designate the 0.025 and 0.975 quantiles for a set of 1000 parameters sampled from the posterior distribution. Both the DD and the DP models provide good visual fits. **(B)** Model ranking through the evidence shows that the DD model has the highest log-evidence and is therefore more strongly supported by the average data.

S2 Details on the implementation of the SMC sampler algorithm

Parameter inference is performed using the Sequential Monte Carlo sampler algorithm proposed by Del Moral et al. (2006), which is emerging as a powerful alternative to conventional Markov chain Monte Carlo (MCMC) methods (Robert and Casella, 2004). The algorithm sequentially generates samples from the probability distributions, $p(\theta)^{1-\phi}p(\theta|x^*)^{\phi}$, for ϕ varying from 0 to 1, by sampling parameter vectors from the prior distribution, $p(\theta)$, and exploiting the likelihood function in order to guide the set of parameter vectors toward a region of high posterior probability. The exact version of the algorithm we use is detailed below (see Algorithm 1). We use $N = 10^4$ particles per population. To determine the next value of ϕ at the beginning of each population (step 5), we ensure that the effective sample size (ESS), which is equal to $\left(\sum_{n=1}^{N} \omega_a^{(n)}\right)^{-1}$ and only depends on the previous weighted population $\{(\theta_{a-1}^{(n)}, \omega_{a-1}^{(n)})\}_{1\leq n\leq N}$ as well as on ϕ and ϕ_{old}, is between 0.5 and 0.9. In addition, to perturb the particles (step 10), we use an MCMC kernel which consists of 5 steps of Metropolis Hasting perturbations with an adaptive multi-variate normal random-walk proposal (Del Moral et al., 2006).

Algorithm 1: The SMC sampler

Input: No. of particles per population N.
Output: Set of weighted particles $\{\theta_p^{(n)}, \omega_p^{(n)}\}_{1\leq n\leq N}$.
1 Initialise $a = 1$, $\phi = 0$;
2 Sample particles $\theta_1^{(n)}$ from prior ;
3 Set weights $\omega_1^{(n)} = \frac{1}{N}$;
4 **while** $\phi < 1$ **do**
5 Set $\phi_{old} = \phi$; a = a+1;
6 Determine next value of ϕ;
7 Resample particles $\{\theta_{a-1}^{(n)}\}$ from weighted multinomial distribution $\{(\theta_{a-1}^{(n)}, \omega_{a-1}^{(n)})\}$;
8 Reset weights $\omega_{a-1}^{(n)} = \frac{1}{N}, \forall n = 1, \ldots, N$;
9 **for** $1 \leq n \leq N$ **do**
10 Draw $\theta_a^{(n)} \sim K_a(\cdot \mid \theta_{a-1}^{(n)})$, where K_a is a MCMC kernel;
11 Update particle weight $\widetilde{\omega}_a^{(n)} = \omega_{a-1}^{(n)} \cdot p(\theta_{a-1}^{(n)}|x^*)^{\phi-\phi_{old}}$;
12 **end**
13 Normalise particle weights $\omega_a^{(n)} = \widetilde{\omega}_a^{(n)} / \sum_{m=1}^{N} \widetilde{\omega}_a^{(m)}$.
14 **end**

S3 The intrinsic noise model

The Linear Noise Approximation (LNA) is used to define a likelihood function in the case of the intrinsic noise model. It is a Gaussian approximation to Markov Jump processes defined by the Chemical Master Equation (Komorowski et al., 2009). Biochemical reactions are modelled through a stochastic dynamic model and the LNA provides us with equations for the average behaviour over the population of cells as well as the evolution of the variance and covariances with time. With $m_t^x(\theta)$, $m_t^y(\theta)$, $v_t^x(\theta)$ and $v_t^y(\theta)$ denoting the solutions of the ODEs describing the means and variances for the parameter θ at time t, the likelihood $p(\{x^*_{i,t}, y^*_{i,t}\}_{i,t}|\theta)$ is equal to

$$\prod_{t\in\mathbb{T}}\prod_{i=1}^{N_t}\Phi(x^*_{i,t}; m_t^x(\theta), v^x(\theta))\Phi(y^*_{i,t}; m_t^y(\theta), v^y(\theta))$$

where $\Phi(\cdot; m, v)$ is the probability density function of a normal distribution of mean m and variance v.

S3.1 Investigating the precision of the Linear Noise Approximation

In order to investigate the accuracy of the Linear Noise Approximation, we generated 100 trajectories simulating the DD model with the Gillespie algorithm (which took around 12 CPU days per trajectory), computed the evolution of the mean and the variance over the 100 trajectories and compared it to the solution of the ODE equations provided by the LNA. As can be seen in Figure S6A, the Linear Noise approximations of the evolution of the means and the variances are very accurate.

S3.2 Efficient sampling of the parameter space

In the main manuscript, we demonstrate that we can confidently implicate extrinsic noise as the dominant factor giving rise to cell-to-cell variability in the MEK/ERK module and that intrinsic noise does not explain the level of observed cell-to-cell variability. To substantiate this further (and to ensure that we explore the parameter space more widely during the parameter inference step of the intrinsic noise) we use Latin hyper-cube sampling (McKay et al., 1979) to generate a set of 10^6 parameter vectors using the Matlab function *lhsdesign.* and systematically analyse the evolution of the molecular concentrations of MEK and ERK for each of these parameters. Only 20 parameter vectors out of the 10^6 lead to stable solutions for which the obtained variances of doubly phosphorylated ERK and MEK is higher than 10^5 (at either 6 or 8 minutes after stimulation; but for none of these parameters do we observe a variance of doubly phosphorylated ERK that is anywhere close to the experimental observations.

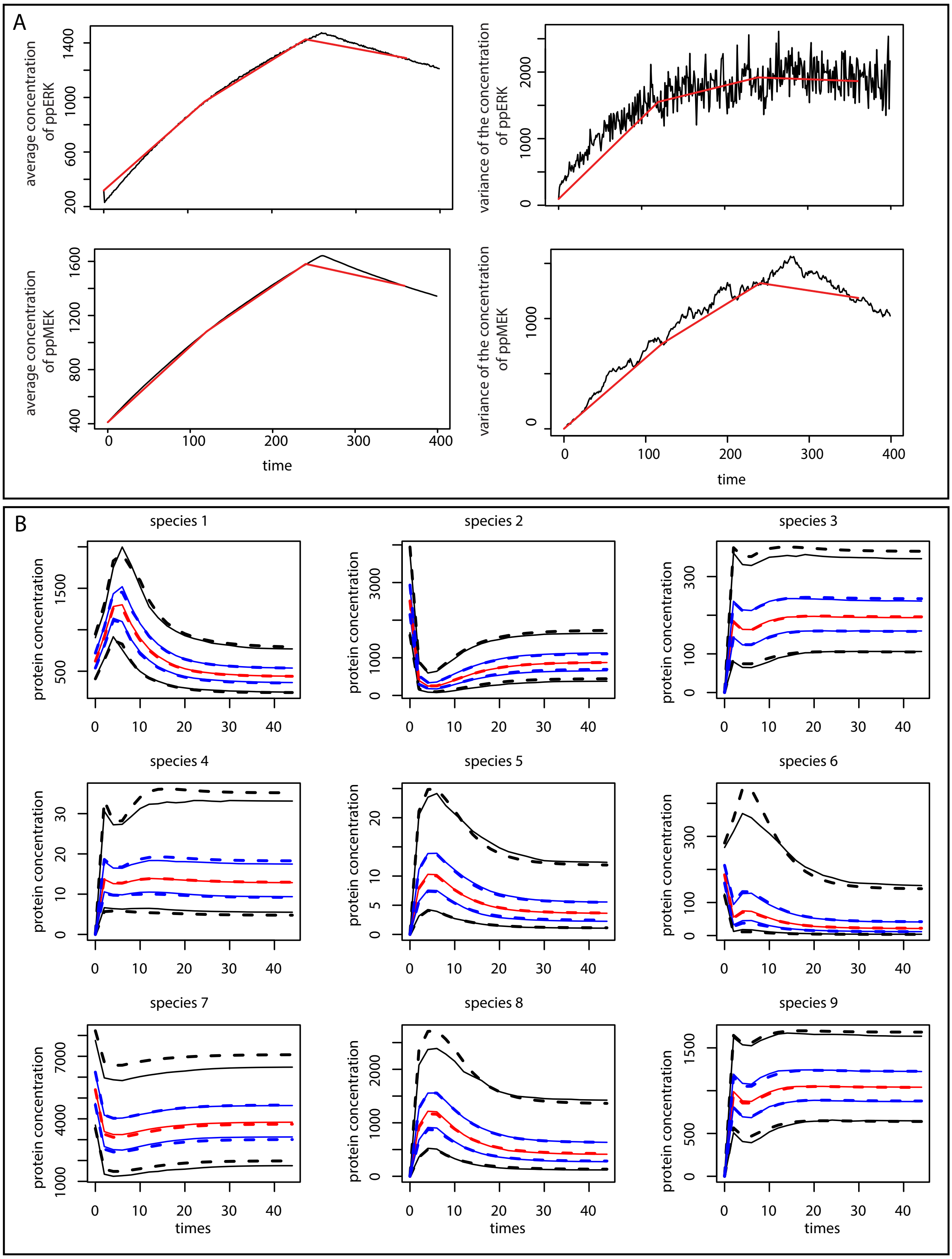

Figure S6: **Accuracy of likelihood approximations. (Related to Figures 3 and 4)** **(A)**The evolution of the mean and variance for the two molecular species of interest. The black lines corresponds to the mean and variances over the trajectories simulated using the Gillespie algorithm and the red lines are the approximation provided by the LNA. **(B)** Comparison of the distribution of the evolution of the protein concentrations (for all the 9 species) for one set of hyper-parameters given by the UT method and by a Monte-Carlo approximation with 1000 particles. Each plot is associated with one of the 9 species. The continuous lines correspond to the Monte-Carlo approximation and the dashed lines correspond to the UT approximation. The red lines represent the median, the blue lines represent the 0.25 and 0.75 quantiles and the black lines represent the 0.025 and the 0.975 quantiles of the distributions.

S4 The extrinsic noise model

S4.1 Using Unscented Transform to derive a likelihood function for the extrinsic noise model

To mathematically describe the cell-to-cell variability due to extrinsic noise a different parameter vector is associated with each cell. Recall that for each time $t \in \mathbb{T} = \{0, 2, 4, \ldots 50\}$, we measure the total amount of doubly phosphorylated MEK and ERK, denoted respectively by $x^*_{i,t}$ and $y^*_{i,t}$, in cells $1 \leq i \leq N_t$. Let denote by $\theta_{i,t}$ the vector of parameters associated to the i-th cell measured at time t. With a single measurement per cell, it is not possible to infer every single parameter $\theta_{i,t}$. Instead, we study the distribution of the parameters $\{\theta_{i,t}\}_{i,t}$; here, we consider a log-normal distribution with mean μ_θ and covariance matrix Σ_θ.

We aim at inferring the hyper-parameters μ_θ and Σ_θ of the constructed hierarchical model given the observed data $\{x^*_{i,t}, y^*_{i,t}\}_{i,t}$. Note that the model contains 20 parameters (see section S1.2) therefore μ_θ is of length 20 and Σ_θ is a 20×20 symmetric matrix. Due to the prohibitive computational cost of inferring these 210 values, we simplify the model by assuming that the covariance matrix Σ_θ is diagonal; we denote by σ^2_θ the vector containing the diagonal elements. The likelihood function is defined as follows

$$p(\{x^*_{i,t}, y^*_{i,t}\}_{i,t} | \mu_\theta, \sigma^2_\theta) = \prod_t \prod_i \int \mathbb{1}_{(x^*_{i,t}, y^*_{i,t}) = f(\theta_{i,t}, t)} p(\theta_{i,t} | \mu_\theta, \sigma^2_\theta) d\theta_{i,t} \tag{1}$$

where $f(\theta_{i,t}, t)$ describes the simulated concentration of the two species of interest at time t when simulating the model with parameter $\theta_{i,t}$; as described above $p(\theta_{i,t}|\mu_\theta, \sigma^2_\theta)$ is the density of a log-normal distribution with mean μ_θ and covariance matrix $\Sigma_\theta = \text{diag}(\sigma^2_\theta)$. This likelihood function can not be computed in closed-form and needs to be estimated. We propose to use the *Unscented Transform* (UT) (Julier, 2002) to approximate the two moments of the distribution $p(\{x^*_{i,t}, y^*_{i,t}\}_{i,t}|\mu_\theta, \Sigma_\theta)$. We denote by $m^x_t(\mu_\theta, \sigma^2_\theta)$ (resp. $m^y_t(\mu_\theta, \sigma^2_\theta)$) and $v^x_t(\mu_\theta, \sigma^2_\theta)$ (resp. $v^y_t(\mu_\theta, \sigma^2_\theta)$) the mean and the variance of this distributions as a function of the hyper–parameters μ_θ and σ^2_θ .

The UT is a mathematical tool, which allows us to approximate the moments of the output of a non-linear function given the moments of the input. The first step of the UT algorithm is to determine a set of weighted particles $\{\xi_j\}_j$, called *sigma points*, which capture both the mean μ_θ and the variances σ^2_θ. Since we assume a log-normal distribution over the parameter space, we consider the variable $\tilde{\theta}$, which is distributed according to a normal distribution with mean $\tilde{\mu}_\theta = \log(\mu_\theta) - 1/2 \log(\sigma^2_\theta/\mu^2_\theta + 1)$ and variance $\tilde{\sigma}^2_\theta = \log(\sigma^2_\theta/\mu^2_\theta + 1)$. Denoting by D the dimension of the parameter space, the sigma-points are defined as follows:

$$\begin{aligned}
\xi_0 &= \tilde{\mu}_\theta \\
\xi_j &= \tilde{\mu}_\theta + \alpha\sqrt{D + \kappa}\, [\tilde{\sigma}_\theta]_j && j = 1, \cdots, D \\
\xi_j &= \tilde{\mu}_\theta - \alpha\sqrt{D + \kappa}\, [\tilde{\sigma}_\theta]_j && j = D + 1, \cdots, 2D,
\end{aligned}$$

where $[\tilde{\sigma}_\theta]_j$ represents a vector full of zeros except on the j-th element which is equal to the j-the element of the vector $\tilde{\sigma}_\theta$. The sigma-point weights $\{w^m_j, w^v_j\}_{0 \leq j \leq 2D}$ are given by,

$$\begin{aligned}
w^m_0 &= \frac{\alpha^2(D + \kappa) - D}{\alpha^2(D + \kappa)} \\
w^v_0 &= \frac{\alpha^2(D + \kappa) - D}{\alpha^2(D + \kappa)} + 1 - \alpha^2 + \beta \\
w^m_j &= w^v_j = \frac{1}{2\alpha^2(D + \kappa)} && j = 1, \cdots, 2D\,.
\end{aligned}$$

The parameters κ, α and β may be chosen to control the positive definiteness of the covariance matrices, spread of the sigma-points and error in the kurtosis respectively (Silk, 2013). Here we use $\kappa = 0$, $\alpha = 0.7$ and $\beta = 2$.

Once the sigma-points have been determined, the ODE system is solved for each sigma-points separately. More precisely, for each $0 \leq j \leq 2D$, we solve the ODE system with the parameter $\exp(\xi_j)$, resulting in solutions $x_{j,t}$ and $y_{j,t}$. Assuming a log-normal distribution in the molecular concentration space, the means and variances of the distribution at each time point can then be computed as

$$m_t^x(\mu_\theta, \sigma_\theta^2) = \sum_{j=0}^{2D} w_j^m \log(x_{j,t})$$

$$m_t^y(\mu_\theta, \sigma_\theta^2) = \sum_{j=0}^{2D} w_j^m \log(y_{j,t})$$

$$v_t^x(\mu_\theta, \sigma_\theta^2) = \sum_{j=0}^{2D} w_j^v (\log(x_{j,t}) - m_t^x(\mu_\theta, \sigma_\theta^2))^2$$

$$v_t^y(\mu_\theta, \sigma_\theta^2) = \sum_{j=0}^{2D} w_j^v (\log(x_{j,t}) - m_t^y(\mu_\theta, \sigma_\theta^2))^2 \ .$$

Therefore the likelihood in equation (1) is approximated as follows

$$p(\{x_{i,t}^*, y_{i,t}^*\}_{i,t}|\mu_\theta, \sigma_\theta^2) = \prod_{t\in\mathbb{T}} \prod_{i=1}^{N_t} \Psi\left(x_{i,t}^*; m_t^x(\mu_\theta, \sigma_\theta^2), v_t^x(\mu_\theta, \sigma_\theta^2)\right) \Psi\left(y_{i,t}^*; m_t^y(\mu_\theta, \sigma_\theta^2), v_t^y(\mu_\theta, \sigma_\theta^2)\right)$$

where $\Psi(\cdot)$ is the pdf of a log-normal distribution.

In Figure S6B we compare the approximation of the likelihood function $p(\{x_{i,t}^*, y_{i,t}^*\}_{i,t}|\mu_\theta, \sigma_\theta^2)$ given by the UT algorithm to a Monte-Carlo approximation. In the Monte-Carlo approximation, 1000 set of parameters are sampled from log-normal distributions (with mean μ_θ and variance σ_θ^2). For each parameter set, we solve the system of ODE to obtain trajectories of every species in the system and then we compute the median and quantiles of the obtained trajectories.

S4.2 Extrinsic noise: identification of model parameters that significantly vary between cells

Under the extrinsic noise model, all model parameters differ between cells. In each cell every parameter, k, is drawn from a log-normal distribution with mean μ_k and variance σ_k^2 (called hyper-parameters). Our Bayesian inference procedure based on the single-cell data allows us to obtain posterior distributions for every hyper-parameters. For the DD model, there are 20 model parameters and therefore 40 hyper-parameters.

To investigate which parameters contribute most to the observed cell-to-cell variability, we analyse the posterior distribution of the coefficient of variation for each parameter (σ_k/μ_k for each k) shown in Figure S7. The coefficients of variation take value between 0 and 1. We distinguish 3 types of posterior distributions: (i) posterior distributions with a support that covers more than 60% of $[0, 1]$ (framed in blue), (ii) posterior distributions close to 0 (posterior framed in yellow has 0.25th percentile lower than 0.05 and a 0.75th percentile lower than 0.3), and (iii) the other posteriors distributions which are more tightly constrained and significantly different to 0 (framed in red). The parameters that contribute most to the observed cell-to-cell variability are those for which the posterior distribution of the coefficient of variation is consistently and significantly different from zero. Indeed, a posterior distribution of a coefficient of variation very close to 0 indicates that the model parameter does not need to vary between cells; a posterior of a coefficient of variation not constrained and including most of the $[0, 1]$ support demonstrates that the variation of this model parameter between cells is not crucial to explain the level of cell-to-cell variability. Therefore only the parameters framed in red in Figure S7 appear to play an important role in the cell-to-cell variability. These parameters are k_1, k_2, k_{10}, ppE_0 and M_0.

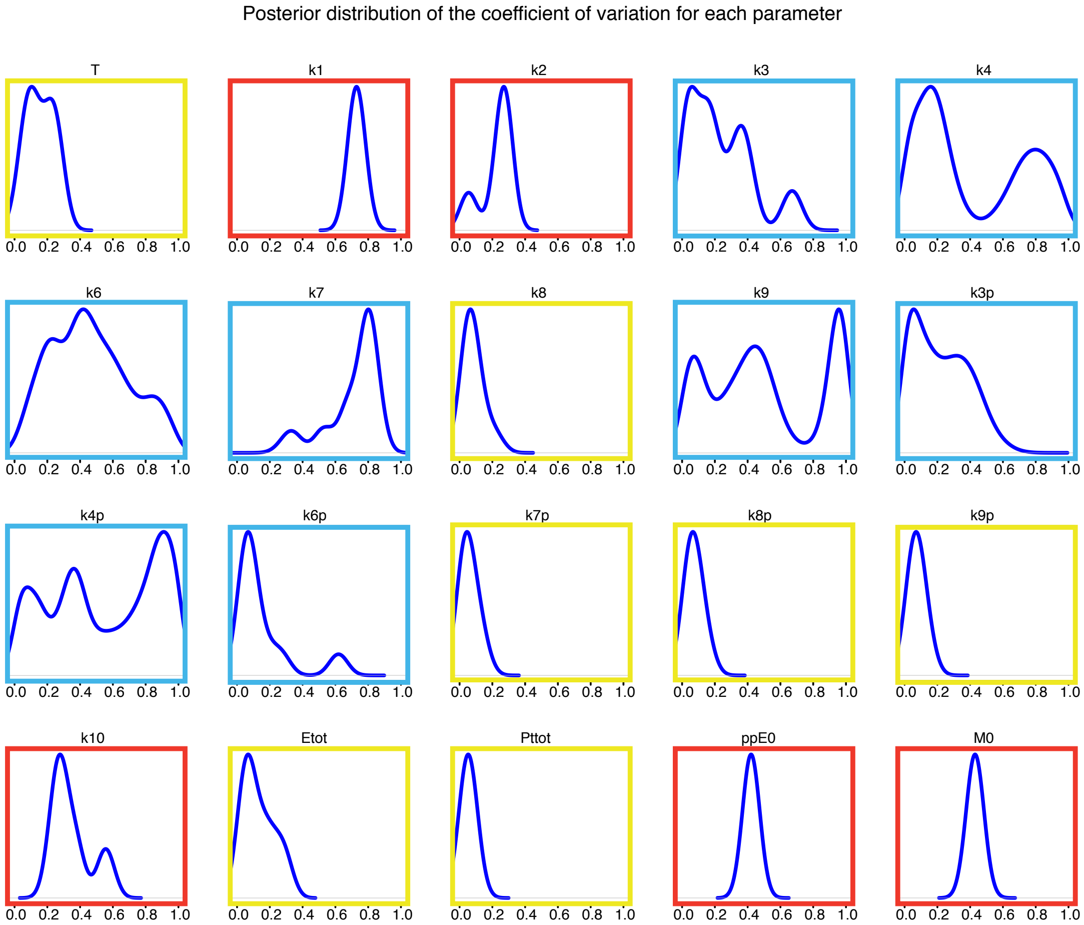

Figure S7: **Posterior distribution of coefficient of variation. (Related to Figures 4, 5 and 6)** The posterior distribution obtained using SMC sampler for the coefficient of variation of each parameter are shown here. We distinguish 3 types of posterior distributions: (i) posterior distributions with a support that covers more than 60% of $[0, 1]$ (framed in blue), (ii) posterior distributions close to 0 (posterior framed in yellow has 0.25th percentile lower than 0.05 and a 0.75th percentile lower than 0.3), and (iii) the other posteriors distributions which are more tightly constrained and significantly different to 0 (framed in red).

Supplemental References

Del Moral, P., Doucet, A. and Jasra, A. (2006). Sequential monte carlo samplers. Journal of the Royal Statistical Society: Series B (Statistical Methodology) *68*, 411–436.

Ferrell, J. E. and Bhatt, R. R. (1997a). Mechanistic studies of the dual phosphorylation of mitogen-activated protein kinase. Journal of Biological Chemistry *272*, 19008–19016.

Ferrell, J. E. and Bhatt, R. R. (1997b). Mechanistic studies of the dual phosphorylation of mitogen-activated protein kinase. Journal of Biological Chemistry *272*, 19008–19016.

Gunawardena, J. (2007). Distributivity and processivity in multisite phosphorylation can be distinguished through steady-state invariants. Biophysical journal *93*, 3828–3834.

Julier, S. J. (2002). The scaled unscented transformation. In American Control Conference, Proceedings of the IEEE vol. 6, pp. 4555–4559,.

Kolaczyk, E. D. (2009). Statistical Analysis Of Network Data: Methods And Models. Springer.

Komorowski, M., Finkenstädt, B., Harper, C. and Rand, D. (2009). Bayesian inference of biochemical kinetic parameters using the linear noise approximation. BMC bioinformatics *10*, 343.

McKay, M. D., Beckman, R. J. and Conover, W. J. (1979). Comparison of three methods for selecting values of input variables in the analysis of output from a computer code. Technometrics *21*, 239–245.

Robert, C. P. and Casella, G. (2004). Monte Carlo statistical methods, vol. 319,. Citeseer.

Schäfer, J., Opgen-Rhein, R. and Strimmer, K. (2001). Reverse engineering genetic networks using the GeneNet package. Journal of the American Statistical Association *96*, 1151–1160.

Schäfer, J. and Strimmer, K. (2005). A shrinkage approach to large-scale covariance matrix estimation and implications for functional genomics. Statistical applications in genetics and molecular biology *4*, Article32.

Silk, D. (2013). Unscented approaches to inference and design for systems and synthetic biology. PhD thesis, Imperial College London.

Thorne, T. W., Fratta, P., Hanna, M. G., Cortese, A., Plagnol, V., Fisher, E. M., Stumpf, M. P. H. and Stumpf, M. P. H. (2013). Graphical modelling of molecular networks underlying sporadic inclusion body myositis. Molecular Biosystems *9*, 1736–1742.

Toni, T., Ozaki, Y.-i., Kirk, P., Kuroda, S. and Stumpf, M. P. H. (2012). Elucidating the in vivo phosphorylation dynamics of the ERK MAP kinase using quantitative proteomics data and Bayesian model selection. Molecular BioSystems *8*, 1921–1929.

Uda, S., Saito, T. H., Kudo, T., Kokaji, T., Tsuchiya, T., Kubota, H., Komori, Y., Ozaki, Y.-i. and Kuroda, S. (2013). Robustness and compensation of information transmission of signaling pathways. Science *341*, 558–561.
